# Exercise dose and modality for blood pressure reduction in predominantly postmenopausal women: a dose-response network meta-analysis

**DOI:** 10.3389/fmed.2026.1918407

**Published:** 2026-08-12

**Authors:** Dongze Li, Jingxin Shi, Tianfu Yu, Jie Wang, Qiyan Jin

**Affiliations:** 1 School of Sports Science and Engineering, East China University of Science and Technology, Shanghai, China; 2 School of Physical Education, Northeast Normal University, Changchun, Jilin, China; 3 College of Physical Education, Henan Agricultural University, Zhengzhou, Henan, China; 4 College of Basic Medicine, Guangxi University of Chinese Medicine, Nanning, Guangxi, China

**Keywords:** blood pressure, exercise, hypertension, menopause, network meta-analysis

## Abstract

**Background:**

Blood pressure rises during the menopausal transition and postmenopause, yet exercise guidance seldom specifies the type and dose most effective for lowering it. We examined the dose-response relationship between exercise and blood pressure in trials enrolling peri- or postmenopausal women, while recognizing that the available evidence was predominantly derived from postmenopausal populations.

**Methods:**

We searched PubMed, Web of Science, Embase, and the Cochrane CENTRAL register from inception to March 2026 for randomized controlled trials of exercise interventions lasting at least 4 weeks in peri- or postmenopausal women. To limit dose misclassification, weekly dose was derived only from trials that reported exercise frequency, intensity, and session duration, expressed as METs-min/week and adjusted for attendance where this was reported. A Bayesian model-based dose-response network meta-analysis was fitted, with risk of bias assessed using RoB 2 and certainty of evidence using CINeMA.

**Results:**

Seventy-four trials involving 3,573 women (mean age, 59.5 years) were included; 70 exclusively enrolled postmenopausal women and four included perimenopausal women. Among eight candidate dose-response functions, the quadratic model yielded the lowest DIC and was retained as the primary model. Under this model, the largest predicted reductions occurred at 1,400 METs-min/week for SBP (−6.70 mmHg, 95% credible interval −9.18 to −4.29) and 1,500 METs-min/week for DBP (−4.20 mmHg, −6.52 to −1.74). The apparent attenuation at higher doses was model-dependent. At the highest observed AQE dose of 800 METs-min/week, aquatic exercise produced the largest model-estimated point reductions for both outcomes (systolic −11.63 mmHg; diastolic −7.73 mmHg); however, these estimates were based on only three AQE intervention arms and the ranking should be considered exploratory. Meta-regression found no significant moderation by age, baseline blood pressure, or intervention length. Certainty of evidence ranged from very low to low.

**Conclusion:**

Among trial populations composed predominantly of postmenopausal women, exercise was associated with a non-linear, dose-related reduction in blood pressure. The largest model-estimated reductions occurred near 1,400–1,500 METs-min/week. Aquatic exercise appeared promising, but the small number of contributing trials and the generally low certainty of evidence preclude definitive modality ranking. Evidence remains insufficient to draw stage-specific conclusions for perimenopausal women.

**Systematic review registration:**

https://www.crd.york.ac.uk/prospero/, identifier [CRD420261354599].

## 1 Introduction

Hypertension is one of the most prevalent cardiovascular risk factors worldwide, affecting an estimated 1.28 billion adults and contributing substantially to cardiovascular morbidity and mortality (1, 2). Women undergoing the menopausal transition represent a disproportionately affected population. Prior to menopause, women are relatively protected from hypertension compared with age-matched men, largely due to the vasodilatory and cardioprotective properties of estrogen (3, 4). However, the progressive decline in ovarian estrogen production during perimenopause triggers arterial stiffening, endothelial dysfunction, heightened sympathetic tone, and adverse shifts in body composition (5), driving a sharp and sustained rise in blood pressure that ultimately surpasses rates observed in men of equivalent age (6, 7). With the menopausal transition spanning a decade or more for many women, this window of accelerating cardiovascular risk demands early and targeted intervention.

Antihypertensive medication represents the primary approach for blood pressure control in this population, yet long-term pharmacological management carries risks of adverse effects including dizziness, electrolyte disturbances, and drug interactions (8), with excessive or inappropriately maintained blood pressure reduction potentially increasing rather than reducing cardiovascular risk (9). These limitations underscore the need for safe, sustainable, and well-tolerated non-pharmacological alternatives that can be integrated into long-term cardiovascular risk management strategies for women across the menopausal transition.

Exercise is a low-cost, widely accessible intervention with broad physiological benefits and is recommended in contemporary hypertension and physical activity guidance (10, 11). However, general physical activity recommendations do not constitute a blood pressure-targeted exercise prescription for peri- and postmenopausal women. Previous network meta-analyses have characterized the blood-pressure-lowering effects of exercise in broad adult populations (12) and postmenopausal women (13). Most notably, Zhang et al. (13) searched seven databases through December 2024 and synthesized 79 studies involving 3,628 postmenopausal women, identifying multi-component exercise at 900 METs-min/week as the most consistently beneficial approach. However, uncertainty remains regarding how dose estimates are affected by incomplete reporting of exercise frequency, intensity, session duration, and adherence, while evidence from perimenopausal women remains limited.

To extend this evidence, we updated the search through March 2026 and conducted a Bayesian dose-response network meta-analysis in peri- and postmenopausal women. The updated search captured eligible evidence published after the previous review’s December 2024 cutoff. To reduce dose misclassification, inclusion in the dose-response analysis required explicit reporting of exercise frequency, specific intensity, and session duration, and weekly dose was standardized as METs-min/week. When attendance was reported, prescribed dose was adjusted to approximate the dose actually completed. Our aim was therefore not to repeat the earlier analysis, but to examine how a stricter dose-extraction framework and the limited available perimenopausal evidence affected continuous dose-response patterns and modality-specific estimates. These estimates were intended as model-based anchors for interpreting the trial evidence rather than fixed clinical prescriptions.

## 2 Materials and methods

This systematic review was conducted and reported in accordance with the Preferred Reporting Items for Systematic Reviews and Meta-Analyses extension for Network Meta-Analyses (PRISMA-NMA) statement (14) and the Cochrane Handbook for Systematic Reviews of Interventions (15). The protocol was prospectively registered in PROSPERO (registration number: CRD420261354599).

### 2.1 Information sources and search strategy

Four electronic databases were systematically searched from inception to March, 2026: PubMed (NCBI), Web of Science (Core Collection), Embase (Ovid), and the Cochrane Central Register of Controlled Trials. The search strategy combined terms related to the population (e.g., “menopause,” “postmenopause,” “perimenopause,” “climacteric”), the intervention (e.g., “exercise,” “physical activity,” “aerobic training,” “resistance training,” “yoga,” “high-intensity interval training”), and the outcome (“blood pressure,” “hypertension”), restricted to randomized controlled trials. Reference lists of eligible articles and relevant previous systematic reviews were screened to identify additional records. Full search strategies for each database are provided in Electronic Supplementary Content 1. No language restriction was initially applied, but only English-language full-text articles were ultimately included.

### 2.2 Selection process

Two reviewers independently screened titles and abstracts following the predefined eligibility criteria using Zotero 7.0 for reference management and duplicate removal. Full texts of potentially eligible records were subsequently reviewed by the same two reviewers. Disagreements at any stage were resolved through discussion, with a third reviewer arbitrating when consensus could not be reached.

### 2.3 Eligibility criteria

Studies were included if they met the following PICOS criteria (16):

Population (P): Women experiencing perimenopause, menopause, or postmenopause, defined by self-reported menstrual history, hormonal criteria, or surgical menopause as reported in the primary studies.

Intervention (I): any exercise intervention. Trials in which exercise was inseparable from psychoeducational or behavioral components were eligible only when the comparator group received comparable non-exercise attention. Studies were required to report exercise type, duration, and frequency for dose–response analysis. Studies combining exercise with dietary management were excluded, as improvements may not be solely attributable to exercise. Studies examining the acute effects of exercise on blood pressure were excluded. Accordingly, only studies with an intervention length of at least 4 weeks were included to distinguish sustained exercise effects from short-term blood pressure responses.

Comparator (C): Another eligible exercise intervention or a non-exercise control group (usual care, waitlist, health education, or minimal intervention).

Outcomes (O): At least one of SBP and/or DBP, reported as change from baseline to post-intervention and analyzed as between-group mean differences in change scores when possible.

Study design (S): Parallel-arm randomized controlled trials.

Studies were excluded if they: (1) used a cross-over design; (2) combined exercise with co-interventions (e.g., caloric restriction, pharmacological therapy, dietary supplementation) in a manner that could not be isolated; (3) were conference abstracts, study protocols, systematic reviews, or gray literature; (4) did not explicitly report all three fundamental components required for calculating weekly exercise dose in METs-min/week — namely, exercise frequency, specific intensity, and session duration (17) — as these parameters could not be reliably estimated from narrative descriptions alone, a complete list of studies excluded on this basis, together with the specific missing parameter for each, is provided in Supplementary Table 2; (5) provided insufficient outcome data to pool effect estimates after contacting the corresponding authors.

### 2.4 Data extraction

A standardized extraction form was piloted on three studies prior to formal extraction. Two reviewers independently extracted the following from each eligible study: study-level characteristics (first author, year, country, funding); participant characteristics (sample size, age, menopausal stage, baseline SBP and DBP, comorbidities); intervention details (exercise modality, frequency, intensity, session duration, blood pressure measurement instrument, programme duration, adherence rate); and outcome data (pre- and post-intervention means and standard deviations for SBP and DBP, or change-from-baseline values with measures of variability). Discrepancies were resolved through discussion with a third reviewer. When data were reported only graphically, WebPlotDigitizer 4.6^1^ was used, and when necessary corresponding authors were contacted to request missing data.

### 2.5 Data conversion and dose calculation

We extracted the mean, mean difference (MD) from baseline to post-intervention, standard deviation (SD), and sample size (N) for each condition and outcome across both exercise and control groups. When SDs were not reported, they were first derived from available statistics, including confidence intervals, *p*-values, or *t*-values; if still unavailable, corresponding authors were contacted. Missing SDs for change scores were estimated using a correlation coefficient of 0.5 between baseline and post-intervention measurements, consistent with typical measurement reliability (15).

For specific data conversions, when studies reported confidence intervals, these were first converted to standard errors (SE) using the formula (18):


$$\text{SD}=\sqrt{\text{N}}\,\frac{{\text{CI}}_{\text{high}}-{\text{CI}}_{\text{low}}}{2\,t}$$

(where SD is standard deviation, N is the group sample size, CI_high_ is the upper limit of the confidence interval, CI_low_ is the lower limit of the confidence interval, and t is the t distribution with N−1 degrees of freedom the respective confidence level.)

If a study only reported standard errors (SE), they were converted to SD using the formula (18):


$$\text{SD}=\sqrt{\text{N}}\times \text{SE}$$

Furthermore, to meet the standard error requirements for dose-response analyses, SEs were calculated from SDs using the formula (18):


$$\text{SE}=\frac{\text{SD}}{\sqrt{\text{N}}}$$

For the dose-response network meta-analysis, interventions were categorized at three levels. At the first level, interventions were classified as “Exercise” or “Control.” Control conditions included no exercise, usual care, waitlist, or health education with no exercise component. At the second level, exercise interventions were further divided by type as reported in the original studies: continuous aerobic exercise (e.g., walking, running, cycling; AE), resistance training (e.g., free-weight or elastic-band strength exercises; RT), combined aerobic and resistance training performed within the same programme (AE-RT), high-intensity interval training characterized by repeated short bursts of vigorous effort interspersed with recovery intervals (HIIT), mind-body exercise or stretching (e.g., yoga, tai chi, Pilates, static stretching; MB/S), aquatic exercise performed in water (AQE), and whole-body vibration exercise performed on a vibrating platform (WBVE). Although HIIT is mechanistically a form of aerobic exercise, its intermittent structure introduces additional heterogeneity relative to continuous AE; it was therefore analyzed as a separate modality. Similarly, aquatic and whole-body vibration exercises were analyzed independently of land-based AE and RT because of their distinct physiological demands and hemodynamic profiles. At the third level, interventions were classified by total weekly exercise dose, calculated as the product of session duration, weekly frequency, exercise intensity, and exercise adherence, and expressed in METs-min/week. For duration, warm-up and cool-down periods were excluded from the total exercise time. If session duration increased progressively over the intervention period, the average total exercise time across the programme was used. For intensity, we referred to the 2024 Compendium of Physical Activities (19) and selected appropriate codes based on the exercise type and intensity descriptors (e.g., %HRmax, %HRR, %VO_2_max, %1RM, or RPE) reported in the original studies. This compendium provides MET values covering a wide range of exercise types, which were sufficient for allocating intensity in our analysis. When studies reported only qualitative intensity levels (e.g., “moderate” or “low”), we applied the ACSM absolute-intensity thresholds (light ≈ 2.0–2.9 METs, moderate ≈ 3.0–5.9 METs, vigorous ≥ 6.0 METs) and used the midpoint of each range (e.g., 4.5 METs for moderate, 8.0 METs for vigorous). For AE-RT, weekly doses of the AE and RT components were calculated separately using their respective intensity codes and then summed to yield the total weekly dose. For HIIT, all interventions were assigned code 02214 (11.0 METs) from the 2024 Adult Compendium of Physical Activities, and this value was multiplied by the reported active session duration after excluding warm-up and cool-down periods. Code 02214 represents vigorous functional interval exercise, including burpees, mountain climbers, squat jumps, and Tabata-style exercise. Because the included HIIT studies used heterogeneous exercise modes and did not consistently report phase-specific metabolic costs, work and recovery intervals could not be coded separately using a uniform approach. Consequently, applying 11.0 METs to the entire active session may have overestimated the average HIIT dose in protocols containing substantial lower-intensity recovery.

Based on the estimated METs-min/week, each intervention was assigned to the nearest predefined dose category. This approach ensured adequate network connectivity and a balanced allocation of dose points across the network. Furthermore, for studies reporting exercise session attendance, the dose categorisation was adjusted to reflect the actual exercise dose delivered, since the volume of exercise completed in interventions is often lower than the prescribed or intended dose. For example, if cycling at a moderate pace (intensity 7 METs) was prescribed three times per week for 30 min per session, the total prescribed weekly dose would be 7 × 30 × 3 = 630 METs-min/week. If the study reported a mean session attendance of 80%, the adjusted dose would be 630 × 0.8 = 504 METs-min/week, which was then rounded to the nearest predefined dose category (500 METs-min/week in this case). The specific dose allocation process is provided in Supplementary Content 4. When attendance was not reported, the prescribed dose was used; this assumption was considered when interpreting delivered-dose estimates.

### 2.6 Risk of bias and methodological quality

Risk of bias was independently assessed by two reviewers using the revised Cochrane Risk of Bias tool for randomized trials (RoB 2) (20), which evaluates five domains: the randomisation process, deviations from intended interventions, missing outcome data, measurement of the outcome, and selection of the reported result. Each domain and the overall study were rated as “low risk,” “some concerns,” or “high risk.” Disagreements were resolved through consensus or arbitration by a third reviewer.

### 2.7 Statistical analysis

#### 2.7.1 Dose-response network meta-analysis

Before conducting the dose-response network meta-analysis, key assumptions were evaluated, including network connectivity, consistency, and transitivity (21–23). Consistency was assessed by comparing the consistency model with the unrelated mean effects (UME) model using the deviance information criterion (DIC), residual deviance, and the number of effective parameters (pD). Local inconsistency was further examined through the node-splitting method, with a *p*-value < 0.05 indicating potential inconsistency. Transitivity was assessed qualitatively by examining the distribution of potential effect modifiers across treatment comparisons, including participant age, menopausal stage, baseline blood pressure, intervention duration, comorbidity profile, and control-group characteristics. Because individual participant data were unavailable, this assessment was based on reported study-level characteristics. A Bayesian model-based dose-response network meta-analysis (MBNMA) was performed using the MBNMAdose R package (version 4.4.3) (24, 25) to characterize the dose-response relationship between weekly exercise dose and changes in SBP and DBP. Vague (non-informative) prior distributions were applied to allow the data to drive the model. Eight candidate dose-response functions were evaluated: Emax, restricted cubic spline (3 knots), log-linear, non-parametric monotonically increasing, exponential, quadratic, general spline, and fractional polynomial functions. Model selection was based on the DIC, where lower values indicate a better fit. The quadratic function with random treatment effects was retained as the primary model because it yielded the lowest DIC among the eight candidate functions and was consistent with the pattern observed in the split NMA (Supplementary Table 9, with SBP presented as an illustrative example). Because the estimated turning point depends on the selected functional form, the resulting peak dose was interpreted as a model-derived estimate within the observed dose range rather than a fixed biological threshold. From the fitted quadratic dose-response function, we estimated the minimum and maximum effective doses (i.e., the range of doses producing a statistically significant reduction in BP relative to the control), the optimal dose (i.e., the dose producing the maximum reduction within the observed dose range), and the predicted mean difference [with 95% credible intervals (CrIs)] at each dose. Treatments were then ranked by their posterior probability of producing the greatest reduction at their optimal dose. Effects were considered statistically significant when the 95% CrI excluded zero. Finally, based on the established effective dose range, the exercise prescription was formulated with reference to the standardized adult physical activity intensity classification table (19).

#### 2.7.2 Subgroup and sensitivity analyses

Pre-specified exploratory subgroup analyses were conducted according to baseline SBP (>130 vs. ≤130 mmHg) and baseline DBP (>80 vs. ≤80 mmHg) (26, 27), and intervention length (>12 vs. ≤12 weeks) (28). These analyses generated separate dose-response curves for each stratum and were not formal tests of between-subgroup interaction. Three sensitivity analyses were performed to evaluate the robustness of the main findings: (1) exclusion of studies at high risk of bias; (2) exclusion of studies in perimenopausal women to restrict the analysis to postmenopausal participants; and (3) exclusion of studies in which the control group received any health education or behavioral component.

#### 2.7.3 Meta-regression

Dose-response network meta-regression was performed using the shared-effect assumption to explore potential effect modifiers of the dose-response relationship, including participant age (years), baseline blood pressure (mmHg), and intervention length (weeks). Regression coefficients are presented as posterior medians with 95% CrIs.

#### 2.7.4 Publication bias

Funnel plots, in conjunction with Egger’s asymmetry test, were used to assess small-study effects and potential publication bias (29), and *p* > 0.05 was interpreted as no statistical evidence of funnel plot asymmetry.

### 2.8 Certainty of evidence

The Confidence in Network Meta-Analysis (CINeMA) framework^2^ was applied to rate the certainty of evidence for each network estimate across six domains: within-study bias, reporting bias, indirectness, imprecision, heterogeneity, and incoherence. The overall confidence was classified as “high,” “moderate,” “low,” or “very low” (30). Detailed downgrading criteria are described in electronic Supplementary Content 9.

## 3 Results

### 3.1 Study selection

**FIGURE 1:**
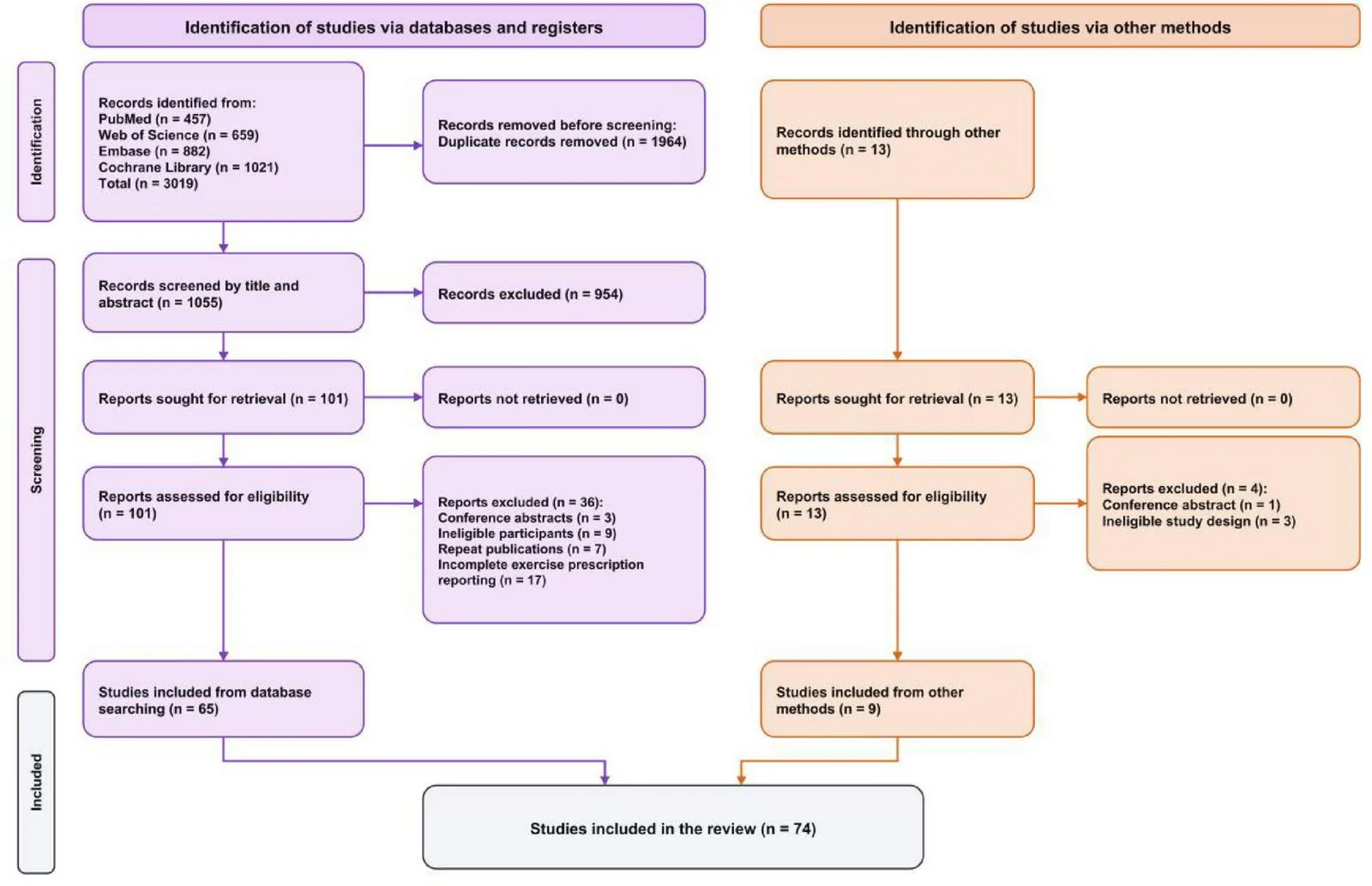
PRISMA flow diagram for study identification, screening, eligibility assessment, and inclusion.

The systematic search identified 3,019 records across four electronic databases, of which 1,964 were duplicates. After duplicate removal, 1,055 records were screened by title and abstract, and 954 records were excluded. A total of 101 full-text reports were sought and assessed for eligibility; 36 reports were excluded because they were conference abstracts (*n* = 3), involved ineligible participants (*n* = 9), were repeat publications (*n* = 7), or had incomplete reporting of exercise prescription data, including missing frequency, intensity, or duration (*n* = 17). Thus, 65 studies were identified through database searching. In addition, 13 records were identified through other methods, of which four were excluded after eligibility assessment, including conference abstract (*n* = 1) and ineligible study design (*n* = 3), yielding nine additional eligible studies. In total, 74 studies were included in the review. The PRISMA flow diagram is presented in Figure 1.

### 3.2 Characteristics of included studies

A total of 3,573 peri- and postmenopausal women were enrolled across the 74 included RCTs. Seventy of the 74 trials enrolled exclusively postmenopausal women, and the remaining four included perimenopausal participants, as detailed in Supplementary Table 4. Participants were recruited from 18 countries across Asia, the Americas, Europe, Africa, and Oceania, with the largest proportions from the United States, Brazil, and South Korea, and a mean participant age of 59.5. Regarding exercise intervention types, AE was the most common modality (45 arms), followed by RT (21 arms), AE-RT (14 arms), MB/S (11 arms), HIIT (6 arms), WBVE (4 arms), and AQE (3 arms). All passive control groups received usual care or no exercise intervention. The length of the exercise interventions ranged from 6 to 44 weeks, with a weekly frequency of 1–9 sessions per week. Detailed study characteristics are provided in Supplementary Table 4.

### 3.3 Risk of bias

Of the 74 included studies, 27 were rated as having a low risk of bias, 39 raised some concerns, and eight were categorized as high risk. Regarding the randomization process, although all RCTs involved random allocation, studies that failed to report specific methods, such as computer-generated sequences, were rated as “some concerns.” While blinding participants is inherently unfeasible in exercise interventions, bias was mitigated in trials that reported blinding of allocation personnel and other staff, whereas studies lacking any form of blinding were rated as “high risk.” Studies with potential missing outcome data were also rated as “some concerns” or “high risk.” In contrast, all studies were rated as “low risk” for outcome measurement because blood pressure was assessed using objective measurement tools. Regarding selective reporting, most studies followed predefined protocols, but those without a provided registration number were rated as “some concerns” as the risk could not be fully assessed. Detailed risk of bias results for individual studies are provided in Supplementary Figures 1, 2.

### 3.4 Hypothesis testing and model selection

The dose-level and agent-level networks were connected for both SBP and DBP, with Control serving as the principal comparator and AE contributing the largest number of intervention arms (Supplementary Figures 3–6). Consistency assessments are presented in Supplementary Contents 7.1, 7.2. Qualitative assessment of transitivity identified no clear systematic imbalance in the available study-level distributions of potential effect modifiers across the major treatment comparisons. However, because evidence for some modalities was sparse and reporting was incomplete, residual intransitivity cannot be excluded (Supplementary Content 7.3). For the consistency assumption, the consistency model and the unrelated mean effects (UME) model showed close agreement for both SBP (DIC: 786.0 vs. 787.5; residual deviance: 195.5 vs. 197.1) and DBP (DIC: 614.7 vs. 615.8; residual deviance: 182.6 vs. 183.5). Local inconsistency was assessed using node-splitting across 49 SBP and 47 DBP comparisons (96 tests in total). Most tests were non-significant, although three comparisons yielded *p* < 0.05: AE-RT_1300 vs. AE-RT_650 *p* = 0.033 and AE_300 vs. Control *p* = 0.017 for SBP, and MB/S_600 vs. Control *p* = 0.018 for DBP. Given the large number of tests and the absence of a consistent pattern, these isolated findings were interpreted cautiously and did not materially change the overall dose-response pattern. Ultimately, the quadratic function model was selected as the best-fitting approach for both SBP and DBP (DIC = 811.3 and 770.3, respectively), consistent with the dose-response pattern observed in the split NMA (Supplementary Content 7.4).

### 3.5 Dose-response relationship of overall exercise

**FIGURE 2:**
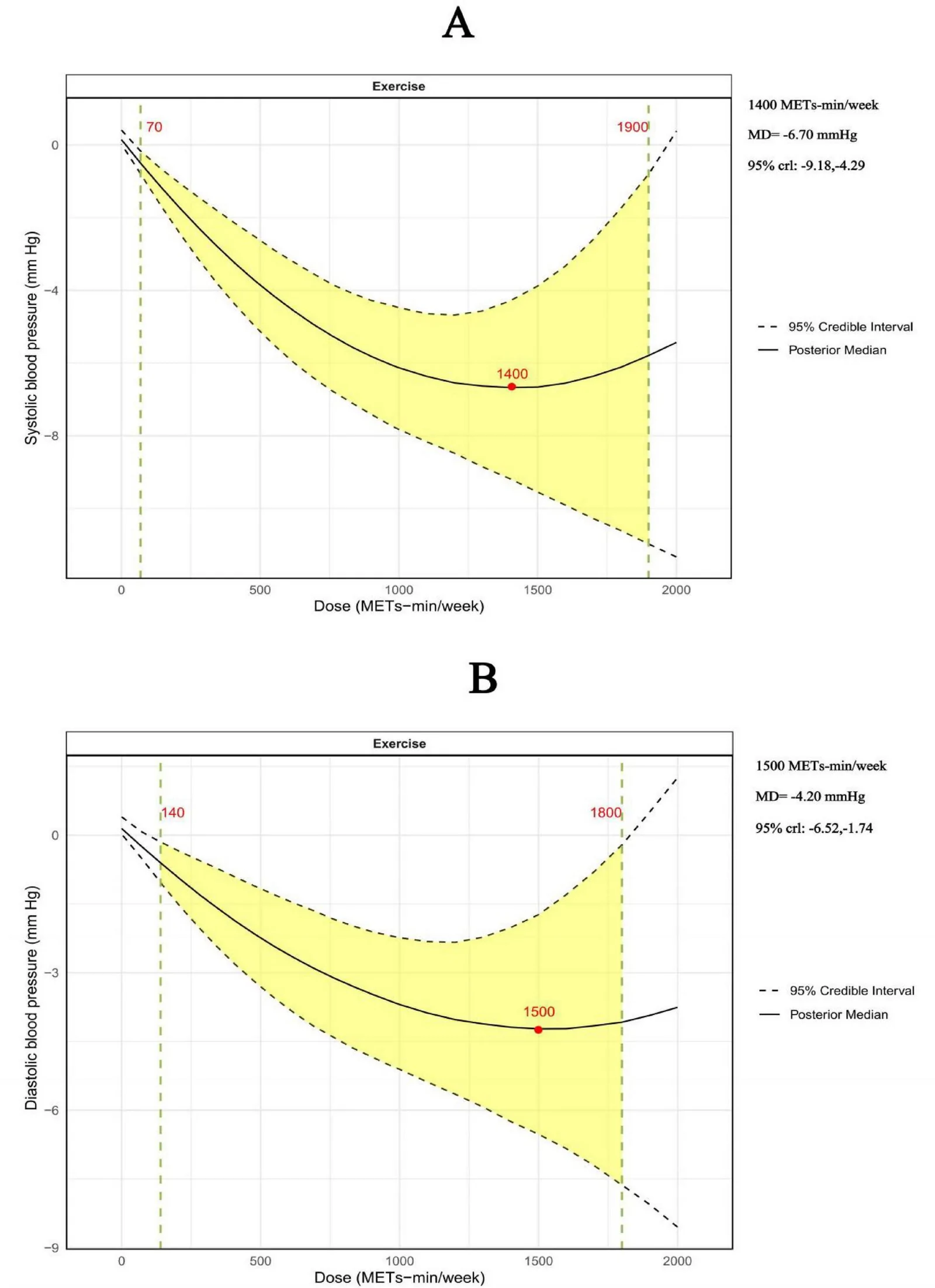
Overall model-estimated dose-response relationships between weekly exercise dose and blood pressure. **(A)** Systolic blood pressure. **(B)** Diastolic blood pressure. The solid black lines represent the posterior median, and the black dashed lines represent the 95% credible intervals. The green dashed vertical lines indicate the minimum and maximum effective doses, the yellow shaded regions indicate dose ranges in which the 95% credible interval excludes zero, and the red points indicate the model-estimated peak doses within the observed dose ranges. METs-min/week, metabolic equivalent task-minutes per week.

Table 1 systematically presents the results of the dose-response network meta-analysis. A non-linear dose-response relationship was identified between weekly exercise dose and blood pressure. For SBP, values decreased progressively with increasing exercise dose, reaching statistical significance from 70 METs-min/week and with the quadratic model estimating the largest reduction at 1,400 METs-min/week (−6.70 mmHg, 95% CrI: −9.18, −4.29). For DBP, a significant reduction was observed from 140 METs-min/week, with the quadratic model estimating the largest reduction at 1,500 METs-min/week (−4.20 mmHg, 95% CrI: −6.52, −1.74). Beyond these model-estimated peak doses, the fitted quadratic curves predicted attenuation of the blood pressure reduction; this pattern is model-dependent and should not be interpreted as a fixed biological threshold. Figure 2 presents the overall model-estimated dose-response relationships between weekly exercise dose and SBP and DBP.

**TABLE 1:** Summary of dose-response network meta-analysis results.

Category	Data point	Blood pressure	Effective dose (METs-min/week)	Optimal dose (METs-min/week)	Mean difference and 95% CrI (optimal dose)
Overall exercise	183	SBP	70–1,900	1,400	−6.70 mmHg (−9.18, −4.29)
	177	DBP	140–1,800	1,500	−4.20 mmHg (−6.52, −1.74)
Baseline SBP (>130 mmHg)	92	SBP	70–2,000	1,700	−8.96 mmHg (−13.99, −3.74)
Baseline SBP (≤130 mmHg)	73	SBP	130–1,100	920	−3.12 mmHg (−5.64, −0.68)
Baseline DBP (>80 mmHg)	87	DBP	140–2,000	1,900	−6.71 mmHg (−11.10, −2.34)
Baseline DBP (≤80 mmHg)	75	DBP	NA	NA	NA
Length (>12 weeks)	72	SBP	65–1,500	1,100	−5.95 mmHg (−9.16, −2.88)
Length (≤12 weeks)	111	SBP	70–2,000	2,000	−9.42 mmHg (−17.41, −1.51)
Length (>12 weeks)	71	DBP	390–1,100	1,100	−3.45 mmHg (−6.73, −0.21)
Length (≤12 weeks)	109	DBP	210–2,000	2,000	−7.22 mmHg (−12.56, −1.67)
Sensitivity analysis	156	SBP	70–2,000	1,500	−7.30 mmHg (−10.43, −4.23)
	154	DBP	140–1,800	1,800	−4.80 mmHg (−9.10, −0.43)
AE	78	SBP	140–2,000	1,700	−6.27 mmHg (−10.54, −2.22)
	71	DBP	210–1,600	1,600	−3.54 mmHg (−6.89, −0.23)
AE-RT	23	SBP	200–1,300	1,300	−4.99 mmHg (−10.10, −0.11)
	18	DBP	590–790	790	-3.31 mmHg (-6.65,-0.03)
AQE	6	SBP	55–800	800	−11.63 mmHg (−19.69, −3.76)
	4	DBP	110–800	800	−7.73 mmHg (−14.74, −0.69)
HIIT	10	SBP	90–1,300	1,300	−9.53 mmHg (−15.60, −3.63)
	10	DBP	180–1,300	1,300	−5.84 mmHg (−11.34, −0.39)
MB/S	16	SBP	40–600	600	−9.78 mmHg (−14.87, −4.50)
	16	DBP	NA	NA	NA
RT	40	SBP	85–1,200	1200	−6.29 mmHg (−11.99, −0.90)
	40	DBP	NA	NA	NA
WBVE	8	SBP	NA	NA	NA
	8	DBP	NA	NA	NA

NA, not applicable (no statistically significant effect observed); AE, aerobic exercise; AE-RT, combined aerobic and resistance training; RT, resistance training; MB/S, mind-body exercise or stretching; HIIT, high-intensity interval training; AQE, aquatic exercise; WBVE, whole-body vibration exercise; CG, control group.

### 3.6 Subgroup analyses

Exploratory subgroup analyses showed that, among participants with baseline SBP > 130 mmHg, the effective dose range was 70–2,000 METs-min/week, with the largest model-estimated reduction at 1,700 METs-min/week (−8.96 mmHg, 95% CrI: −13.99 to −3.74). Among participants with baseline SBP ≤ 130 mmHg, the effective dose range was 130–1,100 METs-min/week, with the largest model-estimated reduction at 920 METs-min/week (−3.12 mmHg, 95% CrI: −5.64 to −0.68). For DBP, participants with baseline DBP > 80 mmHg showed an effective dose range of 140–2,000 METs-min/week, with the largest model-estimated reduction at 1,900 METs-min/week (−6.71 mmHg, 95%CrI: −11.10 to −2.34), whereas no significant effective dose range was identified for those with baseline DBP ≤ 80 mmHg. These subgroup-specific curves were estimated separately, and no formal interaction test was performed; therefore, the apparent differences between strata should be considered exploratory.

When stratified by intervention length, exercise interventions lasting >12 weeks achieved the greatest SBP and DBP reductions at doses of 1,100 METs-min/week (SBP: −5.95 mmHg, 95% CrI: −9.16, −2.88; DBP: −3.45 mmHg, 95% CrI: −6.73, −0.21). Interventions of ≤12 weeks produced larger peak reductions at higher doses, with the greatest SBP effect observed at 2,000 METs-min/week (−9.42 mmHg, 95% CrI: −17.41, −1.51) and the greatest DBP effect also at 2,000 METs-min/week (−7.22 mmHg, 95% CrI: −12.56, −1.67), though the wider credible intervals for shorter interventions suggest greater uncertainty in these estimates. Because no formal interaction test was conducted, the apparent differences between duration strata should be considered exploratory.

### 3.7 Sensitivity analysis

Sensitivity analyses excluding studies at high risk of bias, perimenopausal participants, and studies in which the control group received a health education component did not substantially alter the primary findings. The effective dose range remained 70–2,000 METs-min/week for SBP, with an optimal dose of 1,500 METs-min/week (−7.30 mmHg, 95% CrI: −10.43, −4.23), and 140–1,800 METs-min/week for DBP, with an optimal dose of 1,800 METs-min/week (−4.80 mmHg, 95% CrI: −9.10, −0.43). Dose-response plots for all subgroup and sensitivity analyses are provided in Supplementary Contents 7.5, 7.6.

### 3.8 Dose-response relationship of various exercise modalities

**FIGURE 3:**
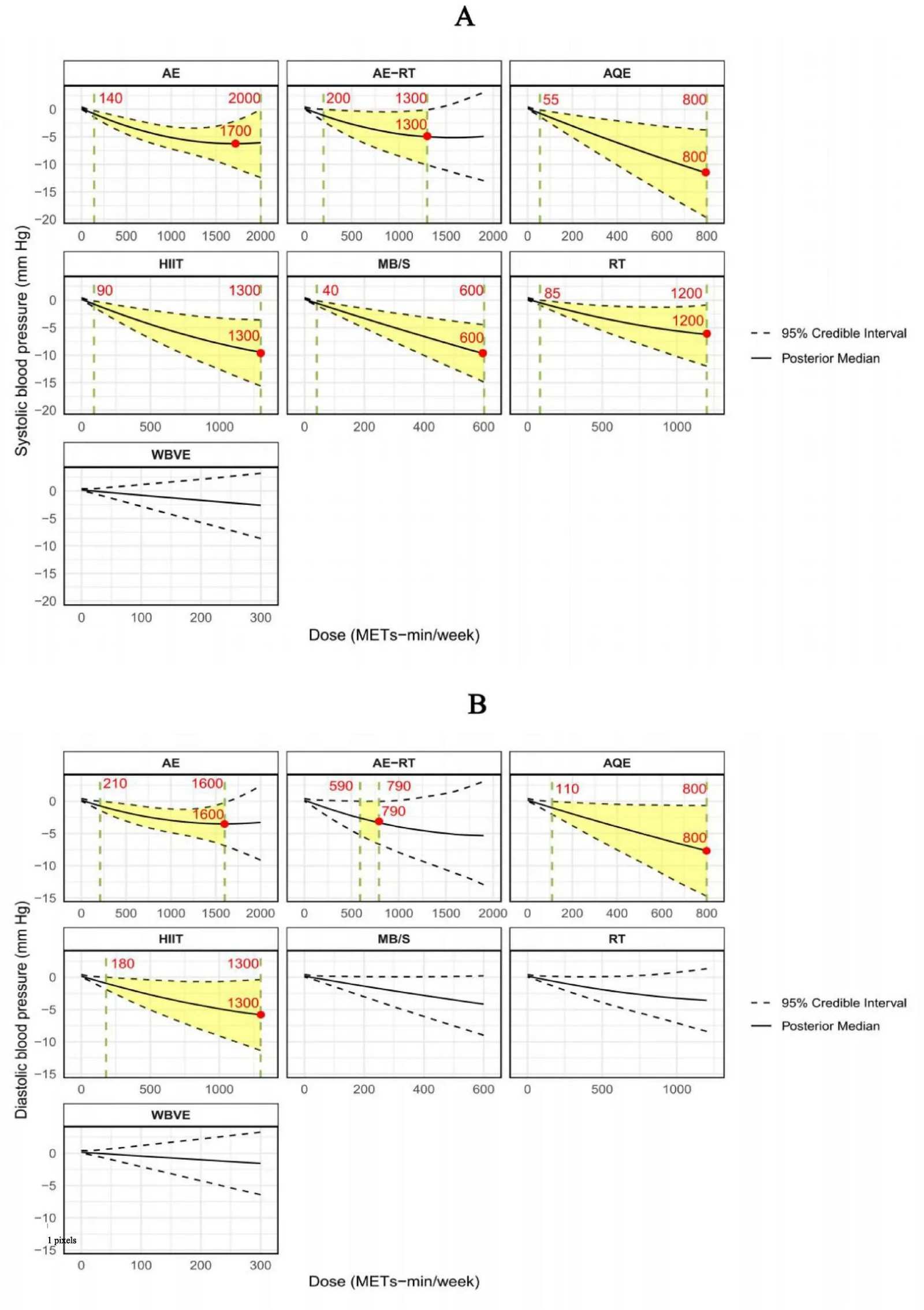
Modality-specific model-estimated dose-response relationships between weekly exercise dose and blood pressure. **(A)** Systolic blood pressure. **(B)** Diastolic blood pressure. Each panel represents a specific exercise modality. The solid black lines represent the posterior median, and the black dashed lines represent the 95% credible intervals. The green dashed vertical lines indicate the minimum and maximum effective doses, the yellow shaded regions indicate dose ranges in which the 95% credible interval excludes zero, and the red points indicate the model-estimated peak doses within the observed dose ranges. METs-min/week, metabolic equivalent task-minutes per week.

Figure 3 shows the dose-response relationships between different exercise modalities and blood pressure. For SBP (Figure 3A), AE (effective range: 140–2,000 METs-min/week), AE-RT (200–1,300 METs-min/week), AQE (55–800 METs-min/week), HIIT (90–1,300 METs-min/week), MB/S (40–600 METs-min/week), and RT (85–1,200 METs-min/week) were all significantly associated with a reduction in SBP, as indicated by the yellow-shaded regions where the 95% credible interval excluded zero. WBVE did not produce a statistically significant SBP reduction across the observed dose range. However, this finding should not be interpreted as evidence that WBVE is ineffective, but rather that the current evidence base is insufficient to characterize its dose-response relationship reliably.

At the highest observed AQE dose of 800 METs-min/week, AQE produced the largest model-estimated SBP reduction (−11.63 mmHg, 95% CrI: −19.69 to −3.76). However, this estimate was based on only three AQE intervention arms and had a wide credible interval; therefore, the ranking should be considered exploratory rather than evidence of definitive superiority (Supplementary Table 10). For DBP (Figure 3B), significant reductions were observed with AE (effective range: 210–1,600 METs-min/week), AE-RT (590–790 METs-min/week), AQE (110–800 METs-min/week), and HIIT (180–1,300 METs-min/week). No statistically significant DBP reductions were identified for MB/S, RT, or WBVE because the corresponding 95% credible intervals crossed zero. At the highest observed AQE dose of 800 METs-min/week, AQE produced the largest model-estimated DBP reduction (−7.73 mmHg, 95% CrI: −14.74 to −0.69). However, this estimate was based on only three AQE intervention arms and had a wide credible interval; therefore, the ranking should be considered exploratory rather than evidence of definitive superiority (Supplementary Table 11).

### 3.9 Meta-regression

Dose-response network meta-regressions examining the influence of age, baseline blood pressure, and intervention duration on the treatment effect are summarized in Supplementary Tables 12,13. None of the three covariates emerged as statistically significant effect modifiers for either SBP or DBP, as all 95% credible intervals for the regression coefficients crossed zero (SBP: age β = −0.06, 95% CrI: −0.46 to 0.33; baseline BP β = −0.08, 95% CrI: −0.26 to 0.12; intervention length β = −0.03, 95% CrI: −0.58 to 0.51; DBP: age β = −0.02, 95% CrI: −0.50 to 0.42; baseline BP β = −0.18, 95% CrI: −0.93 to 0.54; intervention length β = 0.02, 95% CrI: −0.56 to 0.65). Although models including baseline blood pressure or intervention duration produced lower DIC values than the unadjusted model, the corresponding regression coefficients remained uncertain and all 95% credible intervals crossed zero. The improvement in model fit should therefore not be interpreted as evidence of statistically significant effect modification.

### 3.10 Publication bias and certainty of evidence

Comparison-adjusted funnel plots for SBP and DBP are presented in Supplementary Figures 19,20. Egger’s regression test indicated no statistically significant small-study effect for either SBP or DBP, suggesting a low risk of publication bias in the included evidence base.

The certainty of evidence assessed using the CINeMA framework is summarized in Supplementary Tables 14,15. For SBP, confidence ratings ranged from very low (the majority of comparisons) to low (AQE vs. control; WBVE vs. control). For DBP, confidence ratings ranged from very low to low. Following reassessment, the AQE-versus-control comparison was downgraded from high to very low because of major concerns regarding imprecision and heterogeneity. The remaining reasons for downgrading included within-study bias and incoherence (Supplementary Table 15).

### 3.11 Illustrative translation of model-estimated dose ranges

Table 2 provides illustrative conversions of the model-estimated METs-min/week ranges into approximate weekly exercise minutes using representative intensity values from the 2024 Adult Compendium of Physical Activities. These conversions are intended to facilitate interpretation and should not be regarded as trial-validated exercise prescriptions. For example, 800 METs-min/week corresponds to approximately 140 min of moderate-intensity swimming, but this value represents the highest observed AQE dose rather than an established optimal target. Although AQE produced the largest model-estimated point reductions for both SBP and DBP, these estimates were based on only three AQE intervention arms and had wide credible intervals. Therefore, neither the AQE ranking nor the corresponding weekly minutes should be interpreted as a definitive clinical recommendation. The remaining weekly-minute estimates in Table 2 should likewise be viewed as approximate translations of model-based dose ranges and individualized according to participant characteristics and clinical context. Additional details are provided in Supplementary Table 17 and Supplementary Content 2.

**TABLE 2:** Translation of estimated dose ranges into weekly exercise minutes.

Blood pressure	Exercise modality	Energy expenditure (METs-min)*^a^	Recommended accumulation (min/week)	Optimal recommended accumulation (min/week)	Certainty of evidence (grade)
Systolic blood pressure	Cycling	7 (code: 01016)^b^	20–285	245	Very low
	Jogging	4.8 (code: 12025)^c^	30–415	355	Very low
	Walking	3.5 (code: 17160)^d^	40–570	485	Very low
	Resistance training	3.5 (code: 02054)^e^	25–345	345	Very low
	Cycling combined with resistance training	7 (code: 01016) 3.5 (code: 02054)	Cycling: 15–95; Resistance training: 30–185	Cycling: 95; Resistance training: 185	Very low
	Jogging combined with resistance training	4.8 (code: 12025) 3.5 (code: 02054)	Jogging: 20–135; Resistance training: 30–185	Jogging: 135; Resistance training: 185	Very low
	Yoga	2.3 (code: 02175)^f^	15–260	260	Very low
	Tai Chi	3.3 (code: 15670)^g^	10–180	180	Very low
	Dancing	4.8 (code: 02005)^h^	10–125	125	Very low
	Stretching	2.8 (code: 11593)^i^	15–215	215	Very low
	Swimming	5.8 (code: 18292)^j^	10–140	140	Low
	HIIT	11.0 (code: 02214)^k^	10–120	120	Very low
Diastolic blood pressure	Cycling	7 (code: 01016)^b^	30–230	230	Very low
	Jogging	4.8 (code: 12025)^c^	45-335	335	Very low
	Walking	3.5 (code: 17160)^d^	60–460	460	Very low
	Cycling combined with resistance training	7 (code: 01016) 3.5 (code: 02054)	Cycling: 40–55; Resistance training: 85–115	Cycling: 55; Resistance training: 115	Very low
	Jogging combined with resistance training	4.8 (code: 12025) 3.5 (code: 02054)	Jogging: 60–80; Resistance training: 85–115	Jogging: 80; Resistance training: 115	Very low
	Swimming	5.8 (code: 18292)^j^	20–140	140	Very low
	HIIT	11.0 (code: 02214)^k^	15–120	120	Very low

*Exercise intensity was coded based on the 2024 standardized physical activity intensity tables.

^a^METs-min, metabolic equivalents of task minutes.

^b^Cycling,: self-selected moderate pace.

^c^Jogging, in place.

^d^Walking for pleasure.

^e^Resistance (weight) training, multiple exercises, 8–15 reps at varied resistance.

*^f^*Yoga: general.

^g^Taichi: Tai chi, qi gong, general.

^h^Dance: aerobic dance, low impact, moderate effort.

^i^Stretching: sitting, teaching stretching or yoga, or light effort exercise classes.

^j^Swimming: crawl, slow speed, 30–45 yards/minute, moderate effort.

^k^HIIT: High-intensity interval exercise was coded as 11.0 METs using code 02214 from the 2024 Adult Compendium of Physical Activities. Because this code represents vigorous functional HIIT and was applied to the full active session duration, the translated dose and weekly minutes should be interpreted cautiously for protocols containing lower-intensity recovery intervals. All weekly-minute conversions are illustrative translations of model-estimated dose ranges and should not be interpreted as trial-validated exercise prescriptions or fixed clinical targets.

## 4 Discussion

### 4.1 Principal findings and implications

This study should be viewed as an extension of the recent dose-response network meta-analysis by Zhang et al. (13), rather than as the first analysis in this field. Its incremental contribution lies in an updated search through March 2026, inclusion of the limited available perimenopausal evidence, strict exclusion of trials lacking any core dose component, and attendance-adjusted dose estimation when adherence data were available. Although these criteria resulted in fewer included studies than the previous review, their purpose was to improve dose traceability rather than broaden study coverage. These design choices reduced reliance on imputation and allowed continuous dose-response estimates to be interpreted within a transparent METs-min/week framework. Nevertheless, the resulting estimates are model-derived and should not be interpreted as fixed clinical thresholds. The dose-response curves revealed a non-linear quadratic relationship between weekly exercise dose and blood pressure for both outcomes. The model-estimated peak dose for SBP reduction was 1,400 METs-min/week, equivalent to approximately 300–400 min per week of moderate-intensity walking depending on the MET value assigned, producing a mean reduction of 6.70 mmHg (95% CrI: −9.18, −4.29). For DBP, the model-estimated peak dose was 1,500 METs-min/week, yielding a mean reduction of 4.20 mmHg (95% CrI: −6.52, −1.74). Beyond these model-estimated peak doses, the fitted quadratic curves predicted attenuation of the blood pressure reduction. This apparent attenuation is conditional on the selected functional form and should not be interpreted as evidence that higher exercise doses are necessarily less effective or harmful (31).

Exploratory subgroup curves suggested larger blood pressure reductions and higher model-estimated peak doses among participants with higher baseline blood pressure. A previous dose-response network meta-analysis reported a similar descriptive pattern in postmenopausal women (13). However, the baseline blood pressure strata in the present analysis were estimated separately and were not formally compared through an interaction test. Moreover, meta-regression found no statistically significant moderation by baseline blood pressure for either SBP or DBP. The apparent subgroup differences may therefore reflect chance, limited statistical power, or other study-level characteristics. These findings should be considered hypothesis-generating and do not establish that women with higher baseline blood pressure require higher exercise doses or that dose targets should be calibrated according to baseline blood pressure.

Exploratory duration-stratified analyses produced more stable and precise estimates in programmes lasting more than 12 weeks. However, intervention duration was not a statistically significant effect modifier in the meta-regression, and no formal interaction test was performed between the duration strata. The present findings therefore do not establish a minimum programme duration of 12 weeks. Sensitivity analyses excluding studies at high risk of bias, perimenopausal participants, and control groups receiving educational components produced broadly similar overall dose-response estimates, supporting the general pattern of the primary analysis rather than a duration-specific effect.

### 4.2 Interpretation and comparison with existing literature

Previous network meta-analyses have demonstrated that exercise interventions produce clinically meaningful blood pressure reductions (32), with effect sizes varying substantially across modalities (12). A recent dose-response network meta-analysis in postmenopausal women reported that multi-component exercise at 900 METs-min/week provided the most consistent benefits for both SBP and DBP, and similarly observed greater reductions in participants with higher baseline blood pressure (13). Our effect sizes for aerobic exercise were broadly consistent with these prior estimates, though modality rankings differed. This divergence may reflect differences in eligibility criteria, modality classification, dose coding, adherence adjustment, and the evidence available at each search date; it should therefore not be attributed to any single methodological feature. The long-term blood pressure lowering effects of aerobic exercise are thought to be mediated by enhanced endothelial nitric oxide sensitivity (33), reduced sympathetic nervous system activity, and decreased systemic vascular resistance, mechanisms (34) that are particularly relevant in postmenopausal women given the well-documented impairment of endothelial vasodilatory function following estrogen withdrawal (35).

Aquatic exercise produced the largest model-estimated point reductions for both SBP and DBP at 800 METs-min/week, which was also the highest observed AQE dose. However, these estimates were derived from only three AQE intervention arms and were accompanied by wide credible intervals. The AQE ranking is therefore exploratory and should not be interpreted as evidence that aquatic exercise is definitively superior to other modalities or that 800 METs-min/week represents an established optimal prescription. Following reassessment using CINeMA, the certainty of evidence for the AQE-versus-control comparison for DBP was rated very low because of major concerns regarding imprecision and heterogeneity. This further limits confidence in the apparent AQE ranking. The haemodynamic effects of water immersion, including redistribution of peripheral venous blood centrally, increased cardiac preload and stroke volume, and stimulation of atrial natriuretic peptide release (36), may be particularly pronounced in postmenopausal women whose venous compliance and vascular reactivity are already compromised (37). The low-impact, joint-protective nature of aquatic exercise further enhances its clinical suitability for older women with musculoskeletal limitations.

Mind-body exercise and stretching at an optimal dose of 600 METs-min/week produced an SBP reduction of 9.78 mmHg, among the largest modality-specific effects observed. Prior meta-analyses of tai chi in hypertensive populations have reported SBP reductions of approximately 5–7 mmHg (38), suggesting that the larger estimates in the present study may reflect population-specific responses or differences in dose classification. The antihypertensive mechanisms of mind-body practice, encompassing parasympathetic activation, attenuation of hypothalamic-pituitary-adrenal reactivity, and reductions in sympathoadrenal tone, are particularly relevant during perimenopause and postmenopause, where hypothalamic dysregulation and heightened sympathetic activity are established drivers of blood pressure elevation (39). Notably, mind-body exercise and stretching demonstrated a broad effective dose range, making these modalities accessible to women with low exercise tolerance or functional limitations where higher-intensity exercise may be contraindicated.

High-intensity interval training was associated with model-estimated reductions in SBP and DBP, with a peak-dose estimate of 1,300 METs-min/week under the current coding approach. However, these HIIT-specific dose estimates should be interpreted cautiously because assigning 11.0 METs to the entire active session may overestimate the average exercise intensity when substantial lower-intensity recovery periods are included. A recent dose-response network meta-analysis used 8.8 METs for cycling-based HIIT, illustrating that HIIT dose estimates may vary according to the selected Compendium code and the way recovery intervals are handled (40). Therefore, the present HIIT dose estimate should not be interpreted as a fixed prescription target. Aerobic exercise and combined aerobic-resistance training both produced significant and clinically meaningful reductions across a wide dose range, supporting their continued role as practical first-line options within exercise programmes. Resistance training alone demonstrated significant SBP reductions but did not achieve significance for DBP in this population, and whole-body vibration exercise did not reach significance for either outcome, consistent with the limited and heterogeneous evidence base for these modalities. Further high-quality randomized controlled trials are needed to clarify the effects of resistance training and whole-body vibration exercise before firm recommendations can be made.

### 4.3 Limitations

Several limitations should be acknowledged. First, most comparisons were rated very low to low certainty, primarily reflecting within-study bias and substantial heterogeneity; findings should therefore be interpreted as directional evidence to inform prescription rather than definitive clinical standards. Second, only four studies included perimenopausal women, preventing reliable stage-specific subgroup analyses. Consequently, the pooled estimates primarily reflect postmenopausal populations and should not be generalized to perimenopausal women. Future trials should explicitly recruit and characterize women at different stages of the menopausal transition. Third, although the quadratic function yielded the lowest DIC among the candidate models, it may not fully capture the true biological dose-response pattern, particularly because observations were sparse at extreme doses. The estimated peak doses and apparent high-dose attenuation should therefore be interpreted as model-dependent rather than as fixed biological thresholds. Fourth, the majority of studies did not systematically report exercise-related adverse events, limiting conclusions about the safety profile of higher-dose prescriptions (41). Fifth, the subgroup-specific dose-response curves were not formally compared through interaction tests, and meta-regression found no statistically significant moderating effects of age, baseline blood pressure, or intervention duration. The apparent subgroup differences may therefore reflect limited statistical power or study-level confounding and should be considered exploratory. Individual participant data meta-analyses would be better positioned to examine these potential modifiers (42). In addition, attendance was incompletely reported across trials, so delivered-dose adjustment could be applied only to studies providing adherence data. When attendance was unreported, prescribed dose was used, which may overestimate the dose actually completed. In addition, HIIT dose was calculated by assigning 11.0 METs to the full active session duration. Because lower-intensity recovery intervals could not be weighted separately and consistently across trials, the average HIIT dose may have been overestimated. The HIIT-specific dose range and peak-dose estimate should therefore be interpreted cautiously.

## 5 Conclusion

This study systematically evaluated the dose-response relationships between exercise modalities and blood pressure in a trial evidence base composed predominantly of postmenopausal women, applying strict dose-extraction criteria to improve the accuracy of weekly exercise dose estimates. A non-linear dose-response relationship was identified for both SBP and DBP. For SBP, AE, AE-RT, AQE, HIIT, MB/S, and RT were associated with statistically significant reductions, whereas significant DBP reductions were observed for AE, AE-RT, AQE, and HIIT. AQE at the highest observed dose of 800 METs-min/week produced the largest model-estimated point reductions for both outcomes. However, because these estimates were supported by only three AQE intervention arms and wide credible intervals, the modality ranking and corresponding weekly-minute conversion should be considered exploratory rather than definitive clinical recommendations. Duration-stratified analyses produced more stable estimates in programmes lasting more than 12 weeks, but meta-regression found no statistically significant moderation by age, baseline blood pressure, or intervention duration. These subgroup patterns should therefore be considered exploratory and do not establish a minimum programme duration. However, because only four included studies involved perimenopausal women, these pooled estimates primarily apply to postmenopausal populations and should not be generalized across the entire menopausal transition. Given the low certainty of evidence for most modalities, further high-quality RCTs, particularly those explicitly recruiting perimenopausal women, are needed to confirm the model-estimated dose ranges and their clinical applicability.

## Data Availability

The original contributions presented in this study are included in this article/Supplementary material, further inquiries can be directed to the corresponding author.

## Footnotes

1
https://automeris.io/WebPlotDigitizer

2
https://cinema.ispm.unibe.ch/
