# Supplementary material for: Exercise dose and modality for blood pressure reduction in predominantly postmenopausal women: a dose-response network meta-analysis
Source: Front Med (Lausanne). 2026 Aug 12;13:1918407. doi: 10.3389/fmed.2026.1918407 (PMC13506741; doi:10.3389/fmed.2026.1918407)
Supplement: Supplementary file 1 [file Data_Sheet_1.pdf]

Supplementary content

Supplementary content 1
1.Search syntax for each database 2
1.1 Pubmed(457) 2
1.2 Web of Science(659) 2
1.3 Embase(882) 3
1.4 Cochrane(1021) 3
2. Definitions of exercise modalities 5
3.Studies excluded due to incomplete exercise prescription data 6
4. Supplementary methods 9
5. Study characteristics 10
Table S4. Study characteristics 10
6. Risk of bias 19
7. Dose-response network meta-analyses 20
7.1 Connectivity 20
7.2 Consistency 23
7.5 Subgroup analysis 38
7.6 Sensitivity analysis 44
7.7 Effectiveness ranking of different exercises and doses 47
7.7.1 Table S10. Systolic blood pressure 47
7.7.2 Table S11. Diastolic blood pressure 51
7.8 Meta-regression 56
8. Publication bias 56
8.1 Systolic blood pressure 56
8.2 Diastolic blood pressure 57
9. Grade summary of all studies 58
9.1 Within-study Bias 58
9.2 Reporting Bias 58
9.3 Indirectness 58
9.4 Imprecision 59
9.5 Heterogeneity 59
9.6 Incoherence. 59
9.7 Summarizing Judgments Across CINeMA Domains 59
Reference 69

1.Search syntax for each database

1.1 Pubmed(457)

("Menopause"[Mesh] OR "Postmenopause"[Mesh] OR "Perimenopause"[Mesh] OR menopaus*[Title/Abstract] OR postmenopaus*[Title/Abstract] OR perimenopaus*[Title/Abstract] OR climacteric*[Title/Abstract] OR menopausal women[Title/Abstract] OR postmenopausal women[Title/Abstract] OR perimenopausal women[Title/Abstract])

AND

("Exercise"[Mesh] OR "Exercise Therapy"[Mesh] OR "Motor Activity"[Mesh] OR "Sports"[Mesh] OR "Resistance Training"[Mesh] OR "Yoga"[Mesh] OR "Tai Ji"[Mesh] OR "Walking"[Mesh] OR "Physical Exertion"[Mesh] OR physical activity[Title/Abstract] OR movement[Title/Abstract] OR fitness[Title/Abstract] OR aerobic exercise[Title/Abstract]OR "high-intensity interval training"[Title/Abstract] OR "HIIT"[Title/Abstract] OR resistance training[Title/Abstract] OR strength training[Title/Abstract] OR endurance exercise[Title/Abstract] OR yoga[Title/Abstract] OR Tai Chi[Title/Abstract] OR pilates[Title/Abstract] OR dance[Title/Abstract] OR walking[Title/Abstract] OR jogging[Title/Abstract] OR stretching[Title/Abstract] OR mind-body exercise[Title/Abstract])

AND

("Blood Pressure"[Mesh] OR (Pressure, Blood[Title/Abstract]) OR (Pulse Pressure[Title/Abstract]) OR (Pressure, Pulse[Title/Abstract]) OR (Diastolic Pressure[Title/Abstract]) OR (Pressure, Diastolic[Title/Abstract]) OR (Systolic Pressure[Title/Abstract]) OR (Pressures, Systolic[Title/Abstract]) OR (Pressure, Systolic[Title/Abstract])

AND

(Randomized Controlled Trial[Title/Abstract] OR RCT[Title/Abstract] OR randomized trial[Title/Abstract] OR controlled clinical trial[Title/Abstract] OR randomized study[Title/Abstract] OR randomized controlled trial[Title/Abstract] OR randomized clinical trial[Title/Abstract] OR RCTs[Title/Abstract] OR randomized clinical studies[Title/Abstract] OR intervention trial[Title/Abstract] OR randomized intervention[Title/Abstract] OR trial[Title/Abstract] OR experimental study[Title/Abstract])

1.2 Web of Science(659)

(TS=(menopaus* OR postmenopaus* OR perimenopaus* OR climacteric* OR "menopausal women" OR "postmenopausal women" OR "perimenopausal women")

AND

(TS=(exercise OR "exercise therapy" OR "motor activity" OR sport* OR "resistance training" OR yoga OR "Tai Ji" OR "Tai Chi" OR walking OR "physical exertion" OR "physical activity" OR movement OR fitness OR "aerobic exercise" OR "high-intensity interval training" OR HIIT OR "strength training" OR "endurance exercise" OR pilates OR dance OR jogging OR stretching OR "mind-body exercise"))

AND

(TS=("blood pressure" OR "pulse pressure" OR "diastolic pressure" OR "systolic pressure" OR hypertension OR BP))

AND
(TS=("randomized controlled trial" OR RCT OR "randomized trial" OR "controlled clinical trial" OR "randomized study" OR "randomized clinical trial" OR RCTs OR "randomized clinical studies" OR "intervention trial" OR "randomized intervention" OR trial OR "experimental study" OR "random allocation" OR "double-blind" OR "single-blind"))

1.3 Embase(882)

'menopause'/exp OR 'postmenopause'/exp OR 'perimenopause'/exp OR menopaus*.mp. OR postmenopaus*.mp. OR perimenopaus*.mp. OR climacteric*.mp. OR "menopausal women".mp. OR "postmenopausal women".mp. OR "perimenopausal women".mp.
AND
'exercise'/exp OR 'exercise therapy'/exp OR 'physical activity'/exp OR 'sport'/exp OR 'resistance training'/exp OR 'yoga'/exp OR 'tai chi'/exp OR 'walking'/exp OR exercise.mp. OR "physical activity".mp. OR training.mp. OR fitness.mp. OR movement.mp. OR "aerobic exercise".mp. OR "high intensity interval training".mp. OR HIIT.mp. OR "resistance training".mp. OR "strength training".mp. OR "endurance exercise".mp. OR yoga.mp. OR "tai chi".mp. OR pilates.mp. OR dance.mp. OR walking.mp. OR jogging.mp. OR stretching.mp. OR "mind-body exercise".mp.
AND
'blood pressure'/exp OR "blood pressure".mp. OR "systolic blood pressure".mp. OR "diastolic blood pressure".mp. OR SBP.mp. OR DBP.mp. OR hypertension.mp. OR "pulse pressure".mp.
AND
'randomized controlled trial'/exp OR 'clinical trial'/exp OR randomized.mp. OR randomised.mp. OR RCT.mp. OR "randomized controlled trial".mp. OR "randomized clinical trial".mp. OR "randomized trial".mp. OR "controlled clinical trial".mp. OR "intervention trial".mp. OR "experimental study".mp.

1.4 Cochrane(1021)

#1 MeSH descriptor: [Menopause] explode all trees
#2 MeSH descriptor: [Postmenopause] explode all trees
#3 MeSH descriptor: [Perimenopause] explode all trees
#4 (menopaus* OR postmenopaus* OR perimenopaus* OR climacteric*):ti,ab,kw
#5 ("menopausal women" OR "postmenopausal women" OR "perimenopausal women"):ti,ab,kw
#6 #1 OR #2 OR #3 OR #4 OR #5
#7 MeSH descriptor: [Exercise] explode all trees
#8 MeSH descriptor: [Exercise Therapy] explode all trees
#9 MeSH descriptor: [Motor Activity] explode all trees
#10 MeSH descriptor: [Sports] explode all trees
#11 MeSH descriptor: [Resistance Training] explode all trees
#12 MeSH descriptor: [Yoga] explode all trees
#13 MeSH descriptor: [Tai Ji] explode all trees
#14 MeSH descriptor: [Walking] explode all trees

#15 MeSH descriptor: [Physical Exertion] explode all trees
#16 (physical activity OR movement OR fitness OR exercise):ti,ab,kw
#17 ("aerobic exercise" OR "high-intensity interval training" OR HIIT):ti,ab,kw
#18 ("resistance training" OR "strength training" OR "endurance exercise"):ti,ab,kw
#19 (yoga OR "tai chi" OR pilates OR dance):ti,ab,kw
#20 (walking OR jogging OR stretching OR "mind-body exercise"):ti,ab,kw
#21 #7 OR #8 OR #9 OR #10 OR #11 OR #12 OR #13 OR #14 OR #15
OR #16 OR #17 OR #18 OR #19 OR #20
#22 MeSH descriptor: [Blood Pressure] explode all trees
#23 (blood pressure OR "pulse pressure" OR "diastolic pressure"
OR "systolic pressure"):ti,ab,kw
#24 #22 OR #23
#25 MeSH descriptor: [Randomized Controlled Trial] explode all trees
#26 (randomized OR randomised OR RCT OR RCTs OR "randomized trial"
OR "randomized controlled trial" OR "randomized clinical trial"
OR "controlled clinical trial" OR "randomized study"
OR "intervention trial" OR "randomized intervention"
OR trial OR "experimental study"):ti,ab,kw
#27 #25 OR #26
#28 #6 AND #21 AND #24 AND #27

2. Definitions of exercise modalities

Table S1. Definitions of exercise modalities

Abbreviation	Full name	Definitions
AE	Aerobic Exercise	Aerobic exercise is performed by repeating sequences of light-to-moderate intensity activities for extended periods of time. e.g., walking, bicycle, and treadmill training etc.
AQE	Aquatic Exercise	Gait training, balance training, resistance training, or aerobic training performed in deep or shallow water.
CG	Control group	Non-exercise intervention, usual care, or health education
MB	Mind-body exercises	A form of exercise that combines body movement, mental focus, and controlled breathing to improve strength, balance, flexibility, and overall health. Examples of mind -body exercises are yoga, tai chi, and qigong.
Stretching (S)	-	Stretching involves slowly stretching a muscle/tendon group and holding the position for a period of time (10−30 s). Static stretches can be either active, as is common in many forms of yoga, or passive.
RT	Resistance Training	Exercise training designed to improve the strength, power, endurance and size of skeletal muscles.
AE-RT	Aerobic combined resistance training	During the same training phase or cycle, a training regimen combines aerobic exercises designed to enhance cardiopulmonary function with resistance training aimed at improving muscle strength and endurance.
HIIT	High-intensity intermittent	It typically consists of alternating high-intensity exercise lasting 15 seconds to 4 minutes with passive or low-intensity recovery periods. The heart rate during the high-intensity phase approaches 80% to 95% of the maximum heart rate.
WBVE	Whole-Body Vibration Exercise	A form of exercise using a vibrating platform to stimulate involuntary muscle contractions for improved strength and balance.

3.Studies excluded due to incomplete exercise prescription data

Table S2: Studies excluded due to incomplete exercise prescription data (n = 17).

study	Interventions (n)	Menopausal stage	Frequency (times/week)	Duration(min)	Exercise intensity	Exercise-related adverse events	age	Country	Reason
Arazi et al.2018[1]	AQE(10)	Postmenopause	2	30-40	Medium-High	NO	58(3.55)	Iran	No extractable SBP
	CG(10)		0	0			59.1(3.07)		
Arca et al.2014[2]	AQE(19)	Postmenopause	3	50	NR	NR	64(7.0)	Brazil	Poor intensity measurement
	AE(19)		3	50					
	CG(14)		0	0					
Asikainen et al.2003[3]	AE(46)	Postmenopause	5	NR	High	NO	58(4.4)	Finland	No explicit session duration
	AE(43)		5				58(4.2)		
	CG(45)		0				57(4.2)		
	AE(21)		5		Low-Medium		57(3.8)		
	AE(21)		5				55(3.7)		
	AE(18)		5				54(3.5)		
	AE(21)		5				55(4.2)		
	CG(40)		0				56(3.8)		
	CG(12)		0	0			72(7.07)		
Cardoso et al.2011[4]	AE(12)	Postmenopause	3	20-60	Medium-High	One participant excluded due to	50(1)	Brazil	No extractable SBP
	CG(12)		0	0			50(1)		

						exercise-induced asthma			
Elliott et al.2002[5]	RT(8)<br>CG(7)	Postmenopause	3<br>0	NR<br>0	Low	NR	58(4)<br>53(3)	UK	No explicit session duration
Figueroa et al.2011[6]	AE-RT(12)<br>CG(12)	Postmenopause	3<br>0	40<br>0	Medium	NR	54(2)<br>54(1)	USA	No extractable SBP
Figueroa et al.2015[7]	WBVE(12)<br>WBVE(12)<br>CG(12)	Postmenopause	3<br>3<br>0	NR<br>NR<br>0	NR	NR	56(1)<br>58(1)<br>58(1)	USA	No explicit session duration
Hartman et al.2025[8]	AE(136)<br>AE(136)<br>CG(135)	Postmenopause	NR<br>NR<br>0	NR<br>NR<br>0	NR	NO	68.4(7.66)<br>67.3(6.55)<br>67.8(7.23)	USA	No frequency,intensity, or session duration
Innes et al.2012[9]	MB/S(10)<br>CG(10)	Postmenopause	NR<br>2	90<br>90	NR	NO	58.4(6.32)<br>58.9(9.11)	USA	Home practice frequency missing
Latosik et al.2014[10]	AE(12)<br>CG(12)	Postmenopause	NR<br>0	45<br>0	Low-Medium	NR	NR<br>NR	Namibia	No weekly frequency
Lima et al.2012[11]	AE(10)<br>CG(12)	Postmenopause	5<br>0	20-60<br>0	Medium	NR	54.9(1.7)<br>52.(1.6)	Brazil	No extractable SBP
Myslivecek et al.2002[12]	AE(8)<br>CG(8)<br>CG(10)	Postmenopause	≥3<br>0<br>0	NR<br>0<br>0	Medium	NR	52.1(1.5)<br>51.4(1.6)<br>51.4(3.06)	Canada	No explicit session duration

Son et al.2017[13]	AE-RT(10)	Postmenopause	3	60	Low-Medium	NR	76(5)	South Korea	Conflicting weekly frequency (3 vs5)
	CG(10)		0	0			74.7(2)		
Stachenfeld et al.1998[14]	AE(9)	Postmenopause	3.5	30-50	Low-Medium	NR	71(2)	USA	No extractable SBP
	MB/S (8)		3.5	60	NR		73(3)		
Tudor-Locke et al.2020[15]	AE(49)	Postmenopause	7	NR	NR	NR	62.6(6.5)	USA	No explicit session duration,extractable SBP
	AE(47)		7	NR	Medium		61.7(6.2)		
	CG(19)		0	0	NR		58.4(5.8)		
Zhang et al.2019[16]	HIIT(15)	Postmenopause	3	20	High	NR	53.2(3.5)	Australia	No explicit session duration
	CG(15)		0	0			53.0(3.1)		
Zhou et al.2022[17]	AQE(12)	Postmenopause	NR	NR	Medium-High	NR	62.3(5.02)	China	No explicit session duration
	AQE(61)		NR	NR			62.2(6.17)		

4. Supplementary methods

- **Agent:** The agent level.
- **METs_min:** The energy expenditure per min in the study.
- **Min_session:** The duration of a single exercise intervention.
- **Frequency:** The number of times participants engaged in physical activity (times/week).
- **Exact_dose:** The exact estimated METs accumulated per week by participants (METs-min/week).
- **Dose:** The dose group by approximation (METs-min/week).
- **CG:** Control group

Table S3. Dose categorization table

Study ID	Agent	METs_min	Min_session	Frequency	Prescribed dose	Average adherence rate (%)	Exact_dose	Dose
1	AE	7	30	3	630	80	504	500
1	CG	0	0	0	0	0	0	0
2	RT	3.5	20	3	210	100	210	250
2	CG	0	0	0	0	0	0	0
3	AE-RT	AE: 5.5	20	3	330+210	92	496.8	500
3		RT: 3.5	20	3				
3	CG	0	0	0	0	0	0	0
4	HIIT	7	20	3	420	91	382.2	400
4	CG	0	0	0	0	0	0	0
5	MB/S(Yoga)	3.3	30	3	297	90	267.3	250
5	CG	0	0	0	0	0	0	0
6	AQE	5.8	30	3	522	90	459.8	450
6	CG	0	0	0	0	0	0	0

5. Study characteristics

Table S4. Study characteristics

study	Interventions (n)	Menopausal stage	Frequency (times/week)	Duration (min)	Exercise intensity	Blood Pressure monitor	Exercise-related adverse events	age	Country
Akwa et al.2017[18]	AE(8)	Postmenopause	3	24	Medium	Omron BP Monitor M10-IT)	NR	63.5(9.85)	Ghana
	CG(10)		3	0				59.5(7.78)	
Ammar et al.2015[19]	AE(15)	Postmenopause	3	30	Medium	Mercury sphygmomanometer (Model TXJ-10, Japan)	NR	53.24(1.94)	Egypt
	CG(15)		0	0				53.24(1.82)	
Anuradha et al.2025[20]	MB/S(30)	Postmenopause	5	36	Low-Medium	BP Apparatus (Master Care, India)+Stethoscope (Micro Tone, India)	NR	50.63(6.24)	India
	CG(30)		0	0				50.47(8.06)	
Azadpour et al.2017[21]	AE(12)	Postmenopause	3	35	Medium	HBP-1300,Omron Healthcare, Inc, Kyoto, Japan	NR	57.58(4.29)	Turkey
	CG(12)		0	0				56.58(4.17)	
Batista et al.2022[22]	MB/S(24)	Postmenopause	3	50	Medium	OMRON HEM-7113	NR	58(5)	Brazil
	MB/S(23)		3	50				58(4)	
Binder et al.1996[23]	AE(23)	Postmenopause	3.3–3.7	45	Medium-High	NR	NR	65(4)	USA
	CG(11)		0	45				67(4)	
Boutcher et al.2019[24]	HIIT(20)	Postmenopause	3	30	High	OMRON, Bannockburn, IL	NR	54.1(3.6)	Australia
	CG(20)		0	0				53.3(3.4)	
Casey et al.2007[25]	RT(13)	Postmenopause	2	30	Low-Medium	Omron, Inc	NR	58.7(4.5)	USA

	AE(10)		2	30-40	Medium			59.7(6.5)	
Colado et al.2009[26]	AQE(15)	Postmenopause	3	45	Medium	NR	NR	54.7(2.0)	Spain
	RT(21)		3	45				54.0(2.8)	
	CG(10)		0	0				52.9(1.9)	
Conceição et al.2013[27]	RT(10)	Postmenopause	3	45-50	Medium-High	Mercury sphygmomanometer +stethoscope	NR	53.4(3.95)	Brazil
	CG(10)		0	0				53.0(5.7)	
Corrick et al.2013[28]	AE-RT(27)	Postmenopause	2	50	Medium-High	Omron HEM-780	NR	65.6(0.7)	USA
	AE-RT(30)		4	50				63.7(0.5)	
	AE-RT(22)		6	50				64.8(0.7)	
Dalleck et al.2009[29]	AE(8)	Postmenopause	5	30	Medium	NR	NR	59.9(3.1)	USA
	AE(8)		5	45				55.4(3.2)	
	CG(10)		5	0				57.4(4.6)	
El-Refaye et al.2019[30]	RT(20)	Postmenopause	3	40	Medium	Mercury column sphygmomanometer	NR	55.0(3.24)	Egypt
	AE(20)		3	40				53.9(3.12)	
	CG(20)		0	0				54.9(3.22)	
Fairey et al.2005[31]	AE(24)	Postmenopause	3	25	Medium-High	Random zero sphygmomanometer(mercury sphygmomanometer)	Lymphedema (3), gynecologic complication (1), influenza (1)	59(5)	Canada
	CG(28)		3	0				58(6)	
Ferreira et al.2024[32]	AE(51)	Postmenopause	3	50	Medium - High	BIC AP0335 - Innova, Brazil	NR	NR	Brazil
	CG(14)		0	0				NR	
Figueroa et al.2014[33]	WBVE(15)	Postmenopause	3	35	Medium	NR	NR	56(3)	USA
	CG(13)		0	0				56(3)	
Garnier et al.2024[34]	AE(196)	Postmenopause	3	45	Medium	NAIS, Blood Pressure Unit	NR	60(5)	France
	CG(52)		0	0				60(5)	

Gelecek et al.2012[35]	RT(24)<br>CG(21)	Postmenopause	3<br>0	55<br>0	Medium	Mercury sphygmomanometer	NR	54.3(5.3)<br>51.8(3.65)	Turkey
Gerage et al.2013[36]	RT(15)<br>MB/S(14)	Postmenopause	3<br>2	40-50<br>25-40	Medium<br>Low	Mercury sphygmomanometer (Missouri) +stethoscope (Littmann Classic II)	NR	65.5(5)<br>66.2(4.1)	Brazil
Ghadamyari et al.2025[37]	AE(10)<br>RT(10)<br>CG(10)	Postmenopause	3<br>3<br>0	60<br>60<br>0	High	NR	NR	55.1(2.85)<br>58.1(3.18)<br>55.75(3.61)	Iran
Gholamrezai et al.2014[38]	AE(10)<br>CG(10)	Postmenopause	3<br>0	50<br>0	Medium-High	Standard sphygmomanometry	NR	52.5(3.83)<br>51.4(3.06)	Iran
Henríquez et al.2017[39]	AE(18)<br>RT(16)	Postmenopause	3<br>3	40<br>40	Medium<br>Low	NR	NR	58(40)<br>55(4)	Chile
Jaime et al.2019[40]	WBVE(13)<br>RT(12)<br>CG(8)	Postmenopause	3<br>3<br>0	30<br>30<br>0	Medium<br>Low	Omron HEM-907XL	NR	65(4)<br>65(4)<br>65(4)	USA
Jeon et al. 2018[41]	AE-RT(8)<br>CG(8)	Postmenopause	3<br>0	32<br>0	Medium-High	Easy X 800R; Jawan Medical, South Korea	NR	59(2.83)<br>58(5.66)	South Korea
Kamal et al.2024[42]	WBVE(15)<br>CG(15)	Postmenopause	3<br>0	30<br>0	Low–Moderate	Mercury sphygmomanometer	NR	57.3(1.67)<br>56.9(1.83)	Egypt
Kazemi et al.2023[43]	HIIT(15)<br>RT(15)<br>CG(15)	Postmenopause	3<br>3<br>0	33<br>45<br>0	High	OMRON digital monitor	NR	NR<br>NR<br>NR	Iran

Kim et al.2012[44]	MB/S(15)	Postmenopause	3	60	Medium - High	NR	NR	53.5(2.4)	South Korea
	CG(15)		0	0				53.5(2.4)	
Lee et al.2012[45]	MB/S(8)	Postmenopause	3	60	Low-Medium	NR	NR	54.8(2.76)	South Korea
	CG(8)		0	0				54.3(2.91)	
Lee et al.2019[46]	AE(10)	Postmenopause	3	60	Low - Medium	NR	NR	70(4)	South Korea
	CG(10)		0	0				70(4)	
Lindheim et al.1994[47]	AE(25)	Postmenopause	3	30	Medium	NR	NR	48.8(1.1)	USA
	CG(20)		0	0				50.8(1.2)	
	AE(28)		3	30				48.5(0.9)	
	CG(28)		0	0				49.4(1.0)	
Lopez et al.2024[48]	RT(35)	Postmenopause	2	60	Medium	OMRON M3, OMRON Corporation, Kyoto, Japan	NR	69.17(5.71)	Spain
	CG(11)		0	0				67.90(8.60)	
	RT(35)		2	60				70.80(5.86)	
	CG(12)		0	0				72.00(7.07)	
Lyall et al.2022[49]	AE(13)	Postmenopause	3	30	Medium	NR	NR	55(3)	UK
	HIIT(14)		3	21	High			57(4)	
Maeda et al.2004[50]	AE(10)	Postmenopause	5	30	Low-Medium	NR	NR	63(4)	Japan
	CG(5)		0	0				64(4)	
Matsubara et al.2014[51]	AE(11)	Postmenopause	3	45	Medium - High	Colin Medical Technology, Komaki, Japan]	NR	62(2)	Japan
	CG(8)		0	0				62(3)	
McGavock et al.2004[52]	AE-RT(11)	Postmenopause	3	70	Low-Medium	NR	NR	59(7)	Canada
	CG(7)		0	0				59(7)	
Miura et al.2008[53]	RT(29)	Postmenopause	1	60	Low-Medium	BP-203RPEII, Colin, MT, Japan	NR	69.0(6.5)	Japan
	RT(25)		2	60				69.5(7.0)	

	CG(23)		0	0				68.9(7.5)	
	CG(10)		0	0				67.4(1.1)	
Moreau 1999[54]	AE(12)	Postmenopause	7	30-45	Medium	NR	NR	NR	USA
	AE(12)		7	30-45				NR	
	CG(13)		0	0				NR	
Moreau et al.2001[55]	AE(15)	Postmenopause	7	30	Medium	NR	NR	53(2)	USA
	CG(9)		0	0				55(1)	
Nemotoet al.2007[56]	HIIT(31)	Postmenopause	≥4	51±1	High	NR	NR	64(6)	Japan
	AE(43)		≥4	61±1	Medium			62(5)	
	CG(37)		0	0				62(6)	
Nishida et al.2015[57]	AE(31)	Postmenopause	9	15	Medium	BP-203RV, Nihon Colin, Tokyo, Japan	NR	70.4(5.8)	Japan
	CG(31)		0	0				69.7(6.6)	
Nunes et al.2022[58]	HIIT(24)	Postmenopause	3	20	High	OMRON, model HEM-710INT	NR	61.5(7.0)	Brazil
	AE-RT(25)		3	37.5	Medium			62.6(8.5)	
Nuri et al.2012[59]	AE-RT(14)	Perimenopause	4	95	Medium - High	ALPK2	NR	58.27(6.31)	Iran
	CG(15)		0	0				58.27(6.31)	
Oneda et al .2014[60]	AE(12)	Postmenopause	3	20-50	Medium - High	Dixtal 2710 (oscillometric)	NR	50(3)	Brazil
	CG(11)		3	0				50(3)	
	AE(8)		3	20-50				52(3)	
	CG(14)		3	0				51(4)	
Paolillo et al.2013[61]	AE(10)	Postmenopause	2	45	High	Aneroid sphygmomanometer (auscultatory)	NR	55(2)	Brazil
	CG(10)		2	0				55(2)	
Pospieszna et al.2017[62]	AE(20)	Postmenopause	3	50	Medium	NR	NR	62(3.79)	Poland
	CG(19)		3	0				62(1.10)	

Rahnama et al.2010[63]	AE-RT(14)	Perimenopause	2	50	Medium - High	ALPK2	NR	NR	Iran
	CG(15)		2	0				NR	
Ready et al.1996[64]	AE(18)	Postmenopause	3	60	Medium	NR	6 injuries (pre-existing conditions)	61.3(5.8)	Canada
	AE(17)		5	60				61.3(5.9)	
	CG(18)		0	0				61.3(5.10)	
Reis et al.2012[65]	RT(27)	Postmenopause	2	45	Medium - High	NR	NR	52.91(4.02)	Brazil
	CG(31)		0	0				53.86(5.11)	
Rezende Barbosa et al.2019[66]	AE-RT(19)	Postmenopause	3	69.3	Medium	Aneroid sphygmomanometer (R appaport Premium stethoscope, Wenzhou Instruments, Wenzhou, China)	NR	60(4.5)	Brazil
	CG(20)		0	0				58.45(4.85)	
Santa-Clara et al.2003[67]	AE(17)	Perimenopause	3—4	53	Medium - High	Standard sphygmomanometry	NR	59(6)	USA
	CG(16)		0	0				57(7)	
	AE(15)		3—4	53				58(5)	
	CG(12)		0	0				55(5)	
Sardeli et al.2022[68]	AE-RT(12)	Postmenopause	walk 3 rt 2	65	Medium	Finometer Pro® (Finapres Medical System, Amsterdam, The Netherlands)	NR	64.5(3.9)	Brazil
	CG(13)		0	0				65.6(5.6)	
Sattar et al.2012[69]	AQE(14)	Postmenopause	3	53	Medium	NR	NR	55.2(3.7)	Iran
	CG(10)		0	0				54.4(4.6)	
Sénéchal et al.2012[70]	RT(10)	Postmenopause	3	40	Medium - High	Dinamap automatic machine (Critikon; Johnson & Johnson,	NR	62.6(4.1)	Canada
	CG(10)		0	0				62.6(4.2)	
	RT(9)		3	40				62.6(4.3)	

	CG(9)		0	0		Tampa, FL)		62.6(4.4)	
Seo et al.2023[71]	RT(15)<br>CG(13)	Postmenopause	3<br>0	80<br>0	Medium - High	HBP-9020 OMRON Colin	NR	NR	South Korea
Seo et al.2010[72]	AE-RT(8)<br>CG(7)	Postmenopause	3<br>0	60<br>0	Medium - High	NR	NR	54(3.6)<br>58(4.2)	South Korea
Serrano-Guzmánet al.2016[73]	MB/S(27)<br>CG(25)	Postmenopause	3<br>0	40<br>0	Low-Medium	Standard mercury sphygmomanometer	NR	69.07(4.41)<br>69.48(3.22)	Spain
Shabani et al.2018[74]	AE-RT(12)<br>CG(12)	Postmenopause	3<br>0	90<br>0	Medium	Rossmax validated automated oscillometric cuff	NR	56.90(4.93)<br>54.83(4.72)	Iran
Shakoor et al.2016[75]	AE(12)<br>CG(12)	Postmenopause	3<br>0	30<br>0	Medium	Standard sphygmomanometer	NR	67.85(5.67)<br>67.85(5.67)	Iran
Shaw et al.2016[76]	RT(19)<br>CG(18)	Postmenopause	2<br>0	40<br>0	Medium	Sphygmomanometer +stethoscope	NR	60.44(5.34)<br>57.74(2.83)	South Africa
Sobrinho et al.2023[77]	MB/S(23)<br>AE-RT(20)<br>CG(21)<br>MB/S(28)<br>AE-RT(23)<br>CG(26)	Postmenopause	2<br>2<br>0<br>2<br>2<br>0	30<br>90<br>0<br>30<br>90<br>0	Medium-High	OMRON HEM-7113	NR	64.4(2.3)<br>65.3(5.0)<br>66.1(4.9)<br>65.1(4.0)<br>65.3(3.9)<br>66.8(5.4)	Brazil
Son et al.2020[78]	RT(10)<br>CG(10)	Postmenopause	3<br>0	40<br>0	Medium	Omron BP-200	NR	67.7(1)<br>67.4(1.1)	South Korea
Son et al. 2021[79]	RT(18)<br>CG(17)	Postmenopause	3<br>0	40<br>0	Medium	Omron BP-200	NR	68.2(1.6)<br>68.2(1.4)	South Korea
Staffileno et	AE(9)	Postmenopause	5	30	Medium	Mercury	NR	57.1(8.7)	USA

al.2001[80]	CG(9)		0	0		sphygmomanometer		62.3(8.7)	
Sugawara et al.2012[81]	AE(11)<br>CG(11)<br>AE(12)<br>CG(11)	Perimenopause	4<br>0<br>4<br>0	35<br>0<br>35<br>0	Medium	Form PWV/ABI +Jentow(Colin Medical Technology， Japan)	NR	59(2)<br>59(2)<br>60(1)<br>61(2)	Japan
Swift et al.2012[82]	AE(140)<br>AE(82)<br>AE(93)<br>CG(89)	Postmenopause	3—4<br>3—4<br>3—4<br>0	45<br>45<br>45<br>0	Medium	Colin STBP-780	NR	57.2(6.5)<br>56.6(6.3)<br>56.8(6.4)<br>57.0(5.8)	USA
Taha et al.2016[83]	HIIT(23)<br>CG(23)	Postmenopaus	3<br>0	40<br>0	High	Aneroid sphygmomanometer	NR	48.17(2.20)<br>47.78(2.59)	Egypt
Tanahashiet al.2014[84]	AE(20)<br>CG(10)	Postmenopause	4.7<br>0	48<br>0	Medium-High	Form PWV/ABI (Colin Medical Technology, Japan)	NR	62(6)<br>61(7)	Japan
Khalid et al.2013[85]	AE(12)<br>CG(13)	Postmenopause	3<br>0	40<br>0	Medium	NR	3 dropouts: back pain, no blood sample, chest infection	52.9(2.6)<br>52.7(2.2)	Egypt
Wang et al.2024[86]	MB/S(20)<br>CG(20)	Postmenopause	4<br>0	40<br>0	Medium	UNEX EF, Omron BP-203R PE2	NR	54.2(4.60)<br>53.7(4.26)	China
Wong et al.2014[87]	MB/S(14)<br>CG(14)	Postmenopause	3<br>0	50<br>0	Low	OmronVP-2000, Finometer, Millar SPT301B	NR	57(1)<br>56(1)	USA
Wong et al.2016[88]	WBVE(13)<br>CG(12)	Postmenopause	3<br>0	11-60<br>0	Low-Medium	Omron VP-2000	NR	59(1)<br>59(1)	USA
Wong et	AE(20)	Postmenopause	4	30-40	Medium	Colin VP-1000	NR	59(1)	South

al.2018[89]	CG(21)		0	0				59(1)	Korea
Wong et al.2019[90]	AQE(52)	Postmenopause	3--4	32.5-37.5	Medium	Omron HEM-705CP	NR	74(4)	South Korea
	CG(48)		0	0				73(4)	
Yoshizawa et al.2009[91]	AE(11)	Postmenopause	4	35	Medium	Form PWV/ABI (Colin Medical Technology, Komaki, Japan)	NR	57(1)	Japan
	CG(11)		0	0				59(1)	
	AE(12)		4	35				56(1)	
	CG(11)		0	0				57(1)	

Note: For HIIT interventions, weekly dose was calculated using 11.0 METs for the reported active session duration after excluding warm-up and cool-down. Work and recovery intervals were not weighted separately because phase-specific recovery intensity was not consistently available across studies.

6. Risk of bias

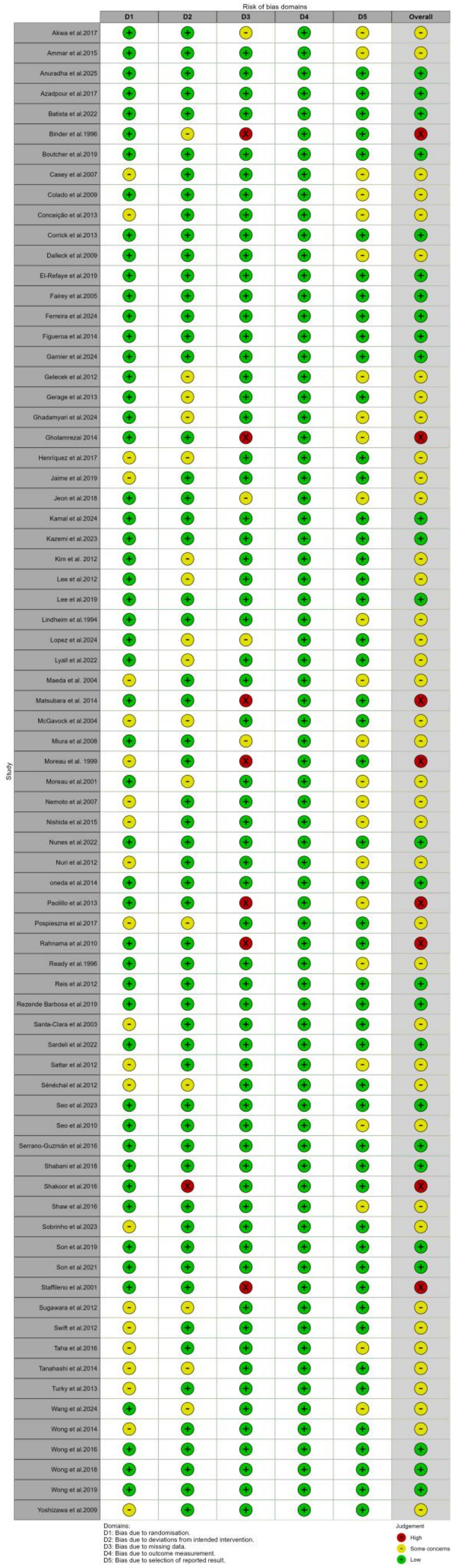

Figure S1. The detail of risk of bias.

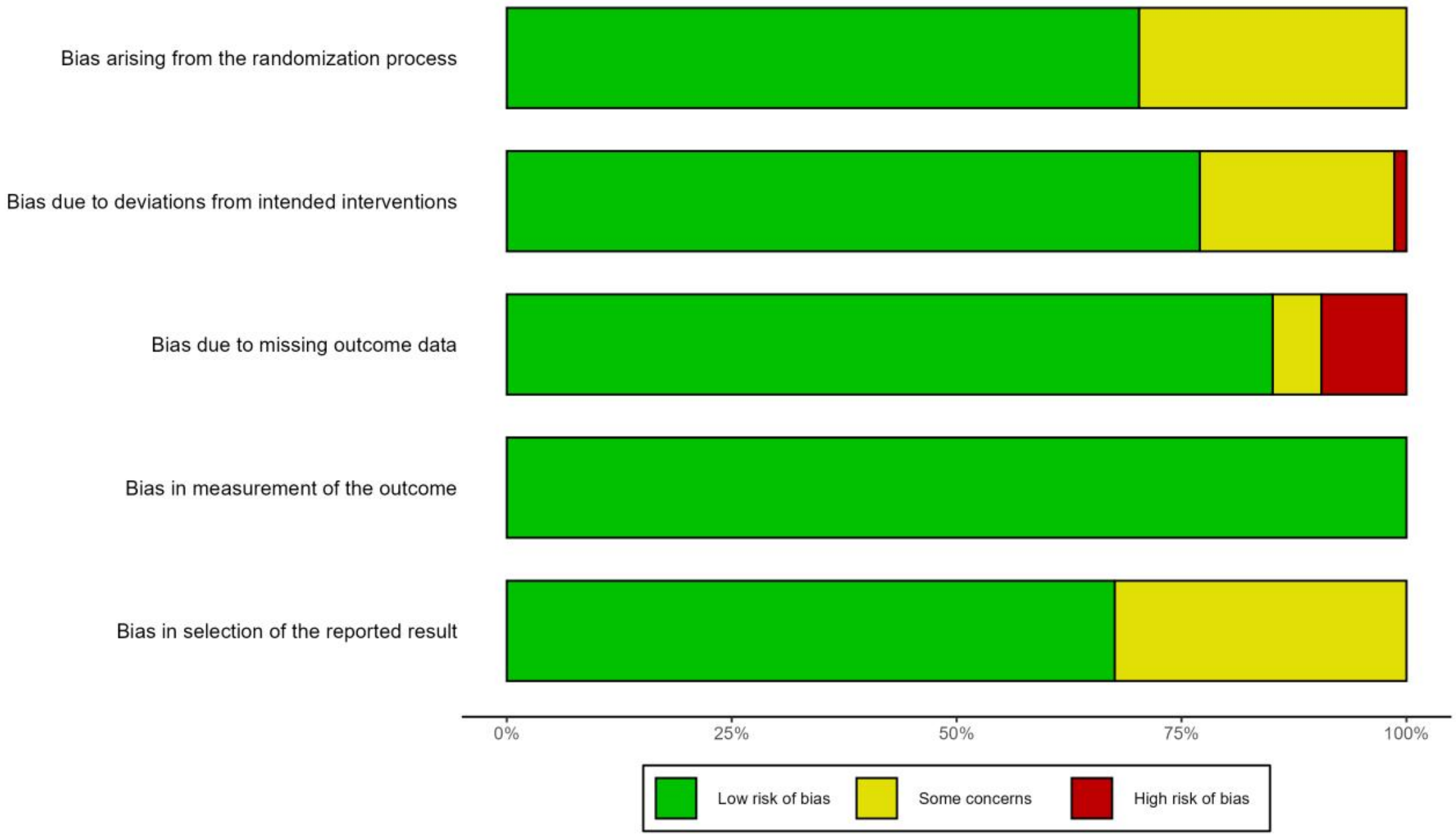

Figure S2. The summary of risk of bias.

7. Dose-response network meta-analyses

7.1 Connectivity

Connectivity is a fundamental assumption in network meta-analysis (NMA). Insufficient connectivity, often resulting from limited direct comparisons, can reduce statistical power and bias results. In our study, connectivity was assessed across all dose and agent levels for each outcome (Figures S3-S6), with no evidence of network disconnection observed.

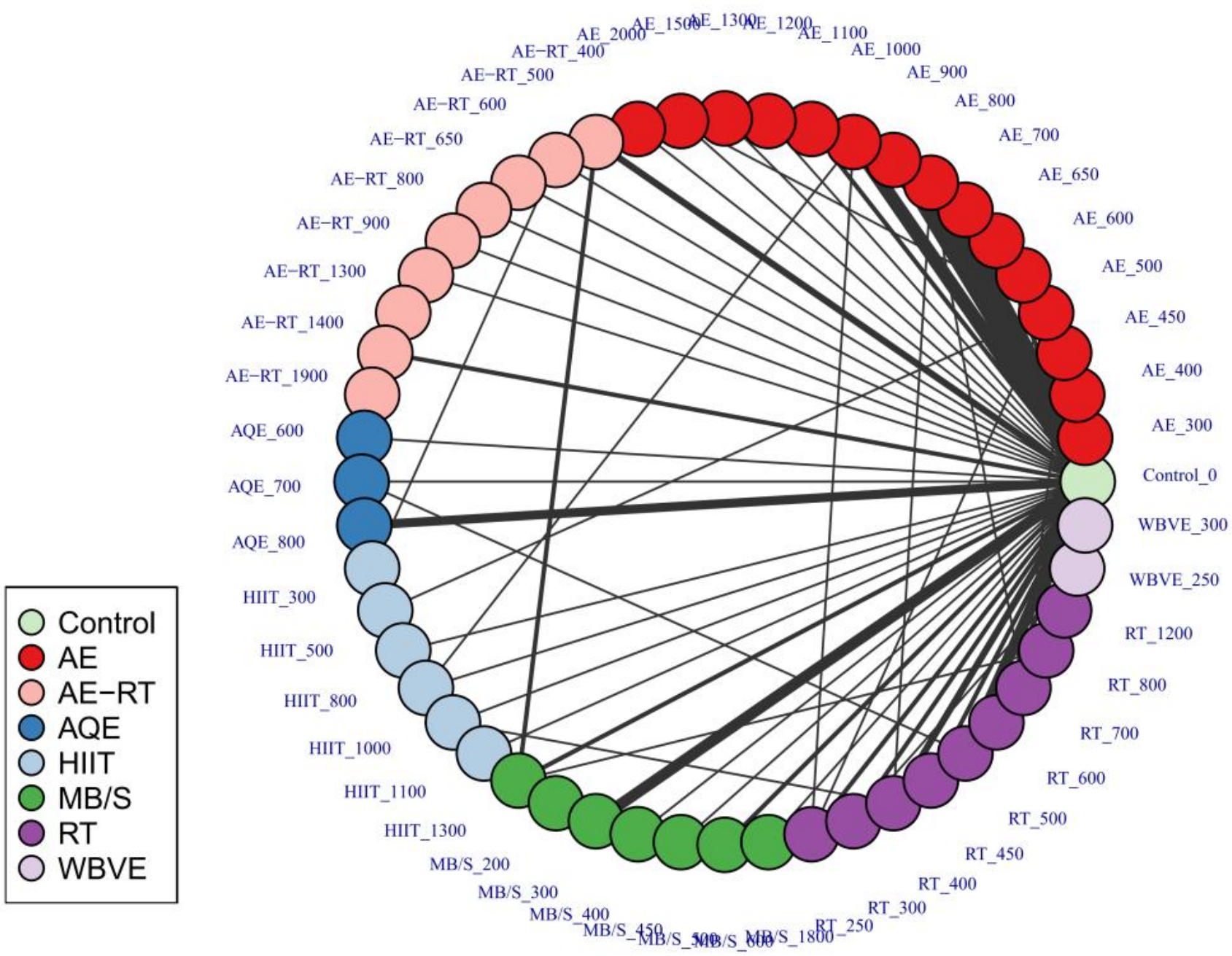

Figure S3. Dose-level network (systolic blood pressure).

Line thickness represents the number of studies available for comparison. The first value represents the specific intervention, and the second value corresponds to the dose (METs - min/week).

AE: Aerobic Exercise; AE-RT: Combined Aerobic and Resistance Training; RT: Resistance Training;MB/S:Mind-body exercise or stretching;HIIT: High-Intensity Interval Training;AQE:Aquatic Exercise; WBVE:Whole-Body Vibration Exercise;CG: Control group

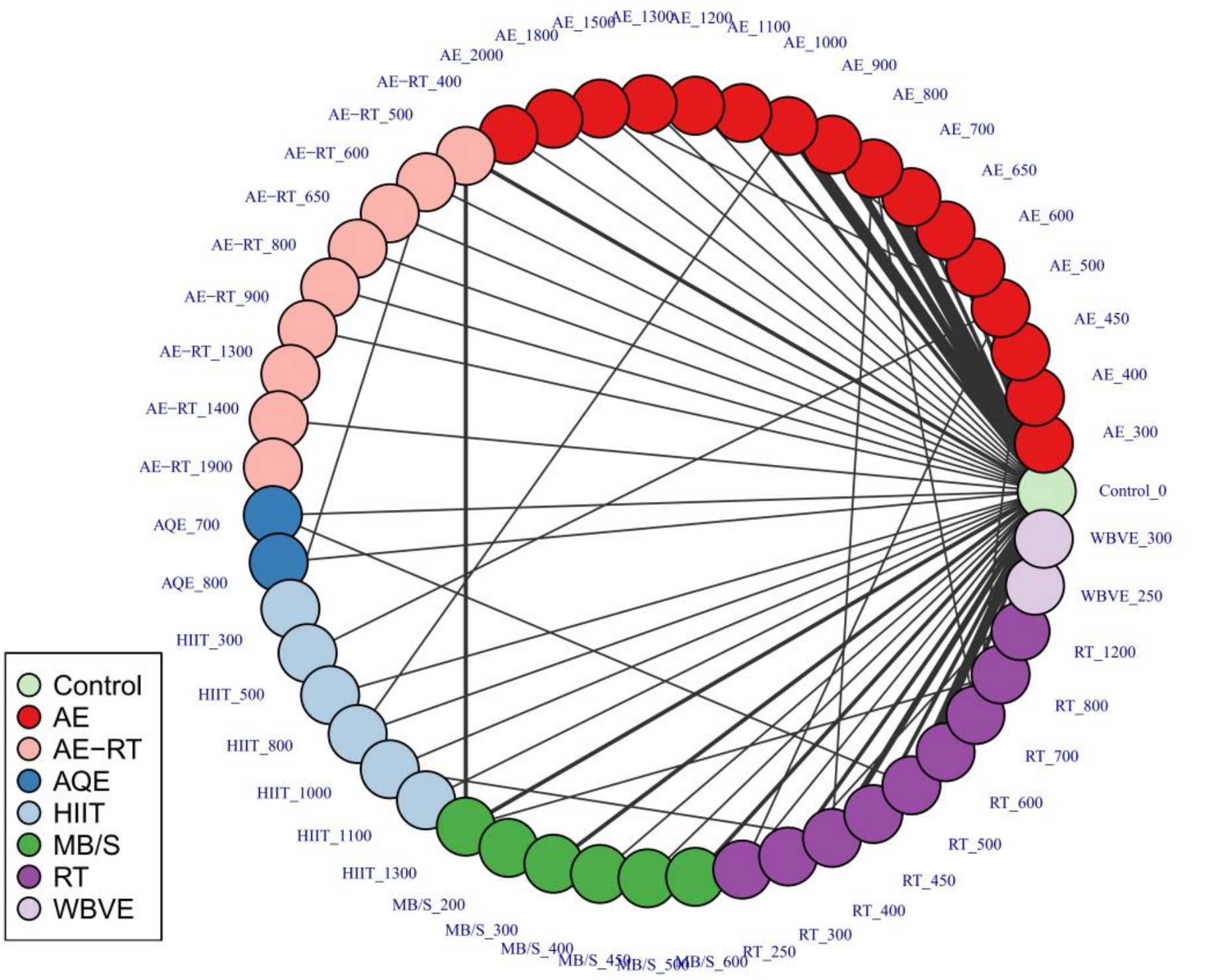

Figure S4. Dose-level network (diastolic blood pressure)

Line thickness represents the number of studies available for comparison. The first value represents the specific intervention, and the second value corresponds to the dose (METs-min/week).

AE: Aerobic Exercise; AE-RT: Combined Aerobic and Resistance Training; RT: Resistance Training;MB/S:Mind-body exercise or stretching;HIIT: High-Intensity Interval Training;AQE:Aquatic Exercise;WBVE:Whole-Body Vibration Exercise;CG: Control group

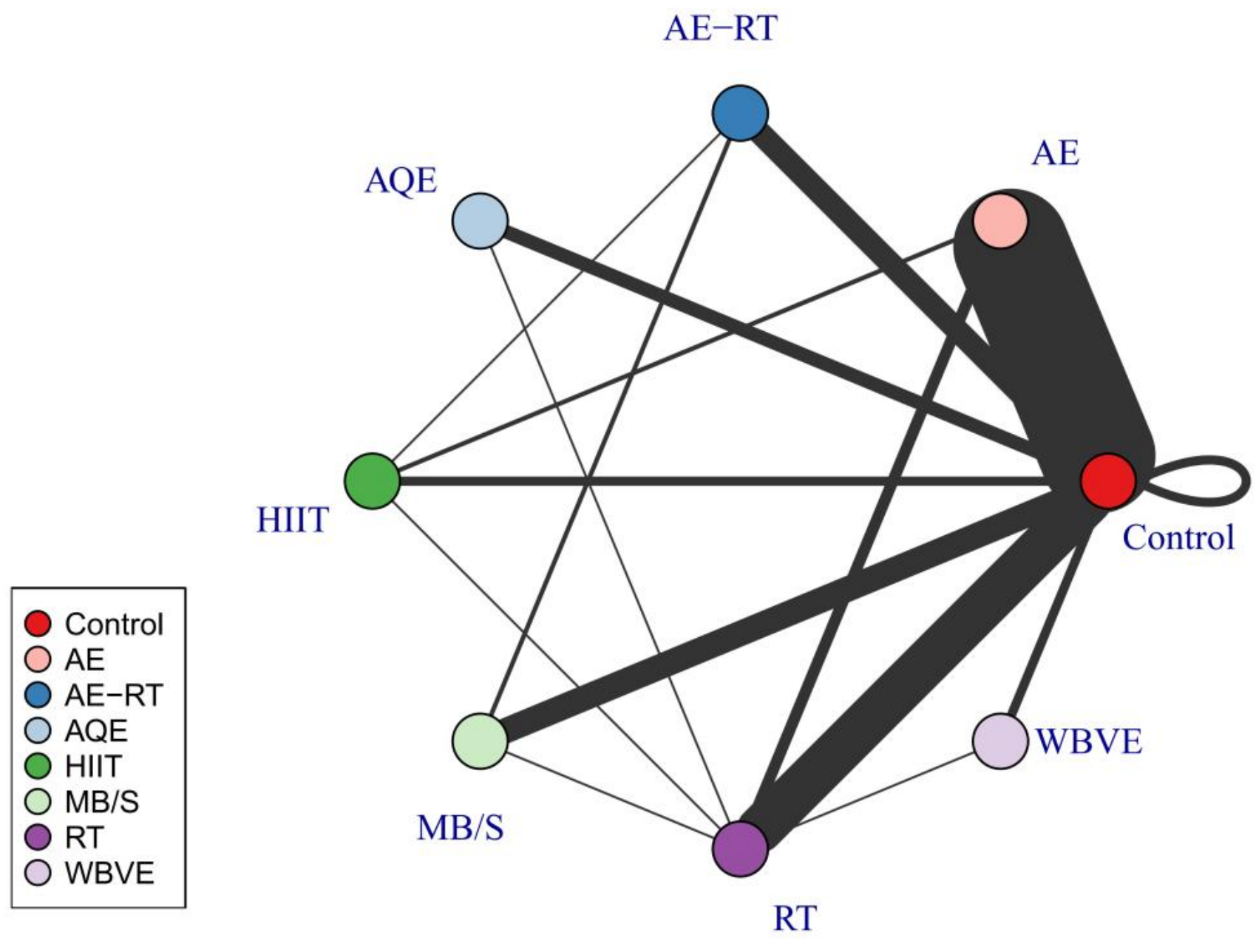

Figure S5. Agent-level network (systolic blood pressure).

Line thickness represents the number of studies available for comparison.

AE: Aerobic Exercise; AE-RT: Combined Aerobic and Resistance Training; RT: Resistance Training;MB/S:Mind-body exercise or stretching;HIIT: High-Intensity Interval Training;AQE:Aquatic Exercise;WBVE:Whole-Body Vibration Exercise;CG: Control group

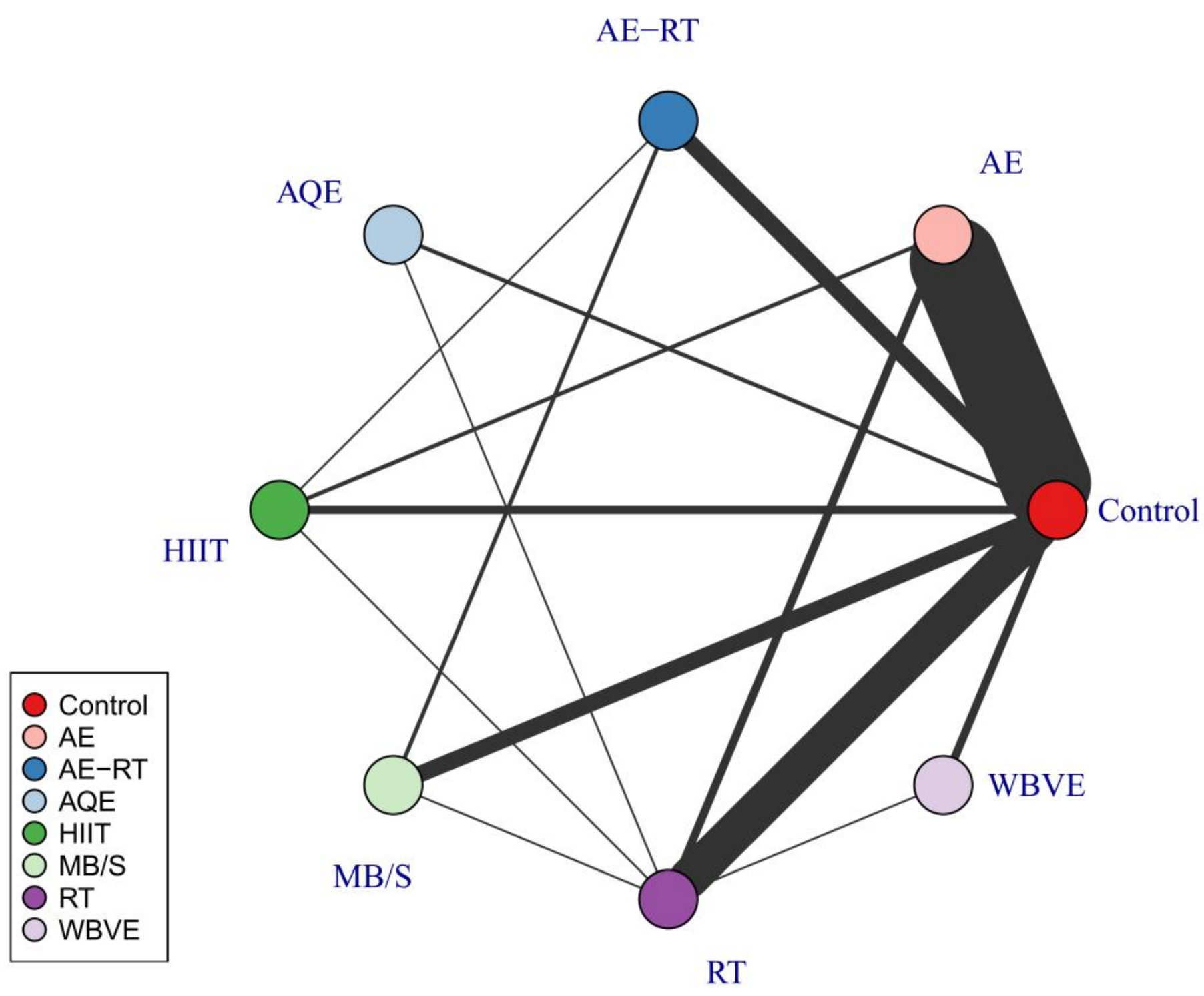

Figure S6. Agent-level network (diastolic blood pressure).

Line thickness represents the number of studies available for comparison.

AE: Aerobic Exercise; AE-RT: Combined Aerobic and Resistance Training; RT: Resistance Training;MB/S:Mind-body exercise or stretching;HIIT: High-Intensity Interval Training;AQE:Aquatic Exercise;WBVE:Whole-Body Vibration Exercise;CG: Control group

7.2 Consistency

We performed a consistency analysis of the data by comparing the consistency of the network i.e., network effect size with the unrelated mean effects UME model i.e., pairwise effect sizes. In practice, we checked whether the bias, the number of estimated parameters in the network, and the Deviance Information Criterion DIC metric were similar for both models, indicating a good fit. The comparison showed good agreement between the models (Tables S5 and S6). Local consistency was further assessed using node-splitting, which separated direct and indirect evidence for each available treatment contrast. The results are presented in Tables S7 and S8.

Table S5. Systolic blood pressure Comparison of consistency and UME model fit.

Model	PD	Deviance	Residual deviance	DIC	SD
Consistent	170.1	615.677	195.531	786.0	4.912
UME	170.3	617.274	197.128	787.5	4.820

Note: UME = unrelated mean effects model; DIC = deviance information criterion; PD = effective number of parameters; SD = standard deviation

Table S6. Diastolic blood pressure Comparison of consistency and UME model fit.

Model	PD	Deviance	Residual deviance	DIC	SD
Consistent	164.9	450.598	182.570	614.7	4.692
UME	165.2	451.510	183.481	615.8	4.626

Note: UME = unrelated mean effects model; DIC = deviance information criterion; PD = effective number of parameters; SD = standard deviation

Table S7. Systolic blood pressure Node-splitting analysis of consistency.

==

Comparison	p-value	Median	2.5%	97.5%

RT_800 vs MB/S_200	**0.088**			
-> direct		-7.923	-19.473	3.114
-> indirect		5.207	0.804	9.674
-> MBNMA		3.598	-0.394	7.599
MB/S_300 vs MB/S_200	**0.124**			
-> direct		-2.950	-14.592	8.533
-> indirect		-0.650	-1.471	-0.039
-> MBNMA		-0.647	-1.480	-0.037
AE-RT_1900 vs AE-RT_650	**0.073**			
-> direct		4.615	-4.181	13.304
-> indirect		-0.337	-1.253	-0.014
-> MBNMA		-0.332	-1.273	-0.014
AE-RT_1300 vs AE-RT_650	**0.033**			
-> direct		7.268	-2.529	17.144
-> indirect		-0.269	-0.973	-0.012
-> MBNMA		-0.248	-0.926	-0.010
HIIT_300 vs AE-RT_500	**0.656**			
-> direct		0.689	-9.008	10.935
-> indirect		-1.780	-7.252	3.859
-> MBNMA		-1.325	-6.093	3.558
RT_400 vs AE_800	**0.328**			
-> direct		-0.589	-15.457	14.596
-> indirect		0.962	-1.799	3.754
-> MBNMA		1.003	-1.591	3.613
HIIT_500 vs AE_500	**0.511**			
-> direct		-0.113	-12.742	13.407
-> indirect		-2.949	-7.585	1.764
-> MBNMA		-2.735	-6.811	1.556
WBVE_300 vs Control	**0.148**			
-> direct		-8.724	-16.490	-0.564
-> indirect		2.599	-4.762	9.749
-> MBNMA		-2.436	-7.736	2.775
WBVE_250 vs Control	**0.073**			
-> direct		4.687	-1.842	11.429
-> indirect		-8.401	-16.134	-0.933
-> MBNMA		-2.326	-7.422	2.678

RT_1200 vs Control	**0.259**			
-> direct		1.247	-14.142	16.297
-> indirect		-4.266	-7.033	-1.663
-> MBNMA		-4.106	-6.867	-1.533
RT_700 vs Control	**0.461**			
-> direct		-6.759	-13.523	-0.059
-> indirect		-3.990	-6.697	-1.270
-> MBNMA		-3.922	-6.473	-1.457
RT_600 vs Control	**0.496**			
-> direct		-5.130	-12.812	2.485
-> indirect		-3.692	-6.280	-1.168
-> MBNMA		-3.858	-6.292	-1.414
RT_500 vs Control	**0.257**			
-> direct		-0.586	-5.242	4.244
-> indirect		-4.825	-7.585	-2.088
-> MBNMA		-3.771	-6.145	-1.376
RT_450 vs Control	**0.561**			
-> direct		-3.911	-10.314	2.742
-> indirect		-3.740	-6.219	-1.349
-> MBNMA		-3.710	-6.015	-1.351
RT_300 vs Control	**0.102**			
-> direct		3.462	-3.762	10.762
-> indirect		-4.140	-6.416	-1.878
-> MBNMA		-3.431	-5.547	-1.239
RT_250 vs Control	**0.536**			
-> direct		-3.248	-9.817	3.327
-> indirect		-3.283	-5.571	-1.067
-> MBNMA		-3.278	-5.352	-1.178
MB/S_600 vs Control	**0.630**			
-> direct		-7.409	-14.208	-0.337
-> indirect		-9.864	-14.456	-5.503
-> MBNMA		-9.121	-12.928	-5.397
MB/S_500 vs Control	**0.510**			
-> direct		-6.461	-16.937	4.049
-> indirect		-9.310	-13.336	-5.325

-> MBNMA		-8.927	-12.535	-5.299
MB/S_450 vs Control	**0.526**			
-> direct		-10.869	-20.766	-0.978
-> indirect		-8.488	-12.301	-4.749
-> MBNMA		-8.826	-12.335	-5.225
MB/S_400 vs Control	**0.732**			
-> direct		-8.402	-15.867	-1.440
-> indirect		-8.836	-12.898	-4.739
-> MBNMA		-8.688	-12.146	-5.153
HIIT_1300 vs Control	**0.359**			
-> direct		-13.000	-23.118	-3.075
-> indirect		-6.413	-11.562	-1.720
-> MBNMA		-7.876	-12.098	-3.150
HIIT_1100 vs Control	**0.592**			
-> direct		-9.907	-20.119	0.161
-> indirect		-7.237	-11.967	-2.341
-> MBNMA		-7.771	-11.945	-3.098
HIIT_1000 vs Control	**0.752**			
-> direct		-6.991	-15.476	1.659
-> indirect		-7.293	-12.588	-2.016
-> MBNMA		-7.713	-11.807	-3.064
HIIT_800 vs Control	**0.259**			
-> direct		1.208	-14.166	15.663
-> indirect		-8.169	-12.524	-3.808
-> MBNMA		-7.554	-11.572	-3.015
AQE_800 vs Control	**0.838**			
-> direct		-12.048	-21.888	-1.913
-> indirect		-9.923	-19.895	0.133
-> MBNMA		-11.054	-18.186	-3.778
AQE_700 vs Control	**0.409**			
-> direct		-4.117	-18.962	10.753
-> indirect		-12.655	-20.473	-5.274
-> MBNMA		-10.901	-17.947	-3.759
AQE_600 vs Control	**0.639**			
-> direct		-12.936	-28.748	2.761

-> indirect		-10.126	-17.789	-2.489
-> MBNMA		-10.739	-17.588	-3.701
AE-RT_1400 vs Control	**0.692**			
-> direct		-5.202	-13.656	2.768
-> indirect		-5.903	-10.023	-1.725
-> MBNMA		-5.707	-9.476	-1.888
AE-RT_900 vs Control	**0.209**			
-> direct		-12.793	-22.427	-3.191
-> indirect		-4.520	-8.481	-0.627
-> MBNMA		-5.552	-9.181	-1.871
AE-RT_800 vs Control	**0.540**			
-> direct		-5.629	-16.902	5.067
-> indirect		-5.461	-9.500	-1.502
-> MBNMA		-5.493	-9.082	-1.855
AE-RT_650 vs Control	**0.189**			
-> direct		6.138	-13.614	25.967
-> indirect		-5.772	-9.397	-2.259
-> MBNMA		-5.379	-8.888	-1.810
AE-RT_600 vs Control	**0.399**			
-> direct		-1.297	-12.340	9.602
-> indirect		-5.795	-9.541	-2.208
-> MBNMA		-5.327	-8.846	-1.809
AE-RT_500 vs Control	**0.470**			
-> direct		-8.073	-18.268	2.190
-> indirect		-4.822	-8.361	-1.112
-> MBNMA		-5.210	-8.698	-1.805
AE-RT_400 vs Control	**0.610**			
-> direct		-6.557	-14.584	1.007
-> indirect		-4.741	-8.463	-1.014
-> MBNMA		-5.041	-8.471	-1.751
AE_2000 vs Control	**0.106**			
-> direct		-12.857	-22.813	-3.123
-> indirect		-4.542	-6.404	-2.774
-> MBNMA		-4.864	-6.684	-3.097
AE_1800 vs Control	**0.196**			

-> direct		-10.450	-20.949	-0.168
-> indirect		-4.675	-6.555	-2.886
-> MBNMA		-4.844	-6.654	-3.089
AE_1500 vs Control	**0.342**			
-> direct		-3.322	-11.804	5.133
-> indirect		-4.789	-6.627	-3.121
-> MBNMA		-4.795	-6.572	-3.074
AE_1300 vs Control	**0.072**			
-> direct		5.068	-5.255	14.864
-> indirect		-5.070	-6.847	-3.347
-> MBNMA		-4.764	-6.516	-3.053
AE_1200 vs Control	**0.339**			
-> direct		-3.262	-12.801	5.789
-> indirect		-4.781	-6.566	-3.030
-> MBNMA		-4.742	-6.473	-3.040
AE_1100 vs Control	**0.220**			
-> direct		-9.302	-17.869	-1.082
-> indirect		-4.525	-6.337	-2.751
-> MBNMA		-4.718	-6.432	-3.028
AE_1000 vs Control	**0.371**			
-> direct		-2.513	-6.521	1.865
-> indirect		-5.009	-6.861	-3.199
-> MBNMA		-4.697	-6.361	-3.002
AE_900 vs Control	**0.427**			
-> direct		-6.471	-11.325	-1.336
-> indirect		-4.477	-6.264	-2.678
-> MBNMA		-4.660	-6.323	-2.983
AE_700 vs Control	**0.404**			
-> direct		-4.798	-12.170	2.488
-> indirect		-4.595	-6.314	-2.822
-> MBNMA		-4.565	-6.163	-2.937
AE_650 vs Control	**0.245**			
-> direct		-0.724	-8.993	7.725
-> indirect		-4.688	-6.454	-3.012
-> MBNMA		-4.534	-6.116	-2.920

AE_600 vs Control	**0.512**			
-> direct		-3.548	-8.450	1.415
-> indirect		-4.652	-6.373	-2.894
-> MBNMA		-4.495	-6.082	-2.899
AE_500 vs Control	**0.373**			
-> direct		-2.241	-8.374	3.836
-> indirect		-4.528	-6.218	-2.867
-> MBNMA		-4.397	-5.978	-2.831
AE_450 vs Control	**0.407**			
-> direct		-5.623	-11.761	0.652
-> indirect		-4.247	-5.916	-2.649
-> MBNMA		-4.346	-5.929	-2.787
AE_400 vs Control	**0.446**			
-> direct		-5.714	-11.050	-0.762
-> indirect		-4.085	-5.795	-2.425
-> MBNMA		-4.270	-5.867	-2.730
AE_300 vs Control	**0.017**			
-> direct		-20.738	-33.235	-7.910
-> indirect		-3.764	-5.485	-2.281
-> MBNMA		-4.054	-5.709	-2.558

Note: The first value represents the specific intervention, and the second value corresponds to the dose (METs-min/week).
AE: Aerobic Exercise; AE-RT: Combined Aerobic and Resistance Training; RT: Resistance Training;MB/S:Mind-body exercise or stretching;HIIT: High-Intensity Interval Training;AQE:Aquatic Exercise;WBVE:Whole-Body Vibration Exercise;CG: Control group

Table S8. Diastolic blood pressure Node-splitting analysis of consistency.

==

Comparison	p-value	Median	2.5%	97.5%
RT_800 vs MB/S_200	**0.311**			
-> direct		-2.946	-12.780	6.982
-> indirect		3.365	-0.572	7.352
-> MBNMA		2.575	-0.889	6.186
MB/S_300 vs MB/S_200	**0.092**			
-> direct		-3.198	-13.114	6.730
-> indirect		-0.322	-0.958	-0.015
-> MBNMA		-0.328	-0.949	-0.016

AE-RT_1900 vs AE-RT_650	**0.089**			
-> direct		3.840	-4.120	11.182
-> indirect		-0.286	-1.121	-0.010
-> MBNMA		-0.270	-1.074	-0.010
AE-RT_1300 vs AE-RT_650	**0.088**			
-> direct		1.959	-6.918	10.934
-> indirect		-0.219	-0.812	-0.007
-> MBNMA		-0.202	-0.781	-0.007
HIIT_300 vs AE-RT_500	**0.508**			
-> direct		3.698	-5.522	12.972
-> indirect		-0.425	-5.193	4.443
-> MBNMA		0.487	-4.053	4.959
RT_400 vs AE_800	**0.346**			
-> direct		-2.474	-12.995	7.796
-> indirect		0.589	-1.879	3.035
-> MBNMA		0.497	-1.937	2.969
RT_250 vs AE_500	**0.340**			
-> direct		-2.807	-11.704	6.313
-> indirect		0.705	-1.573	3.042
-> MBNMA		0.580	-1.718	2.847
HIIT_500 vs AE_500	**0.405**			
-> direct		3.160	-7.019	13.114
-> indirect		-1.897	-6.074	1.955
-> MBNMA		-1.262	-4.942	2.595
WBVE_300 vs Control	**0.226**			
-> direct		-5.883	-12.768	1.193
-> indirect		2.256	-3.764	8.347
-> MBNMA		-1.405	-5.993	3.224
WBVE_250 vs Control	**0.200**			
-> direct		2.762	-2.956	8.584
-> indirect		-5.484	-12.278	1.130
-> MBNMA		-1.344	-5.678	3.123
RT_1200 vs Control	**0.353**			
-> direct		-1.513	-13.007	10.162
-> indirect		-2.695	-5.074	-0.384
-> MBNMA		-2.571	-4.993	-0.349

RT_700 vs Control	**0.449**			
-> direct		-4.974	-10.451	0.710
-> indirect		-2.396	-4.670	0.073
-> MBNMA		-2.460	-4.704	-0.327
RT_600 vs Control	**0.498**			
-> direct		-1.404	-8.029	5.369
-> indirect		-2.600	-4.910	-0.400
-> MBNMA		-2.418	-4.631	-0.311
RT_500 vs Control	**0.688**			
-> direct		-2.000	-6.460	2.261
-> indirect		-2.454	-4.904	-0.098
-> MBNMA		-2.368	-4.504	-0.301
RT_450 vs Control	**0.560**			
-> direct		-2.467	-8.595	3.005
-> indirect		-2.360	-4.603	-0.162
-> MBNMA		-2.336	-4.437	-0.299
RT_300 vs Control	**0.407**			
-> direct		-0.109	-6.403	5.805
-> indirect		-2.431	-4.489	-0.410
-> MBNMA		-2.171	-4.166	-0.266
MB/S_600 vs Control	**0.018**			
-> direct		2.802	-3.148	8.665
-> indirect		-9.117	-12.782	-5.484
-> MBNMA		-5.984	-9.282	-2.812
MB/S_500 vs Control	**0.547**			
-> direct		-4.759	-13.810	3.700
-> indirect		-6.028	-9.418	-2.749
-> MBNMA		-5.896	-9.103	-2.767
MB/S_450 vs Control	**0.388**			
-> direct		-9.694	-18.637	-0.502
-> indirect		-5.259	-8.538	-1.760
-> MBNMA		-5.830	-8.987	-2.713
MB/S_400 vs Control	**0.716**			
-> direct		-6.299	-12.857	0.139
-> indirect		-5.535	-9.129	-1.972

-> MBNMA		-5.745	-8.883	-2.665
HIIT_1300 vs Control	**0.561**			
-> direct		-6.585	-15.963	2.917
-> indirect		-3.788	-8.142	0.331
-> MBNMA		-4.290	-8.341	-0.212
HIIT_1100 vs Control	**0.264**			
-> direct		-10.279	-19.425	-0.855
-> indirect		-3.069	-7.291	1.199
-> MBNMA		-4.237	-8.237	-0.210
HIIT_1000 vs Control	**0.745**			
-> direct		-3.898	-11.783	3.677
-> indirect		-4.015	-8.561	0.474
-> MBNMA		-4.206	-8.193	-0.208
HIIT_800 vs Control	**0.312**			
-> direct		2.070	-9.017	13.269
-> indirect		-5.020	-9.168	-0.728
-> MBNMA		-4.128	-8.001	-0.203
AQE_800 vs Control	**0.742**			
-> direct		-8.826	-18.032	0.133
-> indirect		-5.823	-15.254	3.552
-> MBNMA		-7.448	-13.842	-1.075
AQE_700 vs Control	**0.846**			
-> direct		-7.362	-18.102	3.903
-> indirect		-7.397	-14.874	0.122
-> MBNMA		-7.354	-13.661	-1.069
AE-RT_1400 vs Control	**0.362**			
-> direct		-9.291	-19.388	0.809
-> indirect		-3.732	-7.740	-0.099
-> MBNMA		-4.460	-8.017	-0.856
AE-RT_900 vs Control	**0.068**			
-> direct		-14.559	-23.438	-5.236
-> indirect		-2.761	-6.449	0.803
-> MBNMA		-4.316	-7.823	-0.830
AE-RT_800 vs Control	**0.437**			
-> direct		-1.027	-10.770	8.820

-> indirect		-4.754	-8.397	-1.030
-> MBNMA		-4.268	-7.744	-0.826
AE-RT_650 vs Control	**0.301**			
-> direct		2.023	-10.738	15.069
-> indirect		-4.657	-8.078	-1.200
-> MBNMA		-4.172	-7.605	-0.810
AE-RT_600 vs Control	**0.125**			
-> direct		4.790	-4.809	14.419
-> indirect		-5.235	-8.453	-1.789
-> MBNMA		-4.132	-7.534	-0.801
AE-RT_500 vs Control	**0.510**			
-> direct		-1.984	-11.147	7.091
-> indirect		-4.351	-7.889	-0.980
-> MBNMA		-4.044	-7.361	-0.773
AE-RT_400 vs Control	**0.162**			
-> direct		-10.140	-17.829	-2.439
-> indirect		-2.790	-6.083	0.767
-> MBNMA		-3.912	-7.188	-0.758
AE_2000 vs Control	**0.157**			
-> direct		-8.960	-17.921	-0.026
-> indirect		-2.755	-4.341	-1.148
-> MBNMA		-2.940	-4.627	-1.378
AE_1800 vs Control	**0.149**			
-> direct		-9.277	-18.501	0.020
-> indirect		-2.722	-4.364	-1.190
-> MBNMA		-2.930	-4.607	-1.371
AE_1500 vs Control	**0.280**			
-> direct		0.255	-7.744	8.032
-> indirect		-2.951	-4.563	-1.329
-> MBNMA		-2.904	-4.553	-1.358
AE_1300 vs Control	**0.164**			
-> direct		2.812	-5.838	11.827
-> indirect		-3.063	-4.602	-1.479
-> MBNMA		-2.888	-4.502	-1.344
AE_1200 vs Control	**0.337**			

-> direct		-2.973	-10.766	5.056
-> indirect		-2.867	-4.372	-1.316
-> MBNMA		-2.877	-4.486	-1.336
AE_1100 vs Control	**0.381**			
-> direct		-3.855	-11.173	2.798
-> indirect		-2.811	-4.362	-1.209
-> MBNMA		-2.864	-4.455	-1.327
AE_1000 vs Control	**0.369**			
-> direct		-0.872	-4.642	2.995
-> indirect		-3.081	-4.665	-1.515
-> MBNMA		-2.841	-4.400	-1.324
AE_900 vs Control	**0.517**			
-> direct		-2.041	-6.519	2.352
-> indirect		-2.840	-4.349	-1.280
-> MBNMA		-2.822	-4.375	-1.321
AE_700 vs Control	**0.229**			
-> direct		1.005	-5.306	7.451
-> indirect		-2.941	-4.380	-1.467
-> MBNMA		-2.768	-4.275	-1.294
AE_650 vs Control	**0.395**			
-> direct		-3.411	-10.129	2.953
-> indirect		-2.686	-4.213	-1.131
-> MBNMA		-2.750	-4.235	-1.276
AE_600 vs Control	**0.399**			
-> direct		-0.890	-4.989	3.509
-> indirect		-2.945	-4.460	-1.398
-> MBNMA		-2.727	-4.193	-1.269
AE_450 vs Control	**0.328**			
-> direct		-4.826	-10.024	0.480
-> indirect		-2.469	-3.880	-1.046
-> MBNMA		-2.631	-4.060	-1.211
AE_400 vs Control	**0.501**			
-> direct		-3.166	-7.819	1.253
-> indirect		-2.523	-4.008	-1.002
-> MBNMA		-2.586	-4.008	-1.187

AE_300 vs Control	**0.057**			
-> direct		-11.750	-21.724	-2.165
-> indirect		-2.262	-3.608	-1.034
-> MBNMA		-2.459	-3.905	-1.139

Note: The first value represents the specific intervention, and the second value corresponds to the dose (METs-min/week). AE: Aerobic Exercise; AE-RT: Combined Aerobic and Resistance Training; RT: Resistance Training;MB/S:Mind-body exercise or stretching;HIIT: High-Intensity Interval Training;AQE:Aquatic Exercise;WBVE:Whole-Body Vibration Exercise;CG: Control group

7.3 Transitivity

Transitivity was assessed qualitatively by comparing the available study-level distributions of potential effect modifiers across exercise-modality comparisons. The factors considered included mean participant age, menopausal stage, baseline SBP and DBP, intervention duration, comorbidity profile, and control-group characteristics. The major connected comparisons showed broadly overlapping participant and intervention characteristics, and no clear systematic imbalance in these effect modifiers was identified. However, evidence for AQE and WBVE was sparse, and reporting of several study-level characteristics was incomplete. Therefore, although no major violation of the transitivity assumption was identified, residual intransitivity cannot be excluded.

7.4 Model selection (systolic blood pressure as an example)

For model selection, we adopted a quadratic function model. In accordance with the default settings in MBNMAdose, we applied vague (non-informative) prior distributions to minimize bias and allow the data to drive the results, thereby enabling the model to adapt flexibly to the underlying data structure. This approach is designed to capture a specific form of non-linear dose–response relationship and to identify an optimal dose under the assumption of a quadratic association. Below, we present a comparison of parameter estimates across different models (Table S9). In general, lower DIC indicate a better model fit. As the quadratic function model exhibited the lowest DIC value, we selected it for our analysis.Although the quadratic function yielded the lowest DIC, the estimated turning point remains conditional on the selected functional form and should be interpreted as a model-derived peak within the observed dose range rather than as a fixed biological threshold.

Table S9. Models fit comparison (systolic blood pressure)

Model	DIC	SD	Deviance	Residual deviance	pD
Emax (random treatment effects)	812.7	4.948	642.708	179.690	169.7

Restricted cubic spline (random treatment effects; 3 knots)	818.0	4.895	644.196	196.178	173.3
Log Linear (random treatment effect)	812.4	4.928	642.626	194.608	169.7
Non-parametric monotonically up (random treatment effects)	816.1	4.872	644.194	196.176	172.0
Exponential dose-response function (random treatment effects)	829.6	7.433	638.899	190.881	190.9
Quadratic function (random treatment effects)	811.3	5.204	644.467	196.449	170.7
Spline dose-response functions (random treatment effects)	818.0	4.988	644.925	196.907	172.2
Fractional polynomial dose-response function (random treatment effects)	828.2	6.985	639.519	191.501	190.0

Note: DIC: deviance information criterion; pD: effective number of parameters; SD: standard deviation.

We also validated the fit of the quadratic function model to the data. Figure S7 presents the results of the "Split" network meta-analysis at different doses of various exercises on systolic blood pressure. This step aimed to explore the dose–response pattern and to assess which functional form best fits the data, thereby informing the selection of a model for the

subsequent MBNMA. The model that best matches the pattern shown in Figure S7 should be selected accordingly.

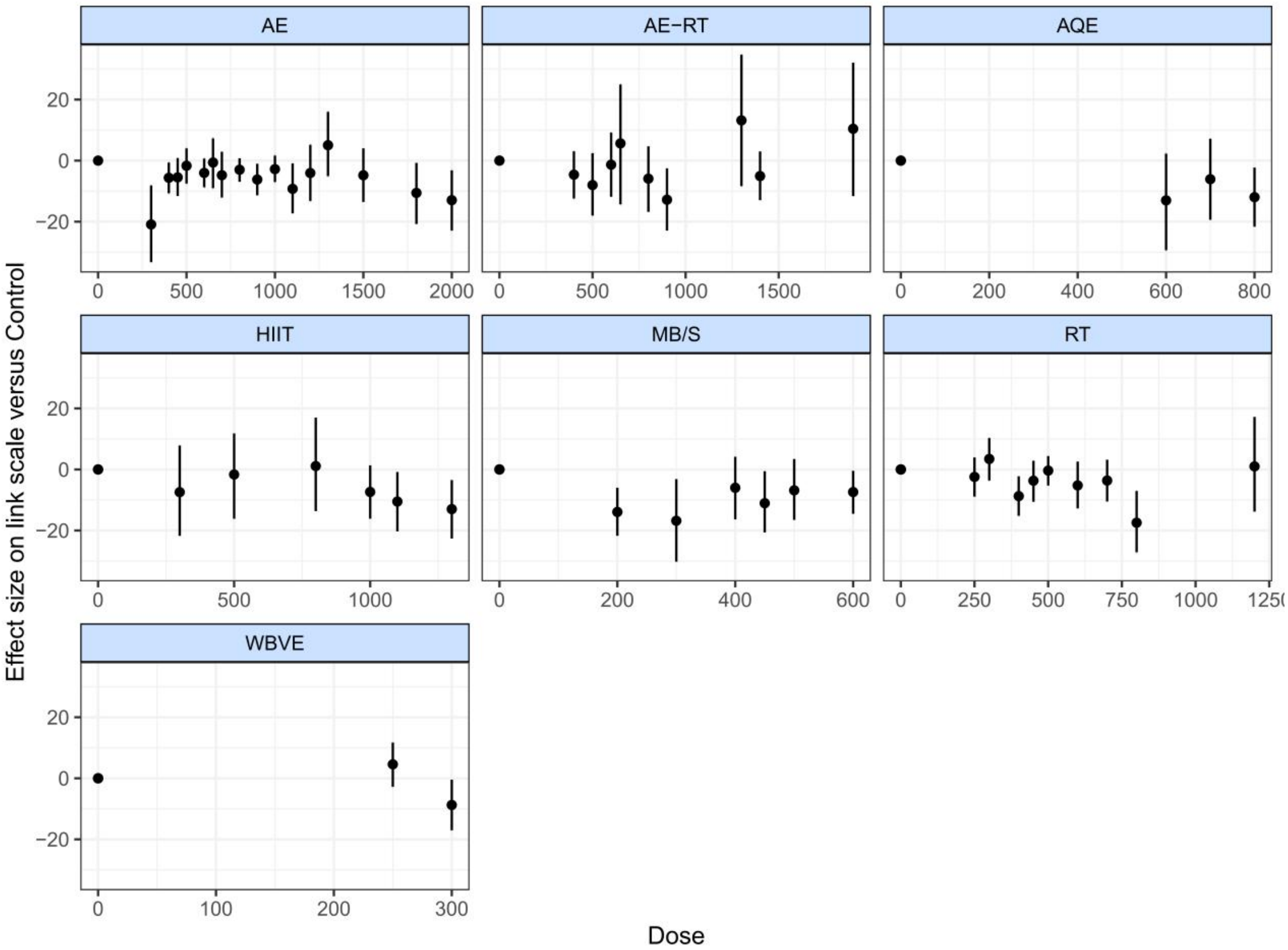

Figure S7. "Split" network meta-analysis of different exercise interventions on systolic blood pressure.

AE: Aerobic Exercise; AE-RT: Combined Aerobic and Resistance Training; RT: Resistance Training;MB/S:Mind-body exercise or stretching;HIIT: High-Intensity Interval Training;AQE:Aquatic Exercise; WBVE:Whole-Body Vibration Exercise;CG: Control group.

We observed that the trend of the model curve is generally consistent with the data points from the "Split" network meta-analysis, suggesting a good fit between the model and the data.

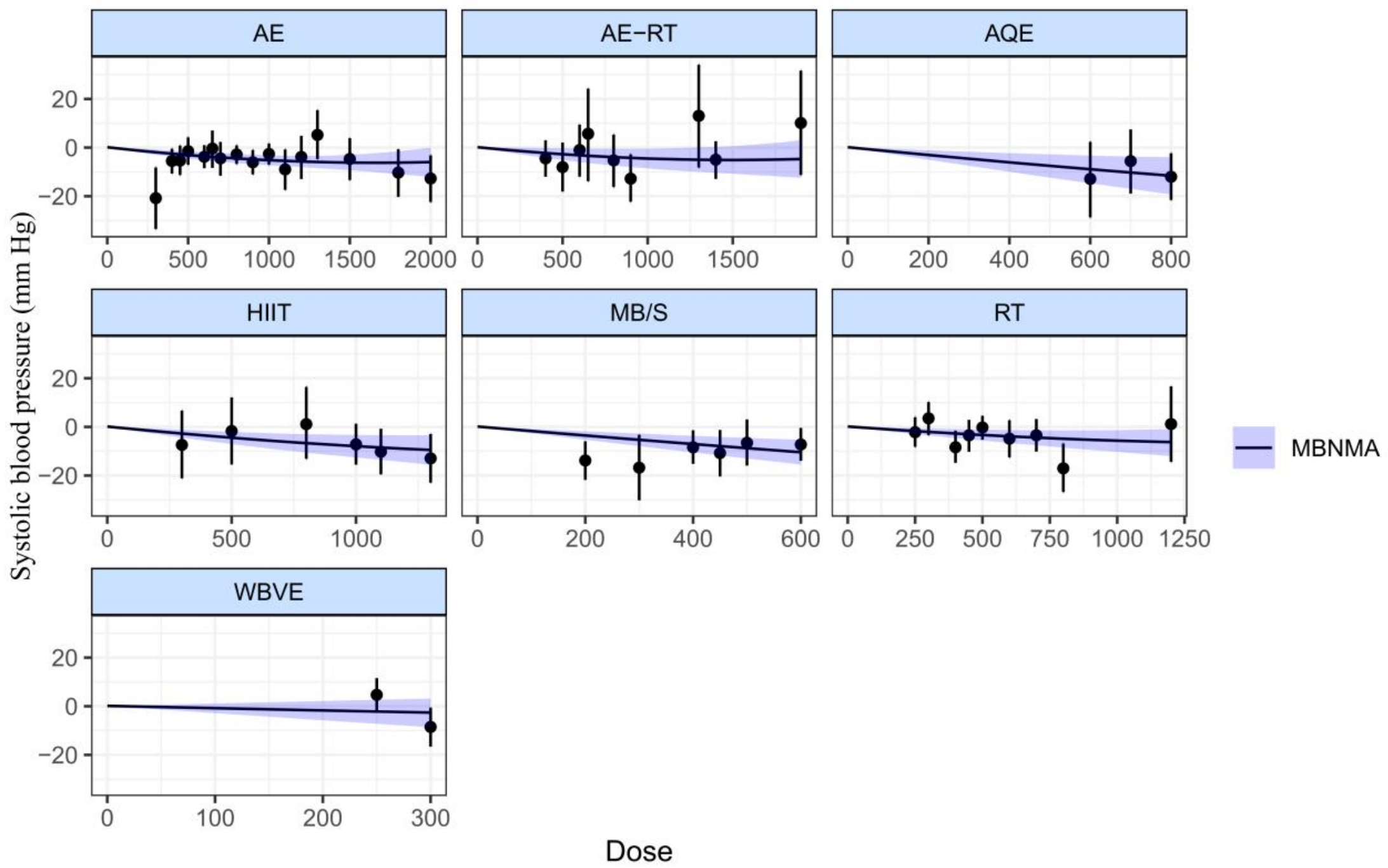

Figure S8. Dose–response relationship between different exercise (METs-min/week) and systolic blood pressure (quadratic function).

The black dots represent the pooled effect size from the meta-analysis at that dose, while the blue region represents the 95% CrI range. AE: Aerobic Exercise; AE-RT: Combined Aerobic and Resistance Training; RT: Resistance Training;MB/S:Mind-body exercise or stretching;HIIT: High-Intensity Interval Training;AQE:Aquatic Exercise; WBVE:Whole-Body Vibration Exercise.

7.5 Subgroup analysis

7.5.1 Systolic blood pressure (baseline blood pressure＞130 mmHg)

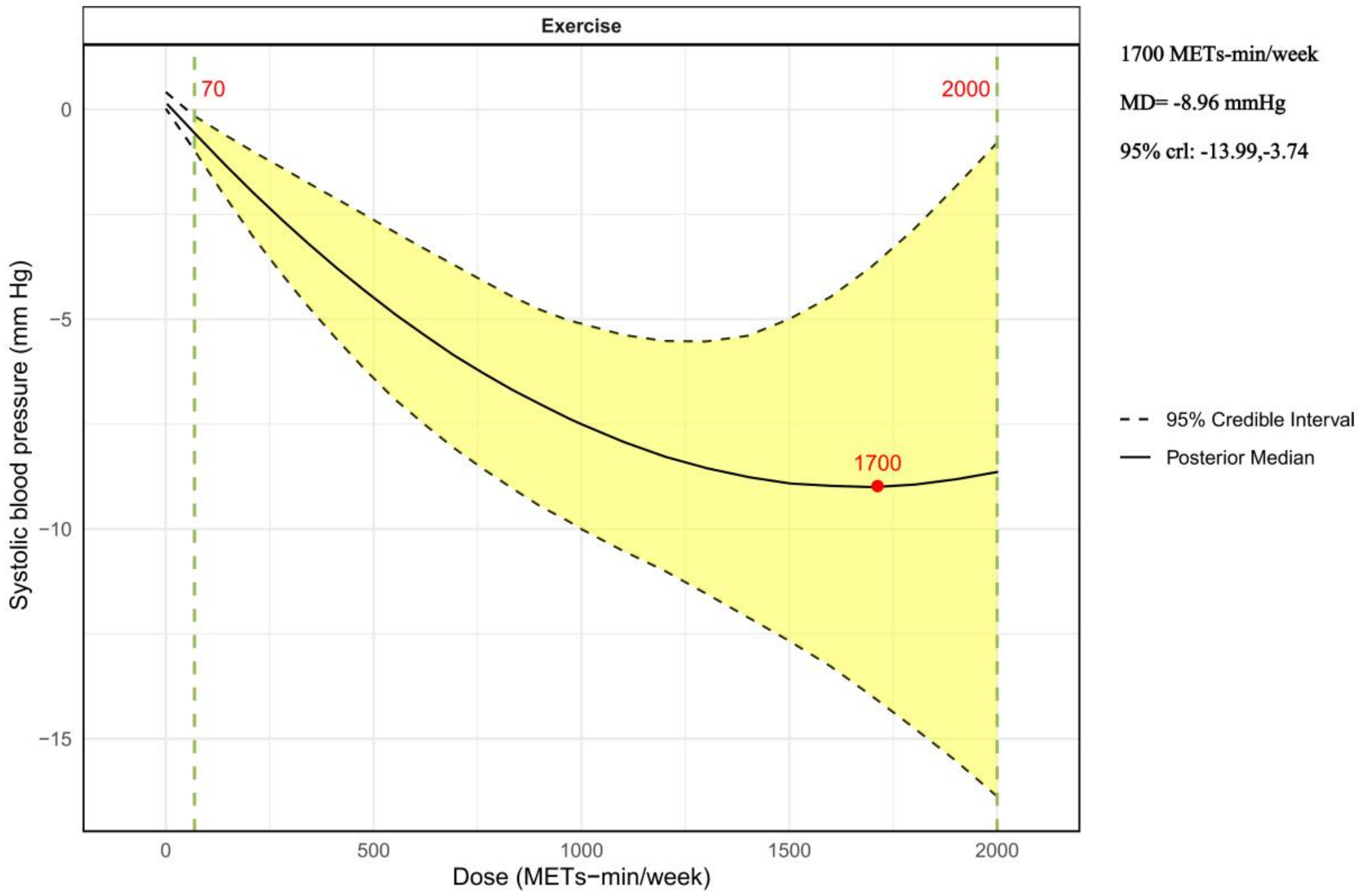

Figure S9. Dose–response relationship between exercise and systolic blood pressure (＞130 mm Hg)

The green dashed lines represent the minimum and maximum effective doses (70 and 2000 METs-min/week), while the yellow shaded area indicates the significant region. and the red dots represent the optimal dose points (1700 METs-min/week).

7.5.2 Systolic blood pressure (baseline blood pressure ≤130 mmHg)

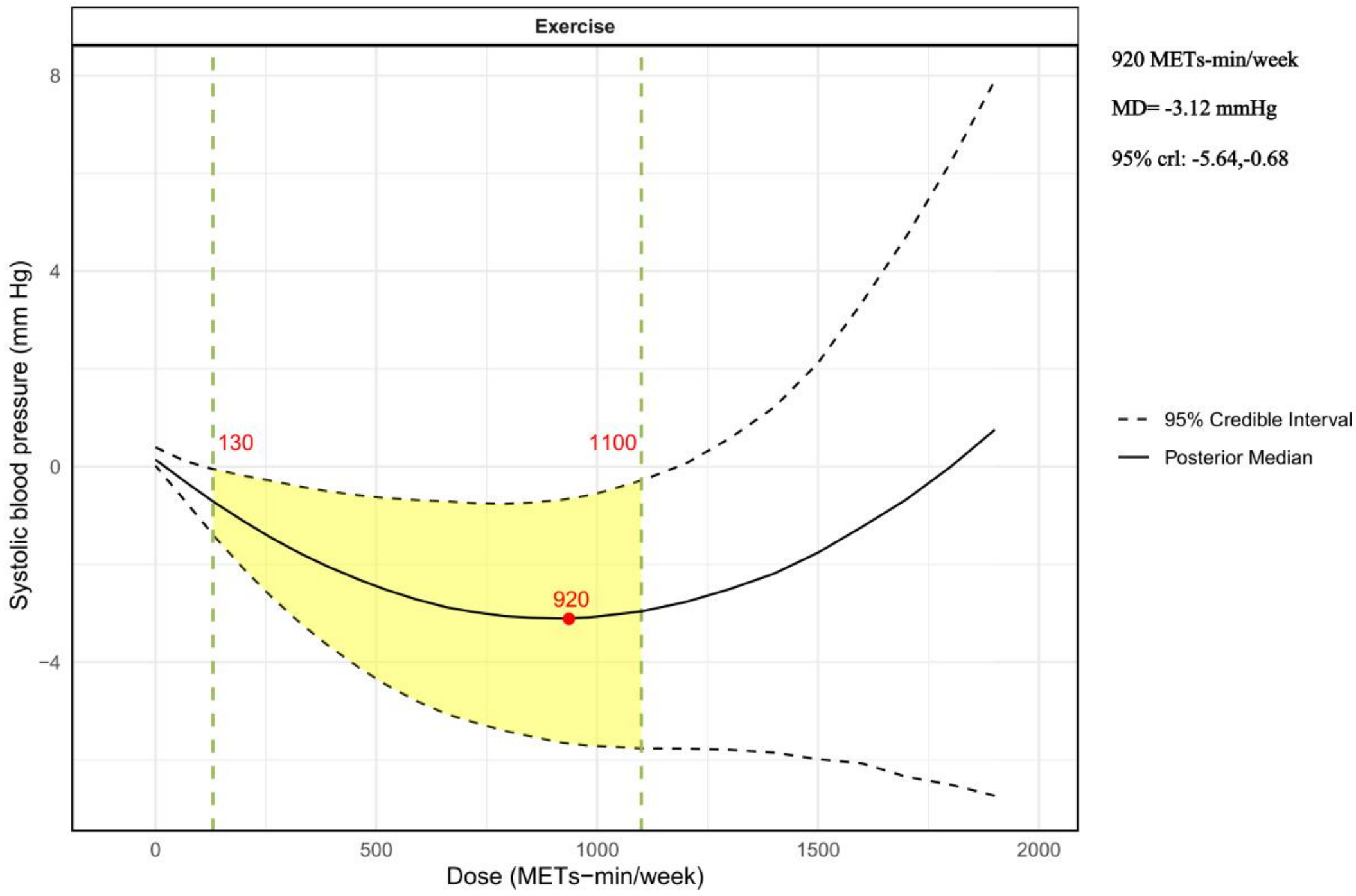

Figure S10. Dose–response relationship between exercise and systolic blood pressure (≤130 mm Hg)

The green dashed lines represent the minimum and maximum effective doses (130 and 1100 METs-min/week), while the yellow shaded area indicates the significant region. and the red dots represent the optimal dose points (920 METs-min/week).

7.5.3 Systolic blood pressure (intervention length＞12 weeks)

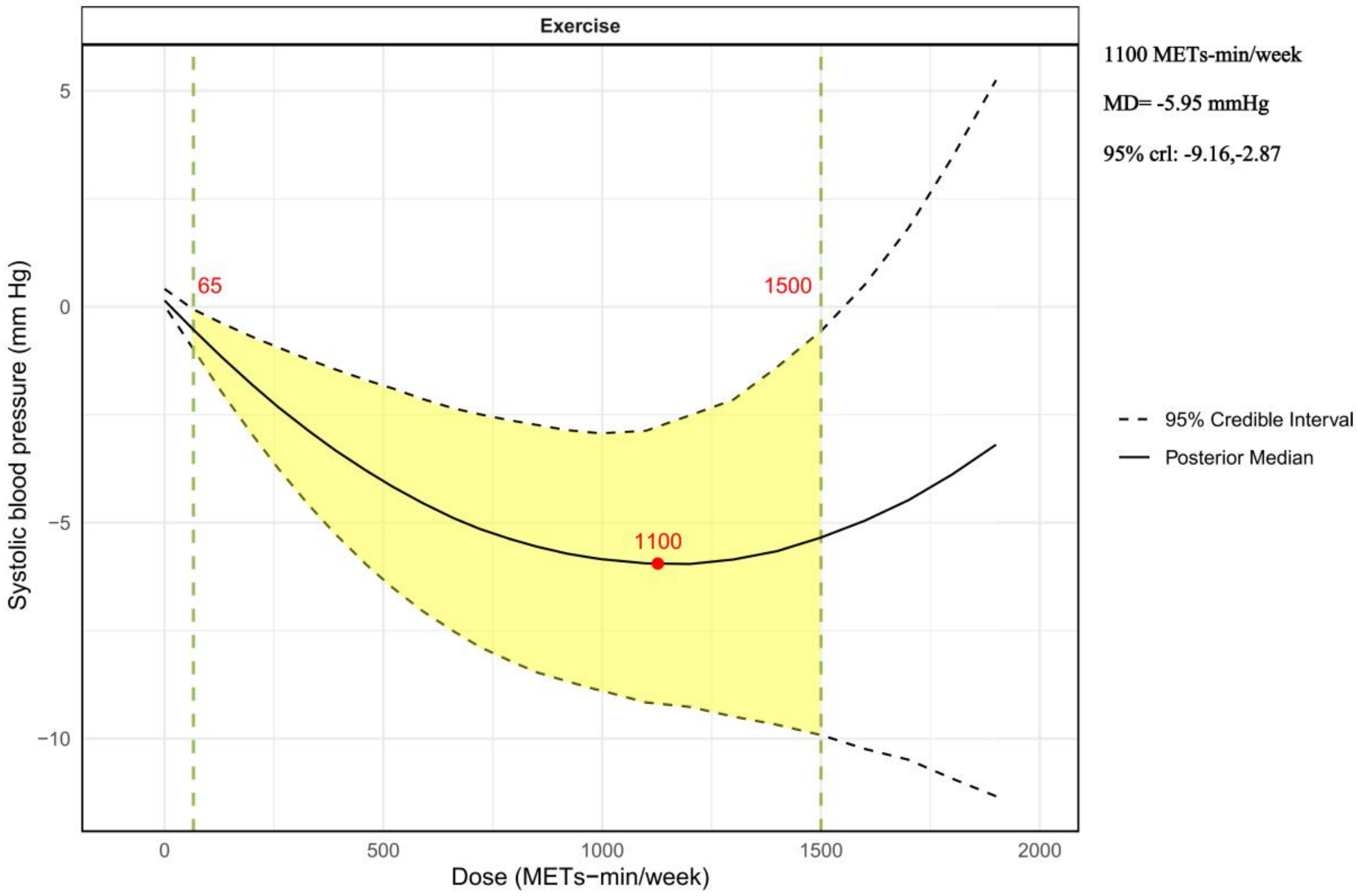

Figure S11. Dose–response relationship between exercise and systolic blood pressure (intervention length ＞12 weeks)

The green dashed lines represent the minimum and maximum effective doses (65 and 1500 METs-min/week), while the yellow shaded area indicates the significant region. and the red dots represent the optimal dose points (1100 METs-min/week).

7.5.4 Systolic blood pressure (intervention length ≤12 weeks)

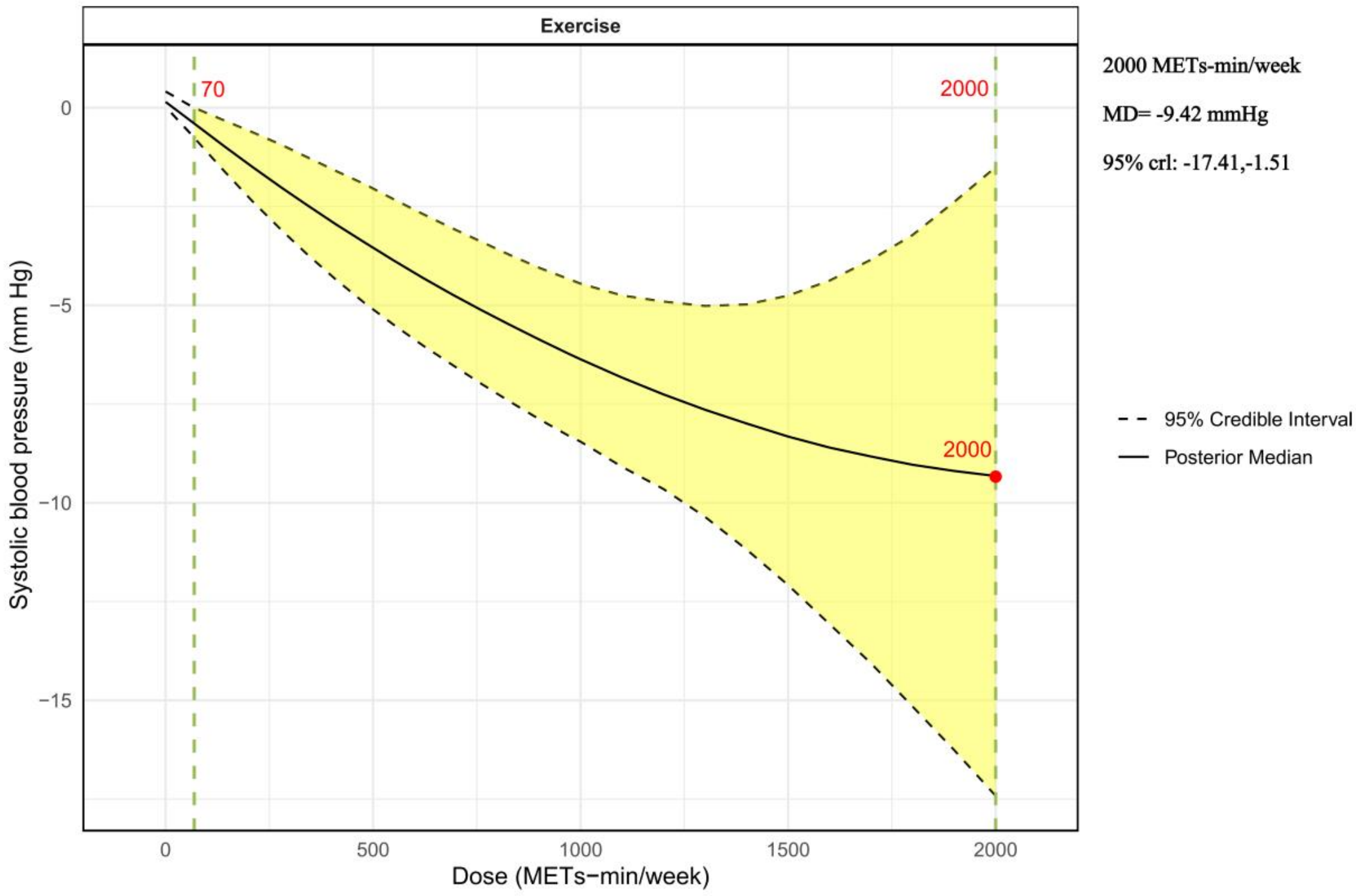

Figure S12. Dose–response relationship between exercise and systolic blood pressure (intervention length ≤12 weeks)

The green dashed lines represent the minimum and maximum effective doses (70 and 2000 METs-min/week), while the yellow shaded area indicates the significant region. and the red dots represent the optimal dose points (2000 METs-min/week).

7.5.5 Diastolic blood pressure (baseline blood pressure＞80 mmHg)

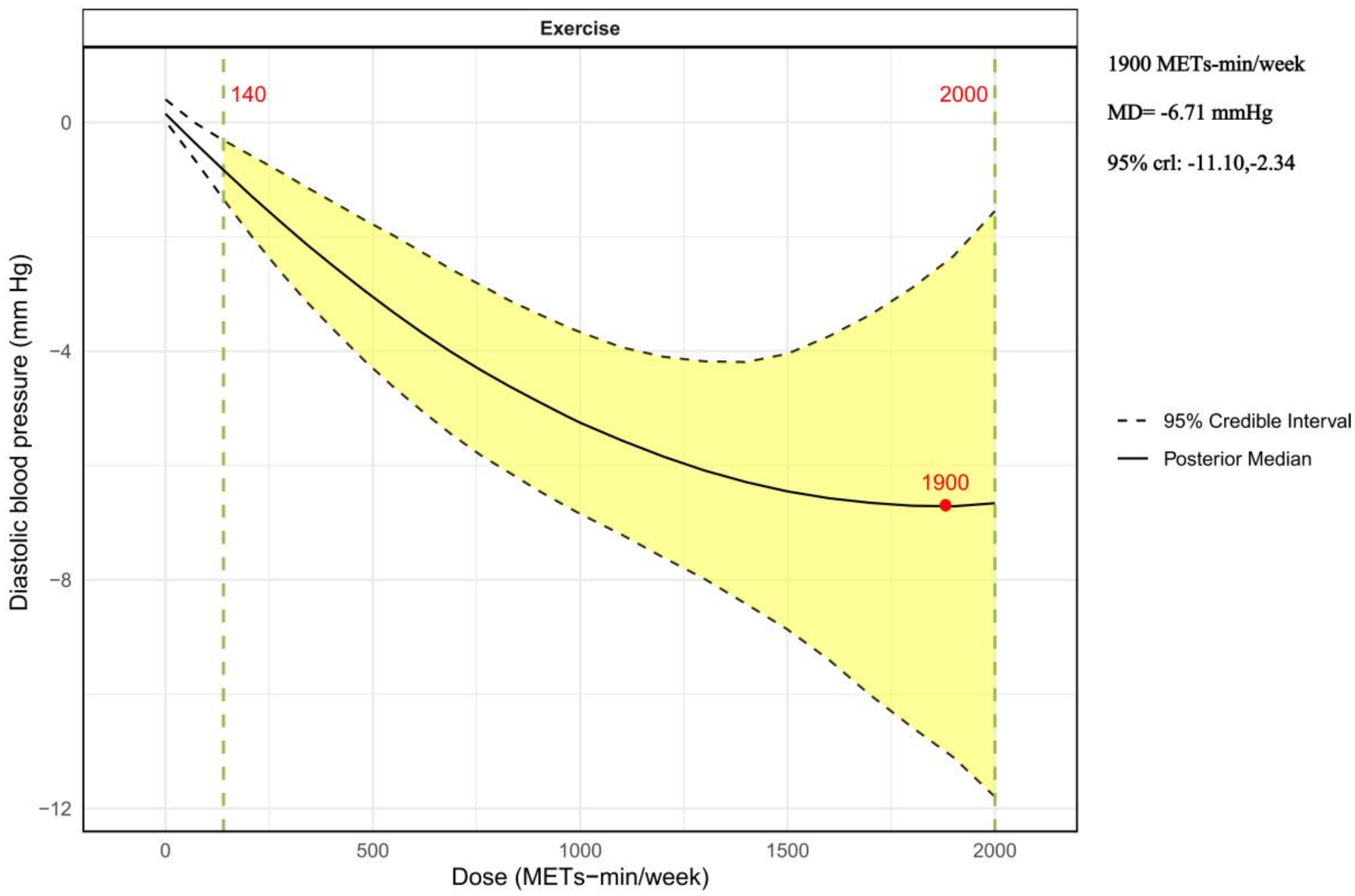

Figure S13. Dose–response relationship between exercise and diastolic blood pressure (baseline blood pressure＞80 mmHg)

The green dashed lines represent the minimum and maximum effective doses (140 and 2000 METs-min/week), while the yellow shaded area indicates the significant region. and the red dots represent the optimal dose points (1900 METs-min/week).

7.5.6 Diastolic blood pressure (baseline blood pressure≤80 mmHg)

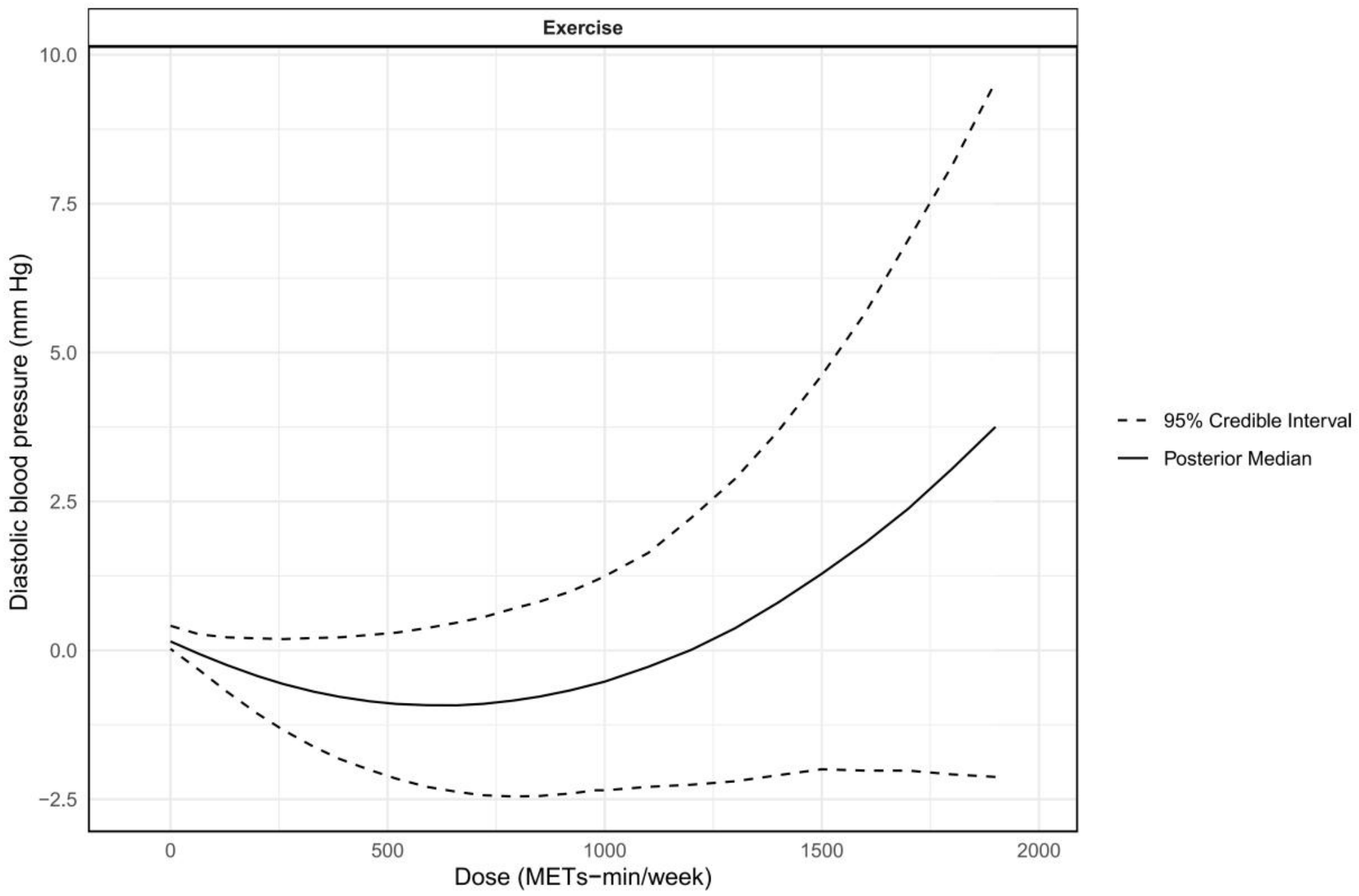

Figure S14 Dose–response relationship between exercise and diastolic blood pressure (baseline blood pressure≤80 mmHg).

7.5.7 Diastolic blood pressure (intervention length＞12 weeks)

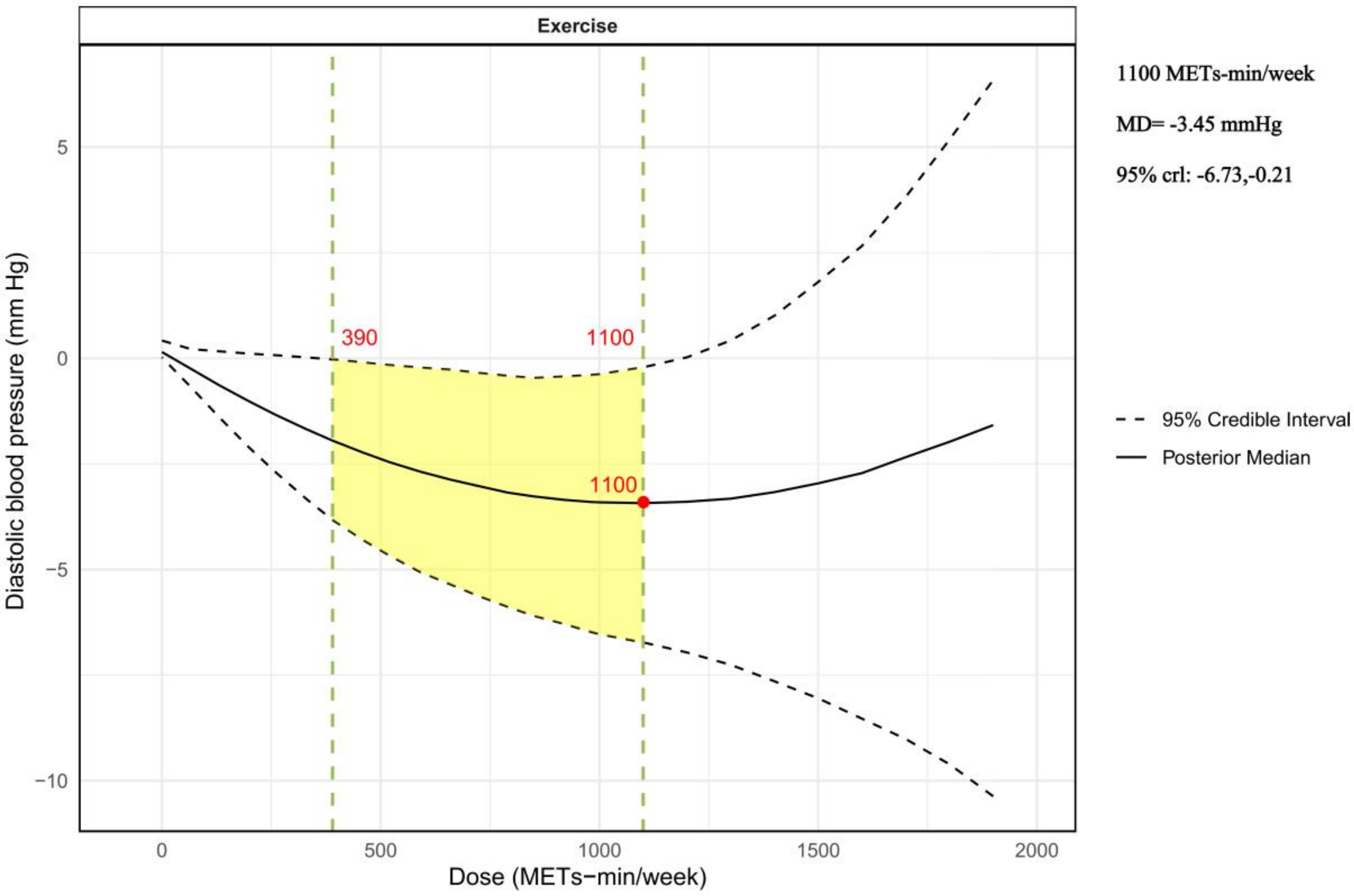

Figure S15. Dose–response relationship between exercise and diastolic blood pressure (intervention length＞12 weeks)

The green dashed lines represent the minimum and maximum effective doses (390 and 1100 METs-min/week), while the yellow shaded area indicates the significant region. and the red dots represent the optimal dose points (1100 METs-min/week).

7.5.8 Diastolic blood pressure (intervention length≤12 weeks)

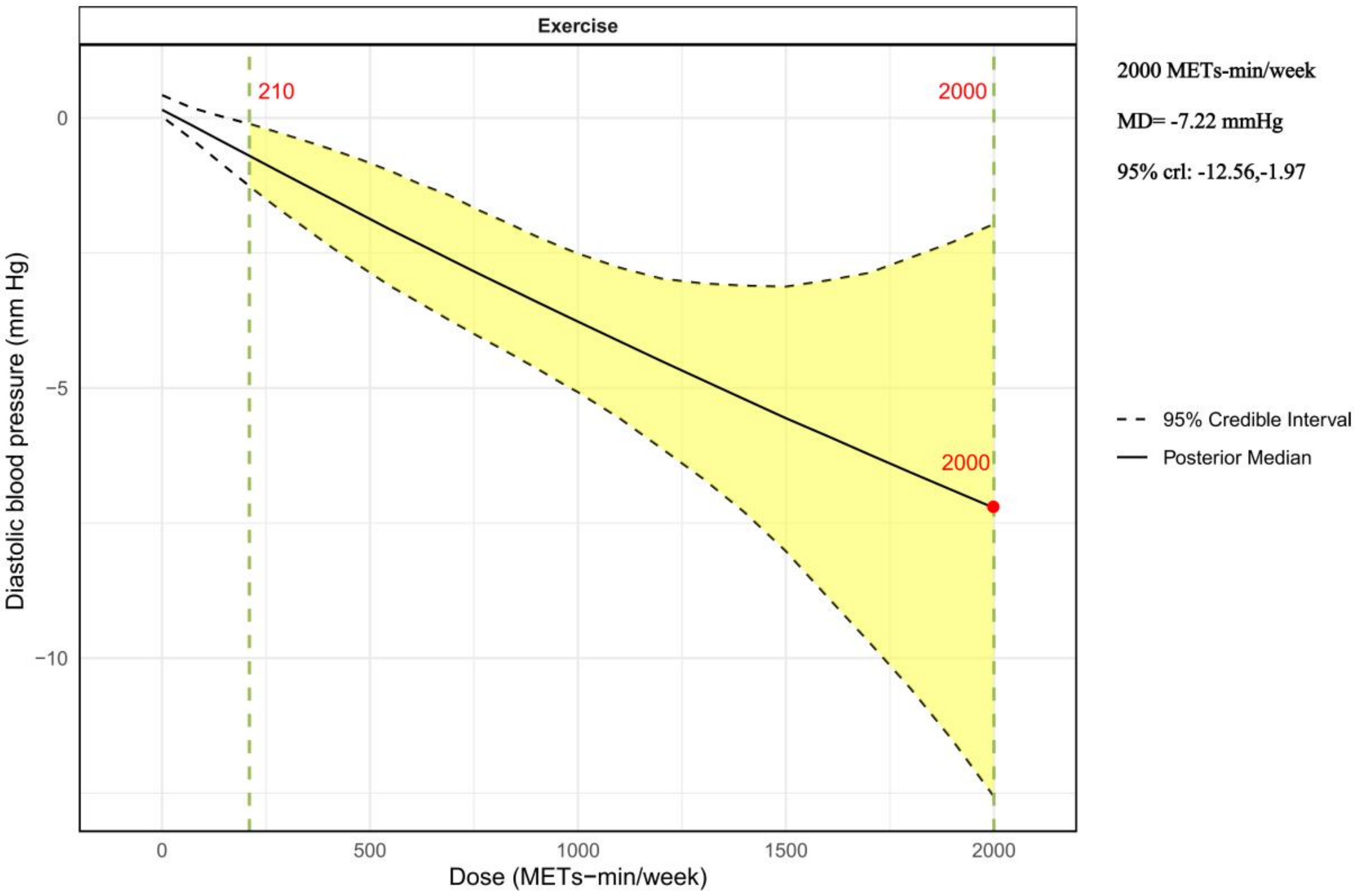

Figure S16. Dose–response relationship between exercise and diastolic blood pressure (intervention length≤12 weeks)

The green dashed lines represent the minimum and maximum effective doses (210 and 2000 METs-min/week), while the yellow shaded area indicates the significant region. and the red dots represent the optimal dose points (2000 METs-min/week).

7.6 Sensitivity analysis

7.6.1 Systolic blood pressure

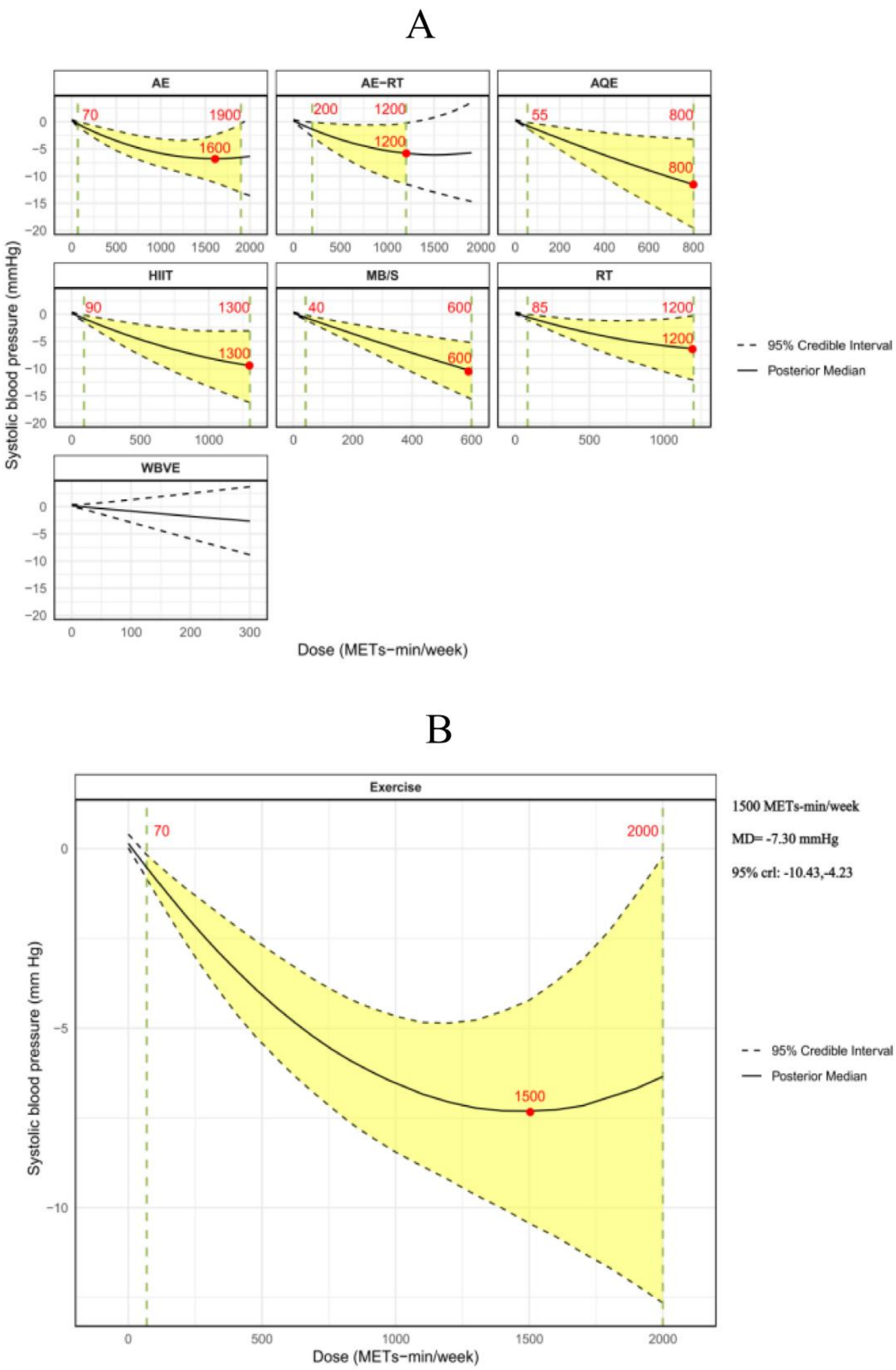

Figure S17. Sensitivity analysis of the relationship between exercise dose and systolic blood pressure after excluding high-risk studies, studies including perimenopausal participants, and studies with educational components in the control group. (A) Modality-specific dose-response relationships; (B) overall dose-response relationship.

7.6.2 Diastolic blood pressure

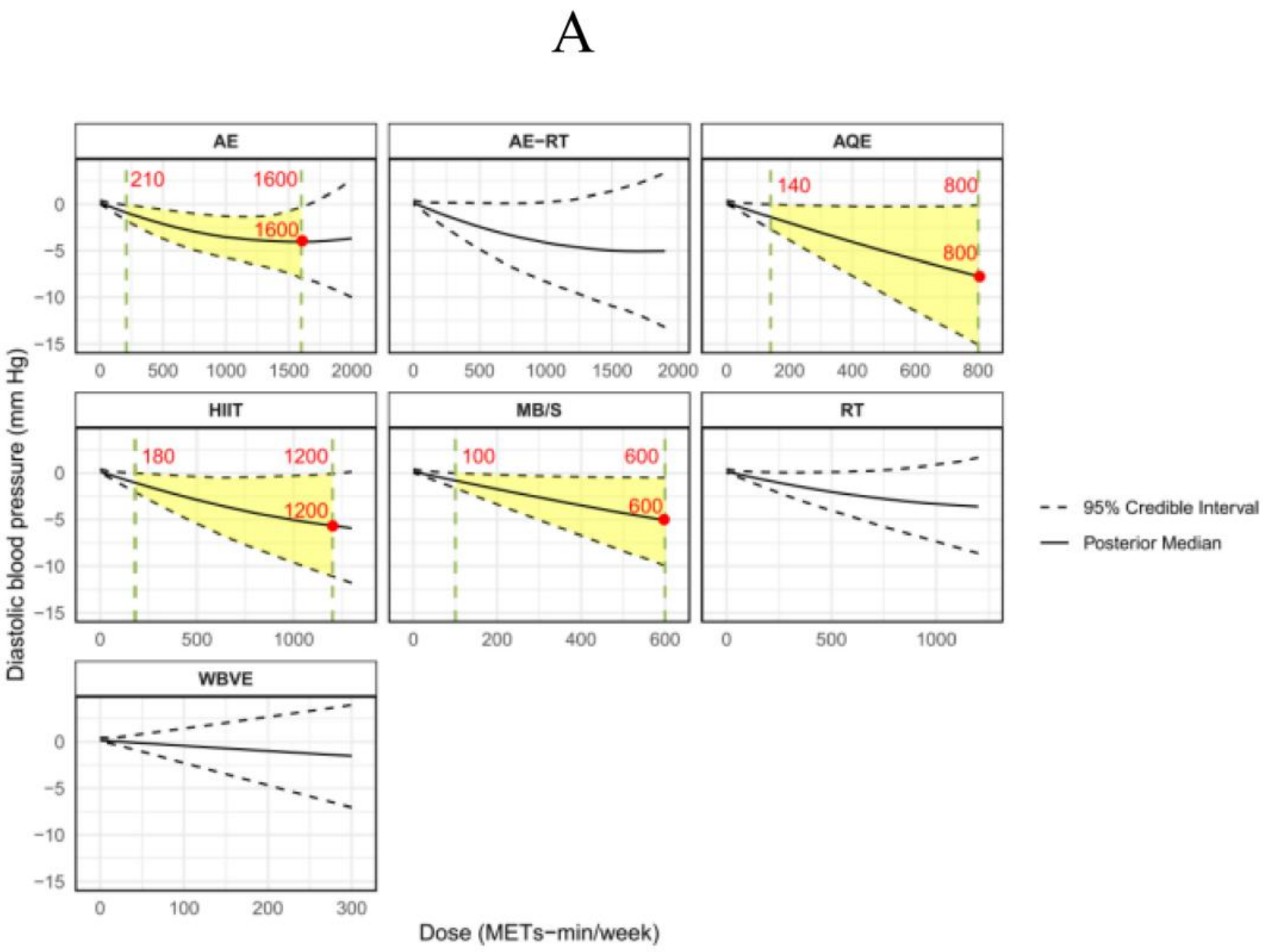

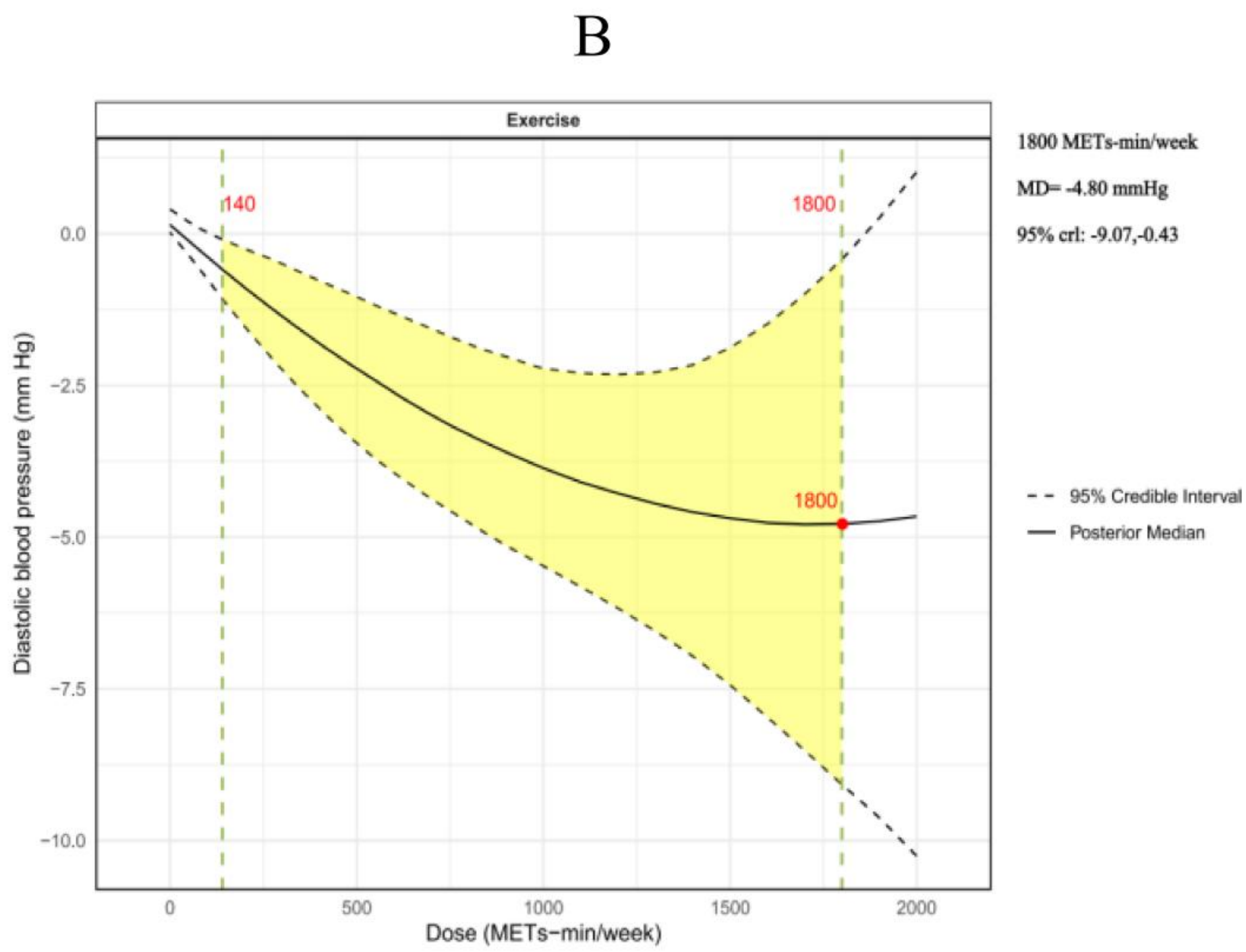

Figure S18. Sensitivity analysis of the relationship between exercise dose and diastolic blood pressure after excluding high-risk studies, studies including perimenopausal participants, and studies with educational components in the control group. (A) Modality-specific dose-response relationships; (B) overall dose-response relationship.

7.7 Effectiveness ranking of different exercises and doses

7.7.1 Table S10. Systolic blood pressure

Rank	Treatment	Mean	Median	2.50%	97.50%
1	AQE_800	12.04333333	3	1	79.025
2	AQE_770	13.894	5	2	81.05
3	AQE_740	15.90366667	6	3	85
4	MB/S_600	11.52333333	7	1	49.025
5	AQE_720	17.61566667	8	4	87
6	MB/S_580	13.57966667	10	2	53
7	AQE_690	19.875	11	5	90
8	HIIT_1300	19.966	12	1	89
9	MB/S_560	15.80433333	12	3	56.025
10	AQE_660	22.32766667	13	6	94
11	MB/S_540	18.14933333	14	4	60.025
12	HIIT_1200	22.642	15	2	90.025
13	AQE_630	25.003	16	7	97.025
14	MB/S_520	20.68466667	17	5	64
15	AQE_610	27.23866667	18	8	101
16	HIIT_1100	26.071	19	3	89
17	MB/S_500	23.347	19	6	68
18	AQE_580	30.342	21	9	103
19	MB/S_480	26.249	22	7	72.025
20	HIIT_1000	30.42733333	24	4	91
21	AQE_550	33.78966667	25	10	108
22	MB/S_460	29.348	25	8	77
23	HIIT_990	31.77933333	26	5	92.025
24	AQE_520	37.57566667	29	11	111.025
25	HIIT_940	34.90966667	29	6	95
26	MB/S_430	33.97333333	30	9	84
27	HIIT_900	37.83333333	32	7	97.025
28	AQE_500	40.67066667	33	12	114.025
29	MB/S_410	37.77066667	34	10	88
30	HIIT_850	41.58333333	37	8	101.025
31	AQE_470	45.14866667	38	13	119
32	MB/S_390	41.89533333	38	11	92.025
33	HIIT_810	45.041	40	11	104
34	RT_1200	52.629	41	2	168.05
35	MB/S_370	46.29533333	43	14	96.025
36	AQE_440	50.11333333	44	14	122
37	AE_1700	53.242	45	7	138
38	AE_1800	55.67333333	45	5	152
39	HIIT_760	49.48333333	45	14	108
40	AE_1600	51.819	46	10	125
41	AE_1900	58.95066667	46	3	171.025

42	RT_1100	55.23	46	5	157
43	AE_1500	51.38533333	47	13	113
44	MB/S_350	51.11933333	48	17	102
45	AE_1400	52.038	49	16	105
46	AE_2000	62.934	49	1	189.025
47	HIIT_720	53.56733333	49	17	111
48	AQE_410	55.61033333	50	16	126
49	AE_1300	53.81366667	52	19	100
50	RT_1000	58.996	52	10	149
51	MB/S_330	56.26433333	54	20.975	107
52	RT_990	60.134	54	11	147.05
53	AE_1200	56.64433333	55	22	98
54	AQE_390	59.96333333	55	18	129.025
55	HIIT_670	58.84666667	55	20	115.025
56	RT_950	62.52833333	57	14	145
57	AE_1100	60.62233333	60	26	100
58	HIIT_630	63.64333333	60	23	119
59	MB/S_310	61.87	60	24	111
60	RT_910	65.14733333	60	16	143
61	AQE_360	66.50733333	62	22	134
62	RT_870	68.038	64	19.975	139.025
63	AE_1000	65.69833333	66	31	104
64	AE-RT_1500	74.30066667	66	9	187
65	AE-RT_1600	75.70633333	66	6	188
66	MB/S_290	67.89233333	66	29	116
67	AE-RT_1400	73.722	67	11	184
68	AE-RT_1700	77.82866667	67	4	189
69	HIIT_580	69.95533333	67	27	123
70	AE_970	68.09833333	68	33	106
71	AE-RT_1300	74.09366667	68	14	175.025
72	RT_830	71.19633333	68	23	140
73	AE-RT_1800	80.63033333	69	2	190
74	AE-RT_1200	75.51433333	71	17	168
75	AQE_330	73.74766667	71	27	138
76	AE-RT_1900	84.11366667	72	1	191
77	RT_790	74.56033333	72	26.975	138
78	AE_900	72.79166667	73	37	110
79	MB/S_270	74.33066667	73	34.975	121
80	AE-RT_1100	77.986	74	21	162
81	HIIT_540	75.655	74	32	127
82	RT_740	78.84033333	77	31	138
83	AE_830	78.02566667	78	41	115
84	AE-RT_1000	81.641	79	25	158.025
85	AQE_300	81.59433333	80	33	142

86	MB/S_250	81.14733333	80	40	126
87	AE-RT_980	83.08033333	81	27	156.05
88	HIIT_490	83.09433333	82	39	133
89	RT_700	82.67	82	36	138
90	AE_760	83.76766667	84	46	120
91	AE-RT_920	86.281	85	30	157
92	AQE_280	87.45	86	38	146
93	RT_660	86.81433333	87	40	139.025
94	MB/S_230	88.348	88	47	131
95	HIIT_450	89.74166667	89	46	137
96	AE-RT_850	90.453	90	35	157
97	AE_690	90.01666667	91	52	125
98	RT_620	91.22866667	91	46	141.025
99	AE-RT_790	94.61633333	94	39	157
100	AQE_250	96.31666667	96	47	151
101	MB/S_210	95.798	96	54.975	137
102	RT_580	95.84333333	96	50	144
103	AE_620	96.89766667	98	60	132
104	HIIT_400	98.47133333	98	54	144
105	AE-RT_720	99.89633333	100	44	160
106	RT_540	100.6943333	101	56.975	147
107	MB/S_190	103.5993333	104	64	142
108	AE_550	104.3433333	105	67	138
109	AE-RT_660	105.013	105	50	161.025
110	AQE_220	105.729	106	57.975	157
111	RT_500	105.7913333	106	62	149
112	HIIT_360	106.01	106.5	63.975	148
113	AE-RT_590	111.2973333	111	59	164
114	MB/S_170	111.6536667	112	73	148
115	RT_460	111.056	112	68.975	152
116	AE_480	112.4313333	114	75	144
117	WBVE_300	114.2296667	115.5	17	191
118	AQE_190	115.6596667	116	70	161
119	HIIT_310	115.73	116	76.975	154
120	AE-RT_520	118.1313333	118	69	166
121	RT_410	117.6426667	118	78	155.025
122	WBVE_290	116.3693333	118	20	190
123	WBVE_280	118.564	121	23	189
124	AE_410	121.1843333	122	84	151
125	AQE_170	122.7416667	123	80	164
126	HIIT_270	123.906	124	86	159
127	MB/S_140	123.512	124	88.975	156
128	RT_370	123.332	124	87	159
129	WBVE_270	120.7696667	124	26.975	188

130	AE-RT_460	124.5346667	125	78.975	168
131	WBVE_260	123.0236667	126	30	187
132	RT_330	129.234	129	95	162
133	WBVE_250	125.3063333	129	33.975	186
134	AE_340	130.5513333	131	96.975	158
135	AE-RT_390	132.2543333	132	91	171
136	MB/S_120	132.004	132	99	162
137	WBVE_240	127.656	132	39	185
138	AQE_140	133.2086667	133	95	170
139	HIIT_220	134.279	134	100.975	165
140	WBVE_230	130	134.5	44	184
141	RT_290	135.377	135	103	166
142	WBVE_220	132.4393333	137	49	183
143	AE-RT_330	139.3966667	138	101	174
144	AE_280	139.3343333	139	108	165
145	MB/S_100	140.6093333	140	109	167
146	WBVE_210	134.923	140	53	182
147	RT_250	141.6626667	141	109.975	169
148	WBVE_200	137.4306667	142	58	181
149	HIIT_180	143.1823333	143	112	171
150	AQE_110	144.0283333	144	109	174
151	WBVE_190	140.008	145	64	180
152	AE-RT_260	148.151	147	115	178
153	MB/S_83	148.1913333	148	117	172
154	RT_210	148.1166667	148	117	173
155	WBVE_180	142.537	148	70.975	179
156	AE_210	150.0683333	150	121	172
157	WBVE_170	145.095	151	76	178
158	WBVE_160	147.6643333	153	82	177
159	AQE_83	154.0646667	155	123	179
160	HIIT_130	154.49	155	126	177
161	AE-RT_200	156.3146667	156	129	181
162	RT_170	154.8923333	156	127	177
163	WBVE_140	152.157	158	94.975	177
164	MB/S_62	157.544	159	129.975	178
165	WBVE_130	154.7646667	160	101	178
166	WBVE_120	157.3226667	163	108	179
167	AE_140	161.557	164	137	179
168	WBVE_110	159.9413333	165	115	180
169	HIIT_90	164.179	167	139	182
170	RT_120	163.546	167	139	182
171	AQE_55	164.7616667	168	138	184
172	WBVE_100	162.509	168	122	181
173	AE-RT_130	166.3156667	169	144	186

174	WBVE_72	169.8376667	170	141	184
175	WBVE_83	167.0433333	170	133	183
176	WBVE_93	164.5003333	170	126	182
177	MB/S_41	167.1893333	171	143	183
178	WBVE_62	172.4043333	173	146	185
179	RT_83	170.5336667	175	148	186
180	WBVE_52	174.943	177	152	186
181	AE_69	174.172	179	151	187
182	WBVE_41	177.6006667	180	158.975	187
183	AE-RT_66	176.4086667	181	154	189
184	AQE_28	175.75	181	151	189
185	HIIT_45	175.6386667	181	152	189
186	MB/S_21	177.2283333	183	154	189

Note: The number that follows the exercise intervention indicates the dose of exercise (METs-min/week).AE: Aerobic Exercise; AE-RT: Combined Aerobic and Resistance Training; RT: Resistance Training;MB/S:Mind-body exercise or stretching;HIIT: High-Intensity Interval Training;AQE:Aquatic Exercise; WBVE:Whole-Body Vibration Exercise;CG: Control group;AQE rankings should be interpreted cautiously because they were based on only three intervention arms and wide credible intervals.

7.7.2 Table S11. Diastolic blood pressure

Rank	Treatment	Mean	Median	2.50%	97.50%
1	AQE_800	16.373	3	1	130.025
2	AQE_770	18.057	4	2	131
3	AQE_740	19.86	6	3	129.025
4	AQE_720	21.436	8	4	130
5	AQE_690	23.459	11	5	128.075
6	AQE_660	25.598	13	6	129
7	AQE_630	27.94866667	15	7	130
8	AQE_610	29.93433333	17	8	130.05
9	HIIT_1300	31.11933333	17	1	149
10	AQE_580	32.562	20	9	132.025
11	HIIT_1200	32.96633333	20	2	141
12	AQE_550	35.48433333	23	10	132.025
13	HIIT_1100	35.57766667	24	3	137
14	MB/S_600	33.59733333	24	1	127.025
15	AE-RT_1800	51.305	26	2	190
16	AE-RT_1900	53.45566667	26	1	191
17	AE-RT_1700	49.67533333	27	3	189
18	AQE_520	38.646	27	11	135
19	MB/S_580	36.026	27	2	128
20	AE-RT_1600	48.71133333	28	4	188
21	AE-RT_1500	48.37966667	29	5	187
22	HIIT_1000	39.04733333	29	4	132

23	AQE_500	41.282	30	12	136.025
24	MB/S_560	38.55233333	30	3	128.025
25	HIIT_990	40.24566667	31	5	132.025
26	AE-RT_1400	48.69666667	32	6	185
27	MB/S_540	41.218	33	4	130
28	AE-RT_1300	49.73366667	34	7	180
29	AQE_470	44.981	34	13	137.025
30	HIIT_940	42.88333333	34	6	133
31	MB/S_520	44.011	36	5	132.025
32	HIIT_900	45.38566667	37	7	133.025
33	AE-RT_1200	51.63833333	38	8	175.025
34	AQE_440	49.07833333	39	14	140
35	MB/S_500	46.93166667	39	6	134
36	HIIT_850	48.54266667	41	8	133
37	AE-RT_1100	54.37566667	42	9	170
38	MB/S_480	49.95766667	42	7	137
39	HIIT_810	51.529	44	9	134
40	AQE_410	53.60866667	45	15	142
41	MB/S_460	53.182	46	8	137
42	AE-RT_1000	58.06533333	48	10	167.025
43	AE-RT_980	59.586	49	11	166.025
44	AQE_390	57.221	49	16	145
45	HIIT_760	55.28966667	49	10	135.025
46	MB/S_430	57.78666667	51	11	139
47	RT_1200	66.63733333	51	1	191
48	HIIT_720	58.83033333	53	12.975	136.025
49	AE-RT_920	62.75533333	54	12	166
50	AQE_360	62.62733333	55	17	146
51	RT_1100	67.94666667	55	4	190
52	AE_1500	63.372	56	12	152
53	AE_1600	65.112	56	9	166.025
54	AE_1700	67.74633333	56	6	184
55	MB/S_410	61.40866667	56	14	141
56	AE_1400	62.683	58	14	139
57	AE_1800	71.09833333	58	4	189
58	HIIT_670	63.34733333	58	16	139.025
59	AE_1900	75.00933333	59	2	190
60	AE-RT_850	66.81066667	59	14	166
61	AE_1300	63.05733333	60	17	128.025
62	MB/S_390	65.21266667	60	17	143
63	RT_1000	70.22766667	60	7	189
64	AE_1200	64.51666667	62	20	123
65	AE_2000	79.34366667	62	1	191
66	AQE_330	68.65266667	62	19	149

67	RT_990	71.032	62	8.975	188
68	HIIT_630	67.47766667	63	19	142
69	AE-RT_790	70.896	64	17	165.05
70	MB/S_370	69.23466667	65	20	145.025
71	RT_950	72.67233333	65	11	187
72	AE_1100	67.145	66	23	121.025
73	RT_910	74.54866667	68	13	185
74	HIIT_580	72.913	69	23	145
75	AQE_300	75.41466667	70	24.975	152
76	MB/S_350	73.47	70	23	146.025
77	AE_1000	70.98966667	71	27	121
78	AE-RT_720	76.09	71	21	166
79	RT_870	76.651	71	15	183
80	AE_970	72.93033333	73	29	123
81	RT_830	79.01333333	74.5	17	180
82	HIIT_540	77.84466667	75	27	148
83	MB/S_330	77.90333333	75	27	149
84	AE_900	76.874	76	32.975	127
85	AE-RT_660	81.27866667	77	24	167
86	AQE_280	80.735	77	28	154
87	RT_790	81.60466667	78	20	178
88	MB/S_310	82.58933333	80	30	151
89	AE_830	81.42866667	81	37	131
90	HIIT_490	84.31966667	82	32	151.025
91	RT_740	84.997	82	23.975	175
92	AE-RT_590	87.88266667	85	29	168
93	AE_760	86.48833333	86	41.975	136
94	AQE_250	88.789	86	35	158
95	MB/S_290	87.483	86	34	153
96	RT_700	88.09033333	86	27	172.025
97	HIIT_450	90.16233333	89	37	154.025
98	RT_660	91.47933333	90	30	170
99	MB/S_270	92.68133333	91	39	156
100	AE_690	92.125	92	46	141
101	AE-RT_520	95.40433333	94	36	170
102	RT_620	95.09666667	94	35	169
103	AQE_220	97.608	96	43	162
104	HIIT_400	97.81166667	97	45	159
105	MB/S_250	98.12633333	97	45	159
106	AE_620	98.28266667	98	52	145
107	RT_580	98.956	98.5	39	167
108	AE-RT_460	102.7593333	103	43	172
109	RT_540	103.1203333	103	45	166
110	HIIT_360	104.6536667	104	52	161

111	MB/S_230	103.89	104	51	161.025
112	AE_550	105.0313333	105	59	150
113	AQE_190	107.1783333	106	54	165
114	RT_500	107.5263333	108	51	167
115	MB/S_210	109.7923333	110	58	164
116	WBVE_300	111.235	112	8	191
117	RT_460	112.1513333	112.5	57	167.025
118	AE_480	112.4133333	113	69	155
119	AE-RT_390	112.215	113	54.975	172
120	HIIT_310	113.548	113	62.975	165
121	AQE_170	114.171	114	62	168
122	WBVE_290	112.995	114	11	190
123	MB/S_190	115.973	116	65.975	166
124	WBVE_280	114.8283333	116	13	189
125	RT_410	118.1566667	119	66	167.025
126	WBVE_270	116.705	119	15.975	188
127	AE_410	120.417	122	77	160
128	AE-RT_330	121.1546667	122	65	172.025
129	HIIT_270	121.3933333	122	74	168.025
130	WBVE_260	118.617	122	18	187
131	MB/S_170	122.225	123	73	169
132	RT_370	123.359	124	74	168
133	AQE_140	124.856	125	76	170.025
134	WBVE_250	120.6396667	125	20	186
135	WBVE_240	122.6443333	128	23	185
136	RT_330	128.8263333	129	82	169.025
137	AE_340	129.062	130	86	166
138	WBVE_230	124.7283333	130	27	184
139	HIIT_220	131.379	132	87	171
140	MB/S_140	131.4413333	133	85.975	172
141	WBVE_220	126.879	133	31	183
142	AE-RT_260	132.4643333	134	83	176
143	RT_290	134.5006667	135	91	171
144	WBVE_210	129.056	135.5	35	182
145	AQE_110	136.1016667	137	91	175
146	AE_280	137.3303333	138	97.975	171
147	WBVE_200	131.3163333	138	39	181
148	HIIT_180	139.8446667	140	99	175
149	MB/S_120	138.1813333	140	94.975	175
150	RT_250	140.3853333	141	100	173
151	WBVE_190	133.6473333	141	43	180
152	AE-RT_200	142.979	144	99	180
153	WBVE_180	136.0016667	144	48	179
154	MB/S_100	145.0583333	146	104	178

155	AQE_83	146.7953333	147	105.975	180
156	RT_210	146.498	147	109	176
157	WBVE_170	138.4273333	147	53	178
158	AE_210	147.5023333	148	112	177
159	WBVE_160	140.9036667	150	60	177
160	HIIT_130	150.7206667	151	112	180
161	MB/S_83	151.153	151	111	181
162	RT_170	152.8506667	153	117	179
163	WBVE_140	145.481	154	72	176
164	AE-RT_130	155.8806667	157	117	184
165	WBVE_130	148.0496667	157	78.975	177
166	AQE_55	158.302	159	121	185
167	WBVE_120	150.674	159	85.975	178
168	AE_140	158.3266667	160	122	183
169	MB/S_62	158.755	160	121	184
170	HIIT_90	160.0246667	162	123	184
171	WBVE_110	153.2986667	162	92	180
172	RT_120	160.9093333	163	127	183
173	WBVE_100	155.922	164	98.975	181
174	WBVE_93	157.9606667	166	103.975	182
175	WBVE_52	168.9956667	168	135	186
176	WBVE_62	166.3046667	168	128	185
177	WBVE_83	160.668	168	111	183
178	WBVE_72	163.5916667	169	120	184
179	MB/S_41	166.575	171	129	187.025
180	RT_83	167.4436667	172	135	187
181	AE-RT_66	168.645	174	131	189
182	WBVE_41	171.9936667	174	142	187
183	AQE_28	170.0553333	175	133	189
184	AE_69	170.399	177	133	188
185	HIIT_45	171.1576667	177	133	189
186	WBVE_31	174.7293333	178	148	188

Note: The number that follows the exercise intervention indicates the dose of exercise (METs-min/week).AE: Aerobic Exercise; AE-RT: Combined Aerobic and Resistance Training; RT: Resistance Training;MB/S:Mind-body exercise or stretching;HIIT: High-Intensity Interval Training;AQE:Aquatic Exercise; WBVE:Whole-Body Vibration Exercise;CG: Control group;AQE rankings should be interpreted cautiously because they were based on only three intervention arms and wide credible intervals.

7.8 Meta-regression

7.8.1 Systolic blood pressure

Table S12. Dose-response network meta-regression(systolic blood pressure)

Covariate	Systolic blood pressure			
	DIC	ResidualDeviance	Shared beta (median and 95%CrI)	SD
Age (years)	1155.9	575.8	-0.06 (-0.46, 0.33)	1.08 (0.77, 1.52)
Baseline blood pressure (mm Hg)	1147.1	564.4	-0.08 (-0.26, 0.12)	0.55 (0.41, 0.78)
Intervention length (weeks)	1070.5	490.8	-0.03 (-0.58, 0.51)	1.65(1.27, 2.27)

7.8.2 Diastolic blood pressure

Table S13. Dose response network meta-regression (diastolic blood pressure)

Covariate	Diastolic blood pressure			
	DIC	ResidualDeviance	Shared beta (median and 95%CrI)	SD
Age (years)	1013.1	579.5	-0.02 (-0.50, 0.42)	1.46 (1.09, 1.96)
Baseline blood pressure (mm Hg)	924	508.9	-0.18 (-0.93, 0.54)	2.56 (2.01, 3.28)
Intervention length (weeks)	1036.4	593.5	0.02 (-0.56, 0.65)	1.89 (1.41, 2.68)

8. Publication bias

8.1 Systolic blood pressure

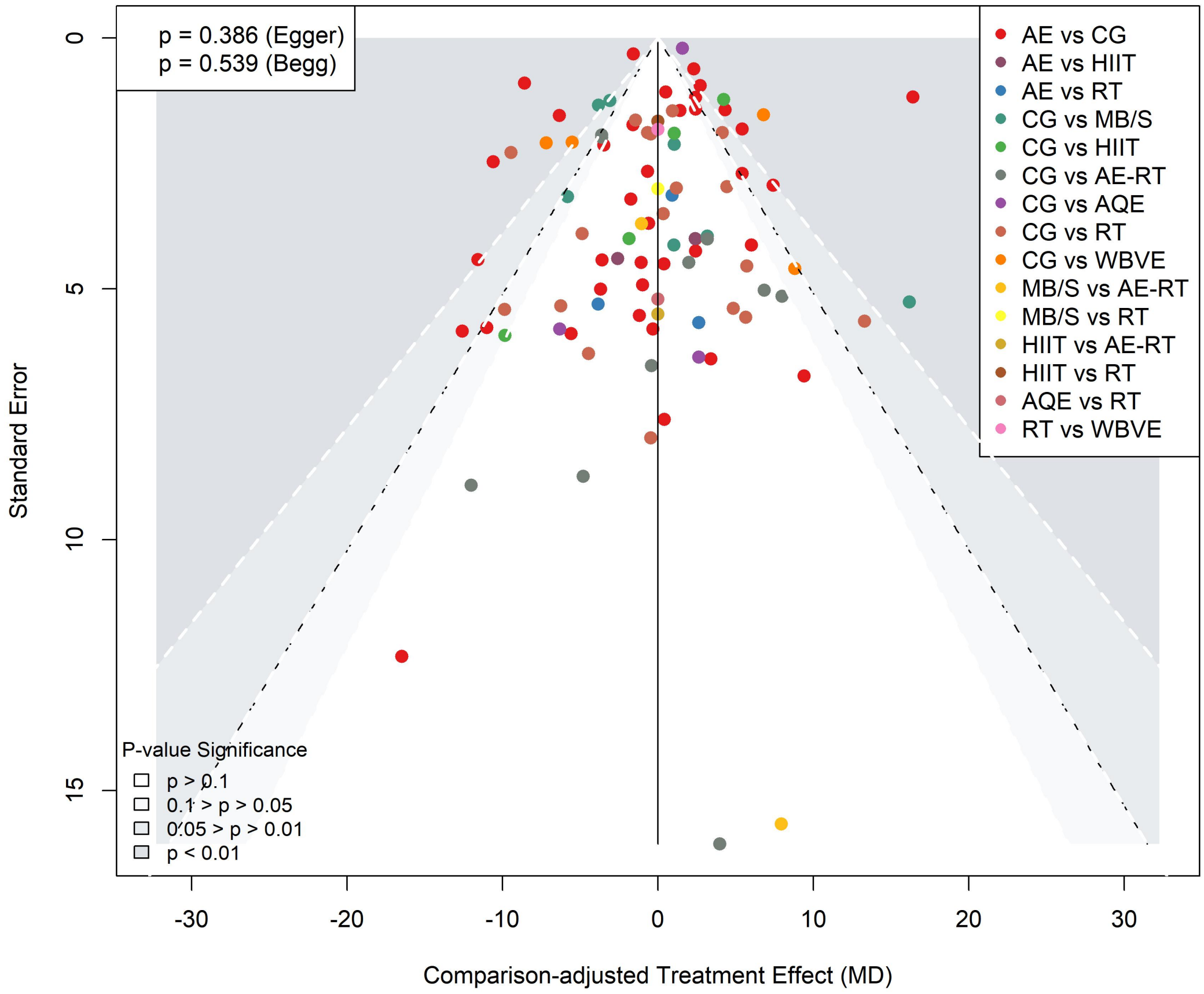

Figure S19. Funnel plot (systolic blood pressure).

AE: Aerobic Exercise; AE-RT: Combined Aerobic and Resistance Training; RT: Resistance Training;MB/S:Mind-body exercise or stretching;HIIT: High-Intensity Interval Training;AQE:Aquatic Exercise; WBVE:Whole-Body Vibration Exercise;CG: Control group

8.2 Diastolic blood pressure

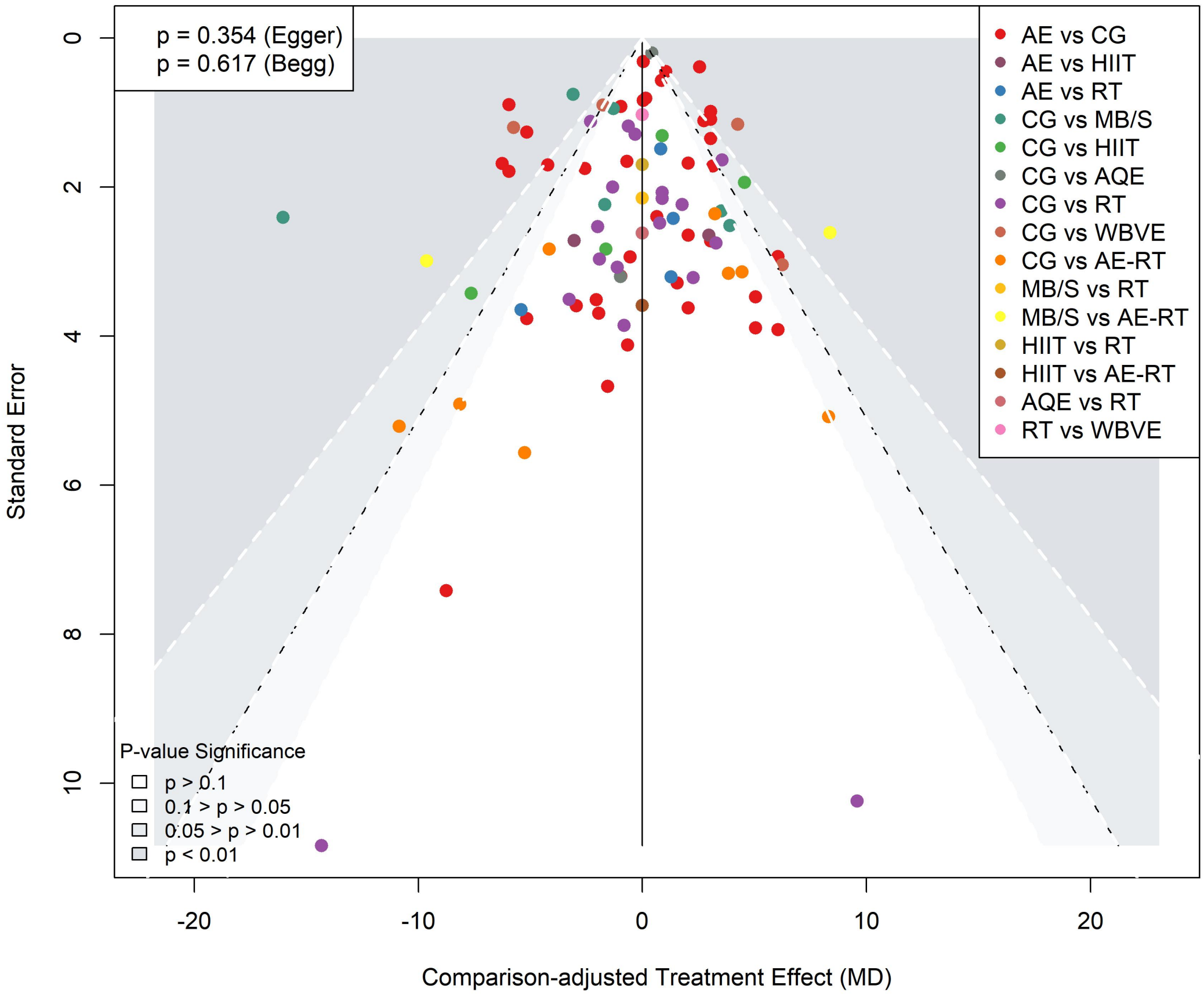

Figure S20. Funnel plot (diastolic blood pressure).

AE: Aerobic Exercise; AE-RT: Combined Aerobic and Resistance Training; RT: Resistance Training;MB/S:Mind-body exercise or stretching;HIIT: High-Intensity Interval Training;AQE:Aquatic Exercise; WBVE:Whole-Body Vibration Exercise;CG: Control group

9. Grade summary of all studies

Based on the CINeMA online guidelines (https://cinema.ispm.unibe.ch/), we evaluated blood pressure only. Downgrading decisions were made based on the following criteria:

9.1 Within-study Bias

Each included study was rated for risk of bias as low (RoB2 1 point), moderate (RoB2 2 points), or high (RoB2 3 points). The rule applied was the average RoB. No downgrade occurred for “no concerns”; one level was downgraded for “some concerns,” and two levels for “major concerns.”

9.2 Reporting Bias (Publication Bias and Selective Reporting)

To minimize publication bias, we examined the reference lists of recent relevant studies. Publication bias was assessed using comparison-adjusted funnel plots, Begg's and Egger’s test (Supplementary Content 8). The results indicated no statistically significant bias ($p > 0.05$), suggesting a low risk of publication bias. Furthermore, no high risk of selective reporting was identified; therefore, this domain was not downgraded.

9.3 Indirectness

Indirectness assessment was conducted by analyzing the clinical and methodological

relevance of the included studies to the research question. All studies met the PICOS criteria: they involved peri- and postmenopausal women , employed various exercise interventions, established appropriate control groups, and used blood pressure as the outcome measure. Although differences in baseline menopausal stage and hormonal status across studies may introduce some indirectness, this was addressed through sensitivity analyses excluding perimenopausal participants.

9.4 Imprecision

In the results of this pooled network analysis, blood pressure was treated as a continuous variable. Since blood pressure measurements were standardized across studies, mean difference was used as the effect size (change score: endpoint value minus baseline value). Given the absence of a clearly defined minimum clinically important difference (CID) for blood pressure changes in perimenopausal and postmenopausal populations, zero values (0) were adopted as the reference threshold for interpreting blood pressure changes.

9.5 Heterogeneity

In addition to statistical heterogeneity, clinical heterogeneity arising from differences in baseline menopausal stage, baseline blood pressure, comorbidity profile, and exercise supervision across included studies was considered when assigning heterogeneity ratings. Comparisons with prediction intervals that extended substantially beyond the credible interval in clinical terms were downgraded accordingly, as detailed in the individual comparison assessments in Tables S14 and S15.

9.6 Incoherence

Incoherence was addressed by p-value thresholds: no downgrade for $p > 0.1$, one level for p 0.05–0.1, and two levels for $p < 0.05$.

9.7 Summarizing Judgments Across CINeMA Domains

The CINeMA framework assesses confidence in evidence across six domains: within-study bias, reporting bias, indirectness, imprecision, heterogeneity, and incoherence. These judgments are summarized into an overall confidence rating using the GRADE approach (high, moderate, low, or very low). As a single issue may impact multiple domains (e.g., indirectness due to intransitivity or heterogeneity affecting both imprecision and bias), all domains are considered together to avoid redundant downgrading and ensure a balanced assessment.

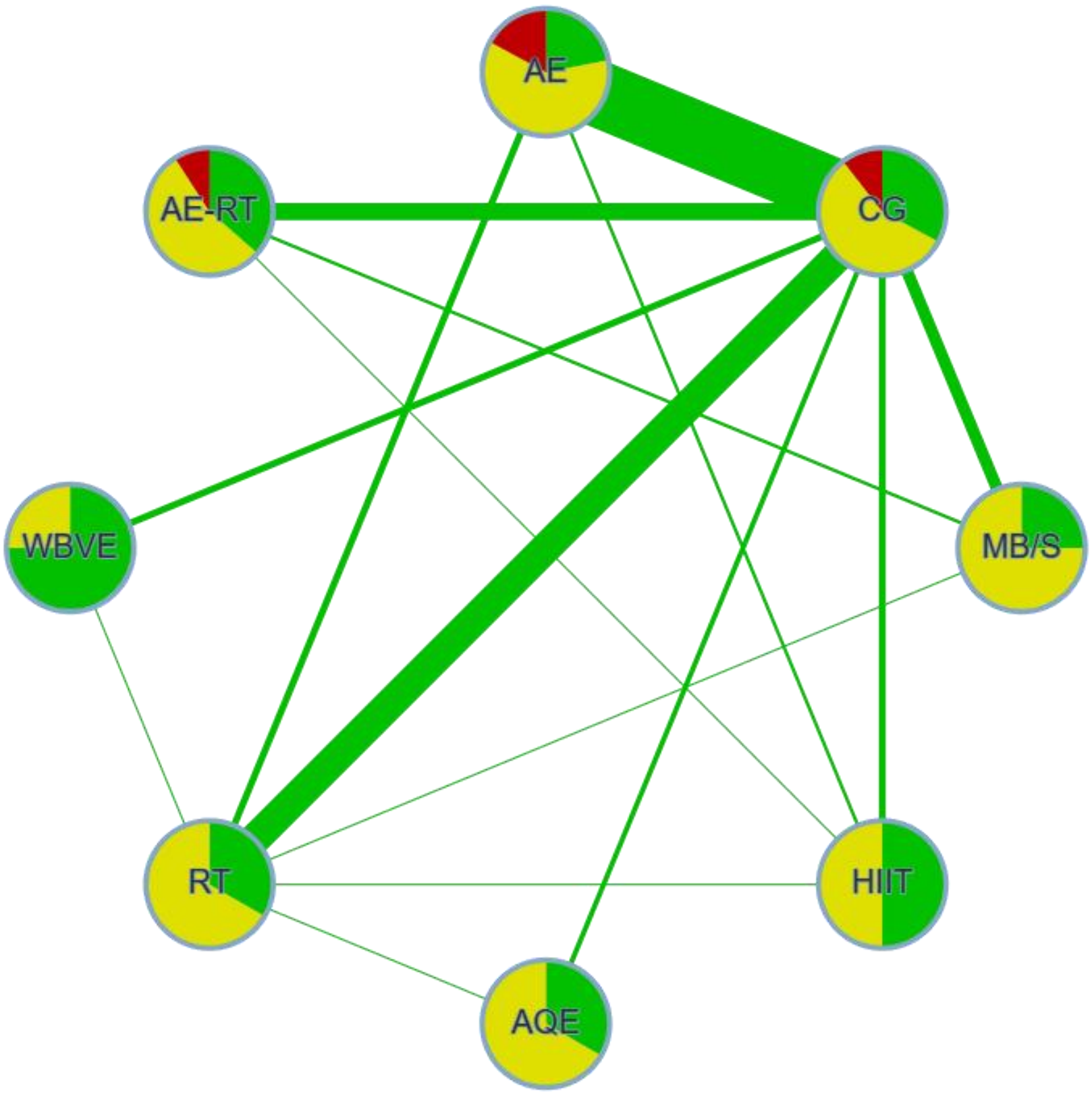

Figure S21. Network plot of study limitations of the included studies (systolic blood pressure).

The colors in the circles indicate the percentage of low RoB studies (green), moderate RoB studies (yellow), high RoB studies (red) about each physical activity type. Edge width by the number of studies. Edge color by average RoB. The colors of the lines indicate the summative RoB assessment of each comparison. Low RoB is green, moderate RoB is yellow, high RoB is red.AE: Aerobic Exercise; AE-RT: Combined Aerobic and Resistance Training; RT: Resistance Training;MB/S:Mind-body exercise or stretching;HIIT: High-Intensity Interval Training;AQE:Aquatic Exercise; WBVE:Whole-Body Vibration Exercise;CG: Control group

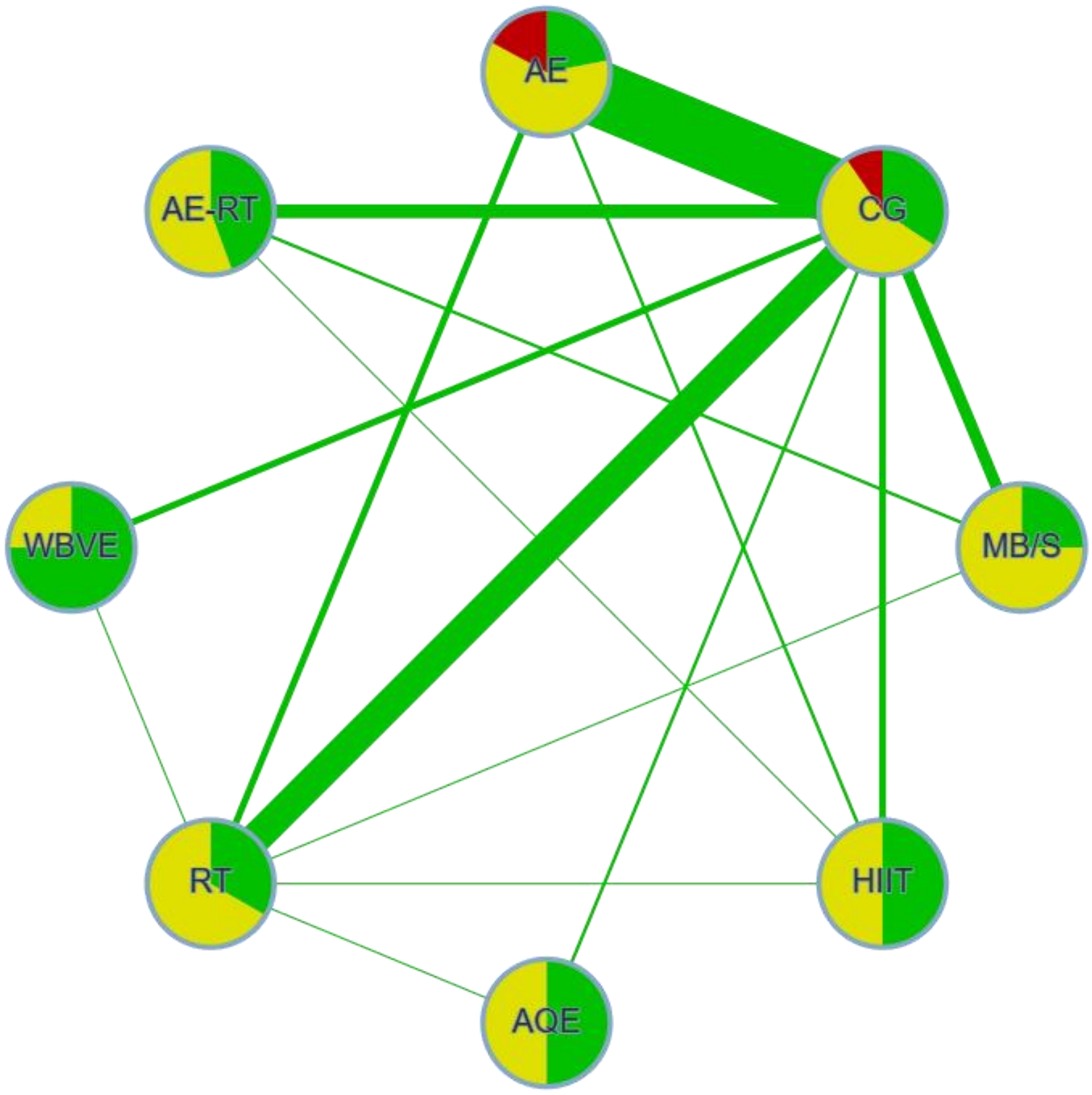

Figure S22. Network plot of study limitations of the included studies (diastolic blood pressure).

The colors in the circles indicate the percentage of low RoB studies (green), moderate RoB studies (yellow), high RoB studies (red) about each physical activity type. Edge width by the number of studies. Edge color by average RoB. The colors of the lines indicate the summative RoB assessment of each comparison. Low RoB is green, moderate RoB is yellow, high RoB is red.AE: Aerobic Exercise; AE-RT: Combined Aerobic and Resistance Training; RT: Resistance Training;MB/S:Mind-body exercise or stretching;HIIT: High-Intensity Interval Training;AQE:Aquatic Exercise; WBVE:Whole-Body Vibration Exercise;CG: Control group

Table S14. The confidence of evidence using the CINeMA (systolic blood pressure).

Comparison	Within-study bias	Reporting bias	Indirectness	Imprecision	Heterogeneity	Incoherence	Confidence rating	Reason(s) for downgrading
**AE:CG**	**Some concerns**	**Low risk**	**No concerns**	**No concerns**	**Major concerns**	**No concerns**	**Very low**	**["Within-study bias","Heterogeneity"]**
AE:HIIT	Some concerns	Low risk	No concerns	Major concerns	No concerns	No concerns	Very low	["Within-study bias","Imprecision"]
AE:RT	Some concerns	Low risk	No concerns	Major concerns	No concerns	No concerns	Very low	["Within-study bias","Imprecision"]
**AE-RT:CG**	**Some concerns**	**Low risk**	**No concerns**	**No concerns**	**Major concerns**	**No concerns**	**Very low**	**["Within-study bias","Heterogeneity"]**
AE-RT:HIIT	Some concerns	Low risk	No concerns	Major concerns	No concerns	No concerns	Very low	["Within-study bias","Imprecision"]
AE-RT:MB/S	Some concerns	Low risk	No concerns	Major concerns	No concerns	No concerns	Very low	["Within-study bias","Imprecision"]
**AQE:CG**	**No concerns**	**Low risk**	**No concerns**	**No concerns**	**Major concerns**	**No concerns**	**Low**	**["Heterogeneity"]**
**AQE:RT**	**Some concerns**	**Low risk**	**No concerns**	**Major concerns**	**No concerns**	**No concerns**	**Very low**	**["Within-study bias","Imprecision"]**
**HIIT:CG**	**Some concerns**	**Low risk**	**No concerns**	**No concerns**	**Major concerns**	**No concerns**	**Very low**	**["Within-study bias","Heterogeneity"]**
**MB/S:CG**	**Some concerns**	**Low risk**	**No concerns**	**No concerns**	**Major concerns**	**Major concerns**	**Very low**	**["Within-study bias","Heterogeneity","Incoherence"]**
**RT:CG**	**Some concerns**	**Low risk**	**No concerns**	**No concerns**	**Major concerns**	**No concerns**	**Very low**	**["Within-study bias","Heterogeneity"]**
**WBVE:CG**	**No concerns**	**Low risk**	**No concerns**	**Major concerns**	**No concerns**	**No concerns**	**Low**	**["Imprecision"]**
HIIT:RT	Some concerns	Low risk	No concerns	Major concerns	No concerns	No concerns	Very low	["Within-study bias","Imprecision"]
MB/S:RT	Some concerns	Low risk	No concerns	Major concerns	No concerns	Major concerns	Very low	["Within-study bias","Imprecision","Incohere

Comparison	Within-study bias	Reporting bias	Indirectness	Imprecision	Heterogeneity	Incoherence	Confidence rating	Reason(s) for downgrading
								nce"]
RT:WBVE	Some concerns	Low risk	No concerns	Major concerns	No concerns	No concerns	Very low	["Within-study bias","Imprecision"]

AE: Aerobic Exercise; AE-RT: Combined Aerobic and Resistance Training; RT: Resistance Training;MB/S:Mind-body exercise or stretching;HIIT: High-Intensity Interval Training;AQE:Aquatic Exercise; WBVE:Whole-Body Vibration Exercise;CG: Control group

Table S15. The confidence of evidence using the CINeMA (diastolic blood pressure).

Comparison	Within-study bias	Reporting bias	Indirectness	Imprecision	Heterogeneity	Incoherence	Confidence rating	Reason(s) for downgrading
**AE:CG**	**Some concerns**	**Low risk**	**No concerns**	**No concerns**	**Major concerns**	**No concerns**	**Very low**	**["Within-study bias","Heterogeneity"]**
AE:HIIT	Some concerns	Low risk	No concerns	Major concerns	No concerns	No concerns	Very low	["Within-study bias","Imprecision"]
AE:RT	Some concerns	Low risk	No concerns	Major concerns	No concerns	No concerns	Very low	["Within-study bias","Imprecision"]
**AE-RT:CG**	**Some concerns**	**Low risk**	**No concerns**	**No concerns**	**Major concerns**	**Major concerns**	**Very low**	**["Within-study bias","Heterogeneity","Incoherence"]**
AE-RT:HIIT	Some concerns	Low risk	No concerns	Major concerns	No concerns	No concerns	Very low	["Within-study bias","Imprecision"]
AE-RT:MB/S	Some concerns	Low risk	No concerns	Major concerns	No concerns	Major concerns	Very low	["Within-study bias","Imprecision","Incoherence"]
**AQE:CG**	**No concerns**	**Low risk**	**No concerns**	**Major concerns**	**Major concerns**	**No concerns**	**Very low**	**["Imprecision","Heterogeneity"]**
**AQE:RT**	**Some concerns**	**Low risk**	**No concerns**	**No concerns**	**Major concerns**	**No concerns**	**Very low**	**["Within-study bias","Heterogeneity"]**
**HIIT:CG**	**Some concerns**	**Low risk**	**No concerns**	**No**	**Major concerns**	**No**	**Very low**	**["Within-study**

Comparison	Within-study bias	Reporting bias	Indirectness	Imprecision	Heterogeneity	Incoherence	Confidence rating	Reason(s) for downgrading
				**concerns**		**concerns**		**bias","Heterogeneity"]**
**MB/S:CG**	**Some concerns**	**Low risk**	**No concerns**	**No concerns**	**Major concerns**	**No concerns**	**Very low**	**["Within-study bias","Heterogeneity"]**
**RT:CG**	**Some concerns**	**Low risk**	**No concerns**	**No concerns**	**Major concerns**	**No concerns**	**Very low**	**["Within-study bias","Heterogeneity"]**
**WBVE:CG**	**No concerns**	**Low risk**	**No concerns**	**Major concerns**	**No concerns**	**No concerns**	**Low**	**["Imprecision"]**
HIIT:RT	Some concerns	Low risk	No concerns	Major concerns	No concerns	Major concerns	Very low	["Within-study bias","Imprecision","Incoherence"]
MB/S:RT	Some concerns	Low risk	No concerns	No concerns	Major concerns	Major concerns	Very low	["Within-study bias","Heterogeneity","Incoherence"]
RT:WBVE	Some concerns	Low risk	No concerns	Major concerns	No concerns	No concerns	Very low	["Within-study bias","Imprecision"]

AE: Aerobic Exercise; AE-RT: Combined Aerobic and Resistance Training; RT: Resistance Training;MB/S:Mind-body exercise or stretching;HIIT: High-Intensity Interval Training;AQE:Aquatic Exercise; WBVE:Whole-Body Vibration Exercise;CG: Control group;The AQE:CG comparison for DBP was downgraded because the credible interval was wide and extended close to the null, indicating major concerns regarding imprecision. Major concerns were also assigned for heterogeneity after reassessment of the sparse AQE evidence. The overall confidence rating was therefore revised from high to very low.

Table S16. Summary of dose-response network meta-analysis result.

Category	Data point	Blood pressure	Effective dose (METs-min/week)	Optimal dose (METs-min/week)	Mean difference and 95% CrI (optimal dose)
Overall exercise	183	SBP	70-1900	1400	-6.70 mmHg (-9.18,-4.29)
	177	DBP	140-1800	1500	-4.20 mmHg (-6.52,-1.74)
Baseline SBP (＞130 mmHg)	92	SBP	70-2000	1700	-8.96 mmHg (-13.99,-3.74)
Baseline SBP (≤130 mmHg)	73	SBP	130-1100	920	-3.12 mmHg (-5.64,-0.68)
Baseline DBP (＞80 mmHg)	87	DBP	140-2000	1900	-6.71 mmHg (-11.10,-2.34)
Baseline DBP (≤80 mmHg)	75	DBP	NA	NA	NA
Length (＞12 weeks)	72	SBP	65-1500	1100	-5.95 mmHg (-9.16,-2.88)
Length (≤12 weeks)	111	SBP	70-2000	2000	-9.42 mmHg (-17.41,-1.51)
Length (＞12 weeks)	71	DBP	390-1100	1100	-3.45 mmHg (-6.73,-0.21)
Length (≤12 weeks)	109	DBP	210-2000	2000	-7.22 mmHg (-12.56,-1.67)
Sensitivity analysis	156	SBP	70-2000	1500	-7.30 mmHg (-10.43,-4.23)
	154	DBP	140-1800	1800	-4.80 mmHg (-9.10,-0.43)
AE	78	SBP	140-2000	1700	-6.27 mmHg (-10.54,-2.22)
	71	DBP	210-1600	1600	-3.54 mmHg (-6.89,-0.23)
AE-RT	23	SBP	200-1300	1300	-4.99 mmHg (-10.10,-0.11)
	18	DBP	590-790	790	-3.31 mmHg (-6.65,-0.03)
AQE	6	SBP	55-800	800	-11.63 mmHg (-19.69,-3.76)
	4	DBP	110-800	800	-7.73 mmHg (-14.74,-0.69)
HIIT	10	SBP	90-1300	1300	-9.53 mmHg (-15.60,-3.63)
	10	DBP	180-1300	1300	-5.84 mmHg (-11.34,-0.39)
MB/S	16	SBP	40-600	600	-9.78 mmHg (-14.87,-4.50)
	16	DBP	NA	NA	NA
RT	40	SBP	85-1200	1200	-6.29 mmHg (-11.99,-0.90)
	40	DBP	NA	NA	NA
WBVE	8	SBP	NA	NA	NA
	8	DBP	NA	NA	NA

NA: Not Applicable (no statistically significant effect observed); AE: Aerobic Exercise; AE-RT: Combined Aerobic and Resistance Training; RT: Resistance Training;MB/S:Mind-body exercise or stretching;HIIT: High-Intensity Interval Training; AQE:Aquatic Exercise;WBVE:Whole-Body Vibration Exercise;CG: Control group

Table S17. Illustrative translation of model-estimated dose ranges into weekly exercise minutes.

Blood pressure	Exercise modality	Energy expenditure (METs min)[a]	Recommended accumulation (min/week)	Optimal recommended Accumulation (min/week)	Certainty of evidence (grade)
Systolic blood pressure	Cycling	7 (code: 01016)[b]	20-285	245	**Very low**
	Jogging	4.8(code:12025)[c]	30-415	355	**Very low**
	Walking	3.5(code:17160)[d]	40-570	485	**Very low**
	Resistance training	3.5(code:02054)[e]	25-345	345	**Very low**
	Cycling combined with resistance training	7(code: 01016)<br>3.5(code:02054)	Cycling: 15-95；<br>Resistance training: 30-185	Cycling: 95；<br>Resistance training: 185	**Very low**
	Jogging combined with resistance training	4.8(code:12025)<br>3.5(code:02054)	Jogging: 20-135；<br>Resistance training: 30-185	Jogging: 135；<br>Resistance training: 185	**Very low**
	Yoga	2.3(code:02175)[f]	15-260	260	**Very low**
	Tai Chi	3.3(code:15670)[g]	10-180	180	**Very low**
	Dancing	4.8(code:02005)[h]	10-125	125	**Very low**
	Stretching	2.8(code:11593)[i]	15-215	215	**Very low**
	Swimming	5.8(code:18292)[j]	10-140	140	**Low**
	HIIT	11.0(code:02214)[k]	10-120	120	**Very low**
Diastolic blood pressure	Cycling	7 (code: 01016)[b]	30-230	230	**Very low**
	Jogging	4.8(code:12025)[c]	45-335	335	**Very low**
	Walking	3.5(code:17160)[d]	60-460	460	**Very low**

Cycling combined with resistance training	7(code: 01016)<br>3.5(code:02054)	Cycling: 40-55；<br>Resistance training: 85-115	Cycling: 55；<br>Resistance training: 115	**Very low**
Jogging combined with resistance training	4.8(code:12025)<br>3.5(code:02054)	Jogging: 60-80；<br>Resistance training: 85-115	Jogging: 80；<br>Resistance training: 115	**Very low**
Swimming	5.8(code:18292)[j]	20-140	140	**Very low**
HIIT	11.0(code:02214)[k]	15-120	120	**Very low**

Note: *Exercise intensity was coded based on the 2024 standardized physical activity intensity tables.[a]METs-min: Metabolic Equivalents of Task minutes. [b]Cycling: self-selected moderate pace. [c]Jogging, in place. [d]Walking for pleasure. [e]Resistance (weight) training, multiple exercises, 8-15 reps at varied resistance.[f]Yoga:general.[g]Taichi:Tai chi, qi gong, general.[h]Dance:aerobic dance, low impact, moderate effort.[i]Stretching:Sitting, teaching stretching or yoga, or light effort exercise classes.[j]Swimming: crawl, slow speed, 30-45 yards/minute, moderate effort.[k]HIIT:High intensity interval exercise, burpees, mountain climbers, squat jumps, Tabata, vigorous effort. 3-6 METs represents moderate-intensity activity, and >6 METs represents high-intensity activity, serving as a reference for absolute exercise intensity.All weekly-minute conversions are illustrative translations of model-estimated dose ranges and should not be interpreted as trial-validated exercise prescriptions or fixed clinical targets.

Reference

1. Arazi H, Asadi R, Taati B. Exercise training in thermo-mineral spring water has beneficial effects on hemodynamic and health-related factors in young-older hypertensive women: A randomized control trial. *Journal of Women & Aging*. 2020;32(3):279-291. doi:10.1080/08952841.2018.1547003

2. Arca EA, Martinelli B, Martin LC, Waisberg CB, Franco RJDS. Aquatic Exercise is as Effective as dry Land Training to Blood Pressure Reduction in Postmenopausal Hypertensive Women. *Physiotherapy Res Intl*. 2014;19(2):93-98. doi:10.1002/pri.1565

3. Asikainen T, Miilunpalo S, Kukkonen-Harjula K, et al. Walking trials in postmenopausal women: effect of low doses of exercise and exercise fractionization on coronary risk factors. *Scandinavian Med Sci Sports*. 2003;13(5):284-292. doi:10.1034/j.1600-0838.2003.00331.x

4. Cardoso CG, Rosas FC, Oneda B, et al. Aerobic training abolishes ambulatory blood pressure increase induced by estrogen therapy: A double blind randomized clinical trial. *Maturitas*. 2011;69(2):189-194. doi:10.1016/j.maturitas.2011.03.017

5. Elliott KJ, Sale C, Cable NT. Effects of resistance training and detraining on muscle strength and blood lipid profiles in postmenopausal women. *Br J Sports Med*. 2002;36(5):340-344. doi:10.1136/bjsm.36.5.340

6. Figueroa A, Park SY, Seo DY, Sanchez-Gonzalez MA, Baek YH. Combined resistance and endurance exercise training improves arterial stiffness, blood pressure, and muscle strength in postmenopausal women. *Menopause (New York, NY)*. 2011;18(9):980-984. doi:10.1097/gme.0b013e3182135442

7. Figueroa A, Kalfon R, Wong A. Whole-body vibration training decreases ankle systolic blood pressure and leg arterial stiffness in obese postmenopausal women with high blood pressure. *Menopause*. 2015;22(4):423-427. doi:10.1097/GME.0000000000000332

8. Hartman SJ, LaCroix AZ, Sears DD, et al. Impacts of Reducing Sitting Time or Increasing Sit-to-Stand Transitions on Blood Pressure and Glucose Regulation in Postmenopausal Women: Three-Arm Randomized Controlled Trial. *Circulation*. 2025;152(8):492-504. doi:10.1161/CIRCULATIONAHA.124.073385

9. Innes KE, Selfe TK. The Effects of a Gentle Yoga Program on Sleep, Mood, and Blood Pressure in Older Women with Restless Legs Syndrome (RLS): A Preliminary Randomized Controlled Trial. Evidence-Based Complementary and Alternative Medicine. 2012;2012:294058. doi:10.1155/2012/294058.

10. Latosik E, Zubrzycki IZ, Ossowski Z, et al. Physiological Responses Associated with Nordic-Walking Training in Systolic Hypertensive Postmenopausal Women. *Journal of Human Kinetics*. 2014;43(1):185-190. doi:10.2478/hukin-2014-0104

11. Lima AHRDA, Couto HE, Cardoso GA, Toscano LT, Silva AS, Mota MPG. Treinamento aeróbio não altera pressão arterial de mulheres menopausadas e com síndrome metabólica.

Arq Bras Cardiol. 2012;99(5):979-987. doi:10.1590/S0066-782X2012005000092

12. Myslivecek PR, Brown CA, Wolfe LA. Effects of Physical Conditioning on Cardiac Autonomic Function in Healthy Middle-Aged Women. *Can J Appl Physiol*. 2002;27(1):1-18. doi:10.1139/h02-001

13. Son WM, Sung KD, Cho JM, Park SY. Combined exercise reduces arterial stiffness, blood pressure, and blood markers for cardiovascular risk in postmenopausal women with hypertension. *Menopause*. 2017;24(3):262-268. doi:10.1097/GME.0000000000000765

14. Stachenfeld NS, Mack GW, DiPietro L, Morocco TS, Jozsi AC, Nadel ER. Regulation of blood volume during training in post-menopausal women. Medicine & Science in Sports & Exercise. 1998;30(1):92-98. doi:10.1097/00005768-199801000-00013.

15. Tudor-Locke C, Schuna JM, Swift DL, et al. Evaluation of Step-Counting Interventions Differing on Intensity Messages. *Journal of Physical Activity and Health*. 2020;17(1):21-28. doi:10.1123/jpah.2018-0439

16. Zhang D, Janjgava, T, Boutcher, SH, Boutcher Y. Cardiovascular response of postmenopausal women to 8 weeks of sprint interval training. *Eur J Appl Physiol*. 2019;119(4):981-989. doi:10.1007/s00421-019-04087-9

17. Zhou WS, Yang AL, Chen CN, et al. Effects of Acute Aquatic High-Intensity Intermittent Exercise on Blood Pressure and Arterial Stiffness in Postmenopausal Women with Different ACE Genotypes. *IJERPH*. 2022;19(15):8985. doi:10.3390/ijerph19158985

18. Akwa LG, Moses MO, Emikpe AO, et al. Lipid profile, cardiorespiratory function and quality of life of postmenopausal women improves with aerobic exercise. *jhse*. 2017;12(3). doi:10.14198/jhse.2017.123.14

19. Ammar T. Effects of aerobic exercise on blood pressure and lipids in overweight hypertensive postmenopausal women. *J Exerc Rehabil*. 2015;11(3):145-150. doi:10.12965/jer.150204

20. Anuradha, Kumar D. Effect of Yoga on Resting Blood Pressure among Postmenopausal Females with Type 2 Diabetes Mellitus – A Feasibility Randomized Controlled Trial. *Indian journal of physical therapy and research*. 2025;7(1):80-85. doi:10.4103/ijptr.ijptr_28_25

21. Azadpour N, Tartibian B, Koşar ŞN. Effects of aerobic exercise training on ACE and ADRB2 gene expression, plasma angiotensin II level, and flow-mediated dilation: a study on obese postmenopausal women with prehypertension. Menopause. 2017;24(3):269-277. doi:10.1097/GME.0000000000000762

22. Batista JP, Tavares JB, Gonçalves LF, et al. Mat Pilates training reduces blood pressure in both well-controlled hypertensive and normotensive postmenopausal women: a controlled clinical trial study. *Clinical and Experimental Hypertension*. 2022;44(6):548-556. doi:10.1080/10641963.2022.2079670

23. Binder EF, Birge SJ, Kohrt WM. Effects of Endurance Exercise and Hormone Replacement Therapy on Serum Lipids in Older Women. *J American Geriatrics Society*. 1996;44(3):231-236. doi:10.1111/j.1532-5415.1996.tb00907.x

24. Boutcher YN, Boutcher SH, Yoo HY, Meerkin JD. The Effect of Sprint Interval Training on Body Composition of Postmenopausal Women. Medicine & Science in Sports & Exercise. 2019;51(7):1413-1419. doi:10.1249/MSS.0000000000001919.

25. Casey DP, Pierce GL, Howe KS, Mering MC, Braith RW. Effect of Resistance Training on Arterial Wave Reflection and Brachial Artery Reactivity in Normotensive Postmenopausal Women. European Journal of Applied Physiology. 2007;100(4):403-408. doi:10.1007/s00421-007-0447-2.

26. Colado JC, Triplett NT, Tella V, Saucedo P, Abellán J. Effects of aquatic resistance training on health and fitness in postmenopausal women. *Eur J Appl Physiol*. 2009;106(1):113-122. doi:10.1007/s00421-009-0996-7

27. Conceição M, Bonganha, Vechin, et al. Sixteen weeks of resistance training can decrease the risk of metabolic syndrome in healthy postmenopausal women. *CIA*. Published online September 2013:1221. doi:10.2147/CIA.S44245

28. Corrick KL, Hunter GR, Fisher G, Glasser SP. Changes in Vascular Hemodynamics in Older Women Following 16 Weeks of Combined Aerobic and Resistance Training. *J of Clinical Hypertension*. 2013;15(4):241-246. doi:10.1111/jch.12050

29. Dalleck LC, Allen BA, Hanson BA, Borresen EC, Erickson ME, De Lap SL. Dose-Response Relationship between Moderate-Intensity Exercise Duration and Coronary Heart Disease Risk Factors in Postmenopausal Women. *Journal of Women's Health*. 2009;18(1):105-113. doi:10.1089/jwh.2008.0790

30. El-Refaye GE, Younis HA. The effect of 12 weeks of resistive exercises versus aerobic exercises in overweight hypertensive postmenopausal women. *Bull Fac Phys Ther*. 2019;24(1):40-48. doi:10.4103/bfpt.bfpt_19_18

31. Fairey AS, Courneya KS, Field CJ, et al. Effect of exercise training on C-reactive protein in postmenopausal breast cancer survivors: A randomized controlled trial. *Brain, Behavior, and Immunity*. 2005;19(5):381-388. doi:10.1016/j.bbi.2005.04.001

32. Ferreira MLV, Castro A, De Oliveira Nunes SG, et al. Hypotensive effects of exercise training: are postmenopausal women with hypertension non-responders or responders? *Hypertens Res*. 2024;47(8):2172-2182. doi:10.1038/s41440-024-01721-8

33. Figueroa A, Kalfon R, Madzima TA, Wong A. Effects of whole-body vibration exercise training on aortic wave reflection and muscle strength in postmenopausal women with prehypertension and hypertension. *J Hum Hypertens*. 2014;28(2):118-122. doi:10.1038/jhh.2013.59

34. Garnier S, Joffroy S, Thon B, Auneau G, Mauriège P. Two-year follow-up of a brisk walking programme on fitness, perceived health and physical activity engagement in postmenopausal women. *Health Education Journal*. 2024;83(6):638-649. doi:10.1177/00178969241269016

35. Gelecek N, İlçin N, Subaşi SS, Acar S, Demir N, Örmen M. The Effects of Resistance Training on Cardiovascular Disease Risk Factors in Postmenopausal Women: A Randomized-Controlled Trial. *Health Care for Women International*. 2012;33(12):1072-1085. doi:10.1080/07399332.2011.645960

36. Gerage A, Forjaz CL, Nascimento M, Januário RS, Polito M, Cyrino E. Cardiovascular Adaptations to Resistance Training in Elderly Postmenopausal Women. *Int J Sports Med*. 2013;34(09):806-813. doi:10.1055/s-0032-1331185

37. Ghadamyari N, Zolfaghari MR, Tolouei Azar J, Fattahi A. The effect of 8 weeks of endurance and resistance exercises on the serum levels of FGF23 and s-Klotho in type 2 diabetic women. *Int J Diabetes Dev Ctries*. 2025;45(2):472-479. doi:10.1007/s13410-024-01343-3

38. Gholamrezai S, Zali M, Shabani R, Hoseini R. Effect of 8 Weeks of Aerobic Exercise on Body Composition and Blood Pressure in Postmenopausal Women. Physical Education of Students. 2014;18(5):74-78. doi:10.15561/20755279.2014.0514.

39. Henríquez S, Monsalves-Alvarez M, Jimenez T, et al. Effects of Two Training Modalities on Body Fat and Insulin Resistance in Postmenopausal Women. Journal of Strength and Conditioning Research. 2017;31(11):2955-2964. doi:10.1519/JSC.0000000000002089.

40. Jaime SJ, Maharaj A, Alvarez-Alvarado S, Figueroa A. Impact of low-intensity resistance and whole-body vibration training on aortic hemodynamics and vascular function in postmenopausal women. *Hypertens Res*. 2019;42(12):1979-1988. doi:10.1038/s41440-019-0328-1

41. Jeon K, Lee S, Hwang MH. Effect of combined circuit exercise on arterial stiffness in hypertensive postmenopausal women: a local public health center-based pilot study. *Menopause*. 2018;25(12):1442-1447. doi:10.1097/GME.0000000000001154

42. Kamal WM, Ali EA, Radwan MA, Ahmed ZS, Mohamed DM. Effect of whole-body vibration on blood pressure in obese postmenopausal women. A randomized controlled study. *Fiz Pol*. 2024;24(2):35-41. doi:10.56984/8ZG5608H7W

43. Kazemi SS, Heidarianpour A, Shokri E. Effect of resistance training and high-intensity interval training on metabolic parameters and serum level of Sirtuin1 in postmenopausal women with metabolic syndrome: a randomized controlled trial. *Lipids Health Dis*. 2023;22(1):177. doi:10.1186/s12944-023-01940-x

44. Kim JW, Kim DY. Effects of Aerobic Exercise Training on Serum Sex Hormone Binding Globulin, Body Fat Index, and Metabolic Syndrome Factors in Obese Postmenopausal Women. *Metabolic Syndrome and Related Disorders*. 2012;10(6):452-457. doi:10.1089/met.2012.0036

45. Lee JA, Kim JW, Kim DY. Effects of yoga exercise on serum adiponectin and metabolic syndrome factors in obese postmenopausal women. *Menopause*. 2012;19(3):296-301. doi:10.1097/gme.0b013e31822d59a2

46. Lee SH, Scott SD, Pekas EJ, Lee S, Lee SH, Park SY. Taekwondo training reduces blood catecholamine levels and arterial stiffness in postmenopausal women with stage-2 hypertension: randomized clinical trial. *Clinical and Experimental Hypertension*. 2019;41(7):675-681. doi:10.1080/10641963.2018.1539093

47. Lindheim S r., Notelovitz M, Feldman E b., Larsen S, Khan F y. The independent effects of exercise and estrogen on lipids and lipoproteins in postmenopausal women. *International Journal of Gynecology & Obstetrics*. 1994;47(1):88-89. doi:10.1016/0020-7292(94)90488-X

48. Babiloni-Lopez C, Gargallo P, Juesas A, et al. Long-Term Effects of Microfiltered Seawater and Resistance Training with Elastic Bands on Hepatic Parameters, Inflammation, Oxidative Stress, and Blood Pressure of Older Women: A 32-Week, Double-Blinded, Randomized, Placebo-Controlled Trial. *Healthcare*. 2024;12(2):204. doi:10.3390/healthcare12020204

49. Lyall GK, Birk GK, Harris E, et al. Efficacy of interval exercise training to improve vascular health in sedentary postmenopausal females. *Physiological Reports*. 2022;10(16). doi:10.14814/phy2.15441

50. Maeda S, Tanabe T, Otsuki T, et al. Moderate Regular Exercise Increases Basal Production of Nitric Oxide in Elderly Women. *Hypertens Res*. 2004;27(12):947-953. doi:10.1291/hypres.27.947

51. Matsubara T, Miyaki A, Akazawa N, et al. Aerobic exercise training increases plasma Klotho levels and reduces arterial stiffness in postmenopausal women. *American Journal of Physiology-Heart and Circulatory Physiology*. 2014;306(3):H348-H355. doi:10.1152/ajpheart.00429.2013

52. McGavock J, Mandic S, Lewanczuk R, Koller M, Muhll IV, Quinney A, Taylor D, Welsh R, Haykowsky M. Cardiovascular Adaptations to Exercise Training in Postmenopausal Women with Type 2 Diabetes Mellitus. Cardiovascular Diabetology. 2004;3:3. doi:10.1186/1475-2840-3-3.

53. Miura H, Nakagawa E, Takahashi Y. Influence of group training frequency on arterial stiffness in elderly women. *Eur J Appl Physiol*. 2008;104(6):1039-1044. doi:10.1007/s00421-008-0860-1

54. Moreau KL. The Effects of Walking Volume on Blood Pressure in Hypertensive Postmenopausal Women [doctoral dissertation]. Knoxville, TN: University of Tennessee; 1999.

55. Moreau KL, DeGarmo R, Langley J, et al. Increasing daily walking lowers blood pressure in postmenopausal women. Medicine and Science in Sports and Exercise. 2001;33(11):1825-1831. doi:10.1097/00005768-200111000-00005.

56. Nemoto K, Gen-no H, Masuki S, Okazaki K, Nose H. Effects of High-Intensity Interval Walking Training on Physical Fitness and Blood Pressure in Middle-Aged and Older People. Mayo Clinic Proceedings. 2007;82(7):803-811. doi:10.4065/82.7.803.

57. Nishida Y, Tanaka K, Hara M, et al. Effects of home-based bench step exercise on inflammatory cytokines and lipid profiles in elderly Japanese females: A randomized controlled trial. *Archives of Gerontology and Geriatrics*. 2015;61(3):443-451. doi:10.1016/j.archger.2015.06.017

58. Nunes PRP, Silva TRGB, Carneiro MAS, Martins FM, Souza AP, Orsatti FL. Functional high-intensity interval training is not equivalent when compared to combined training for blood pressure improvements in postmenopausal women: a randomized controlled trial. *Clinical and Experimental Hypertension*. 2022;44(2):127-133. doi:10.1080/10641963.2021.2001481

59. Nuri R, Kordi M, Moghaddasi M, et al. Effect of combination exercise training on metabolic syndrome parameters in postmenopausal women with breast cancer. *J Can Res Ther*. 2012;8(2):238. doi:10.4103/0973-1482.98977

60. Oneda B, Cardoso CG, Forjaz CLM, et al. Effects of estrogen therapy and aerobic training on sympathetic activity and hemodynamics in healthy postmenopausal women: a double-blind randomized trial. *Menopause*. 2014;21(4):369-375. doi:10.1097/GME.0b013e31829d2a00

61. Paolillo FR, Corazza AV, Borghi-Silva A, Parizotto NA, Kurachi C, Bagnato VS. Infrared LED irradiation applied during high-intensity treadmill training improves maximal exercise tolerance in postmenopausal women: a 6-month longitudinal study. *Lasers Med Sci*. 2013;28(2):415-422. doi:10.1007/s10103-012-1062-y

62. Pospieszna B, Karolkiewicz J, Tarnas J, Lewandowski J, Laurentowska M, Pilaczyńska-Szcześniak Ł. Influence of 12-week Nordic Walking training on biomarkers of endothelial function in healthy postmenopausal women. *J Sports Med Phys Fitness*. 2017;57(9). doi:10.23736/S0022-4707.16.06528-2

63. Rahnama N, Nouri R, Rahmaninia F, Damirchi A, Emami H. The effects of exercise training on maximum aerobic capacity, resting heart rate, blood pressure and anthropometric variables of postmenopausal women with breast cancer. *J Res Med Sci*. 2010;15(2):78-83.

64. Ready AE, Naimark B, Ducas J, et al. Influence of walking volume on health benefits in women post-menopause. *Med Sci Sports Exerc*. 1996;28(9):1097-1105. doi:10.1097/00005768-199609000-00004

65. Reis JG, Costa GC, Schmidt A, Ferreira CHJ, Abreu DCC. Do muscle strengthening exercises improve performance in the 6-minute walk test in postmenopausal women? *Rev bras fisioter*. 2012;16(3):236-240. doi:10.1590/S1413-35552012005000022

66. Rezende Barbosa MP, Vanderlei LC, Neves LM, et al. Functional training in postmenopause: Cardiac autonomic modulation and cardiorespiratory parameters, a randomized trial.

Geriatrics Gerontology Int. 2019;19(8):823-828. doi:10.1111/ggi.13690

67. Santa-Clara H, Szymanski L, Fernhall B o. Effect of exercise training on blood pressure in postmenopausal Caucasian and African-American women. *The American Journal of Cardiology*. 2003;91(8):1009-1011. doi:10.1016/S0002-9149(03)00128-0

68. Sardeli AV, Castro A, Gadelha VB, et al. Metabolomic Response throughout 16 Weeks of Combined Aerobic and Resistance Exercise Training in Older Women with Metabolic Syndrome. *Metabolites*. 2022;12(11):1041. doi:10.3390/metabo12111041

69. Sattar M, Esfarjani F, Nezakat-Alhosseini M, Kordavani L. Effect of Aquatic Resistance Training on Blood Pressure and Physical Function of Postmenopausal Women. ARYA Atherosclerosis Journal. 2012;8(Special Issue in National Hypertension Treatment):S178-S182.

70. Sénéchal M, Bouchard DR, Dionne IJ, Brochu M. The effects of lifestyle interventions in dynapenic-obese postmenopausal women. *Menopause*. 2012;19(9):1015-1021. doi:10.1097/gme.0b013e318248f50f

71. Seo J, Park HY, Jung WS, et al. Effects of 12 Weeks of Resistance Training on Body Composition, Muscle Hypertrophy and Function, Blood Lipid Level, and Hemorheological Properties in Middle-Aged Obese Women. *Rev Cardiovasc Med*. 2023;24(7):196. doi:10.31083/j.rcm2407196

72. Seo DI, Jun TW, Park KS, Chang H, So WY, Song W. 12 Weeks of Combined Exercise Is Better Than Aerobic Exercise for Increasing Growth Hormone in Middle-Aged Women. *International Journal of Sport Nutrition and Exercise Metabolism*. 2010;20(1):21-26. doi:10.1123/ijsnem.20.1.21

73. Serrano-Guzmán M, Aguilar-Ferrándiz ME, Valenza CM, Ocaña-Peinado FM, Valenza-Demet G, Villaverde-Gutiérrez C. Effectiveness of a flamenco and sevillanas program to enhance mobility, balance, physical activity, blood pressure, body mass, and quality of life in postmenopausal women living in the community in Spain: a randomized clinical trial. *Menopause*. 2016;23(9):965-973. doi:10.1097/GME.0000000000000652

74. Shabani A, Shabani R, Dalili S, Hassanzadeh Rad A. The effect of concurrent endurance and resistance training on cardio-respiratory capacity and cardiovascular risk markers among sedentary overweight or obese post-menopausal women. *J Nurs Midwifery Sci*. 2018;5(4):123. doi:10.4103/JNMS.JNMS_34_18

75. Shakoor E, Qassemian A, Koushkie Jahromi M, Mehrez A. Effects of 8-Week Aerobic Exercise Training on Vascular Endothelial Growth Factor among Postmenopausal Women. International Journal of Applied Exercise Physiology. 2016;5(4):1-10.

76. Shaw BS, Gouveia M, McIntyre S, Shaw I. Anthropometric and cardiovascular responses to hypertrophic resistance training in postmenopausal women. *Menopause*. 2016;23(11):1176-1181. doi:10.1097/GME.0000000000000687

77. Sobrinho ACS, Benjamim CJR, Luciano De Almeida M, et al. Fourteen weeks of multicomponent training associated with flexibility training modifies postural alignment, joint range of motion and modulates blood pressure in physically inactive older women: a randomized clinical trial. *Front Physiol*. 2023;14:1172780. doi:10.3389/fphys.2023.1172780

78. Son WM, Pekas EJ, Park SY. Twelve weeks of resistance band exercise training improves age-associated hormonal decline, blood pressure, and body composition in postmenopausal women with stage 1 hypertension: a randomized clinical trial. *Menopause*. 2020;27(2):199-207. doi:10.1097/GME.0000000000001444

79. Son WM, Park JJ. Resistance Band Exercise Training Prevents the Progression of Metabolic Syndrome in Obese Postmenopausal Women. *jsportscimed*. Published online March 15, 2021:291-299. doi:10.52082/jssm.2021.291

80. Staffileno BA, Braun LT, Rosenson RS. The Accumulative Effects of Physical Activity in Hypertensive Post-Menopausal Women. European Journal of Cardiovascular Prevention & Rehabilitation. 2001;8(5):283-290. doi:10.1177/174182670100800507.

81. Sugawara J, Akazawa N, Miyaki A, et al. Effect of endurance exercise training and curcumin intake on central arterial hemodynamics in postmenopausal women: pilot study. *American Journal of Hypertension*. 2012;25(6):651-656. doi:10.1038/ajh.2012.24

82. Swift DL, Earnest CP, Katzmarzyk PT, Rankinen T, Blair SN, Church TS. The effect of different doses of aerobic exercise training on exercise blood pressure in overweight and obese postmenopausal women. *Menopause*. 2012;19(5):503-509. doi:10.1097/gme.0b013e318238ea66

83. Taha MM. Effect of High Intensity Interval Training on Endothelial Function in Postmenopausal Hypertensive Patients:Randomized Controlled Trial. *International Journal of Physiotherapy*. 2016;3(1). doi:10.15621/ijphy/2016/v3i1/88908

84. Tanahashi K, Akazawa N, Miyaki A, et al. Aerobic Exercise Training Decreases Plasma Asymmetric Dimethylarginine Concentrations With Increase in Arterial Compliance in Postmenopausal Women. *American Journal of Hypertension*. 2014;27(3):415-421. doi:10.1093/ajh/hpt217

85. Khalid T, Nesreen E, Ramadhan O. Effects of exercise training on postmenopausal hypertension: implications on nitric oxide levels. *Med J Malaysia*. 2013;68(6):459-464.

86. Wang X, Han Y, Li H, Wang X, Wang G. Impact of a Precision Intervention for Vascular Health in Middle-Aged and Older Postmenopausal Women Using Polar Heart Rate Sensors: A 24-Week RCT Study Based on the New Compilation of Tai Chi (Bafa Wubu). *Sensors*. 2024;24(17):5832. doi:10.3390/s24175832

87. Wong A, Figueroa A. Eight weeks of stretching training reduces aortic wave reflection magnitude and blood pressure in obese postmenopausal women. *J Hum Hypertens*. 2014;28(4):246-250. doi:10.1038/jhh.2013.98

88. Wong A, Alvarez-Alvarado S, Kinsey AW, Figueroa A. Whole-Body Vibration Exercise Therapy Improves Cardiac Autonomic Function and Blood Pressure in Obese Pre- and Stage 1 Hypertensive Postmenopausal Women. *The Journal of Alternative and Complementary Medicine*. 2016;22(12):970-976. doi:10.1089/acm.2016.0124

89. Wong A, Figueroa A, Son WM, Chernykh O, Park SY. The effects of stair climbing on arterial stiffness, blood pressure, and leg strength in postmenopausal women with stage 2 hypertension. *Menopause*. 2018;25(7):731-737. doi:10.1097/GME.0000000000001072

90. Wong A, Kwak YS, Scott SD, et al. The effects of swimming training on arterial function, muscular strength, and cardiorespiratory capacity in postmenopausal women with stage 2 hypertension. *Menopause*. 2019;26(6):653-658. doi:10.1097/GME.0000000000001288

91. Yoshizawa M, Maeda S, Miyaki A, et al. Additive beneficial effects of lactotripeptides and aerobic exercise on arterial compliance in postmenopausal women. *American Journal of Physiology-Heart and Circulatory Physiology*. 2009;297(5):H1899-H1903. doi:10.1152/ajpheart.00433.2009
